# The fermented cabbage metabolome and its protection against cytokine-induced intestinal barrier disruption of Caco-2 monolayers

**DOI:** 10.1128/aem.02234-24

**Published:** 2025-04-07

**Authors:** Lei Wei, Maria L. Marco

**Affiliations:** 1 Department of Food Science and Technology, University of California Davis 117239, Davis, California, USA; Universita degli Studi di Napoli Federico II, Portici, Italy

**Keywords:** fermented food, lactobacilli, probiotic, intestinal barrier function, metabolome, gut inflammation, ILA, PLA

## Abstract

Fermented vegetables, such as fermented cabbage (sauerkraut), have garnered growing interest for their associations with a myriad of health benefits. However, the mechanistic details underlying the outcomes of consuming these foods require further investigation. This study examined the capacity of soluble metabolites in laboratory-scale and commercial-fermented cabbage to protect against disruption of polarized Caco-2 monolayers by interferon gamma (IFN-γ) and tumor necrosis factor-alpha (TNF-α). Laboratory-scale ferments (LSF) were prepared with and without the addition of *Lactiplantibacillus plantarum* NCIMB8826R (LP8826R) and sampled after 7 and 14 days of incubation. Trans-epithelial electrical resistance (TER) and paracellular permeability to fluorescein isothiocyanate (FITC)-dextran revealed that fermented cabbage, but not raw cabbage or brine, protected against cytokine-induced damage to the Caco-2 monolayers. Barrier-protective effects occurred despite increased IL-8 production following cytokine exposure. Metabolomic analyses performed using gas and liquid chromatography resulted in the identification of 149 and 333 metabolites, respectively. Significant differences were found between raw and fermented cabbage. LSF metabolomes changed over time, and the profiles of LSF with LP8826R best resembled the commercial product. Overall, fermentation resulted in lower carbohydrate and increased lactic acid, lipid, amino acid derivative (including D-phenyl-lactate [D-PLA], indole-3-lactate [ILA], and γ-aminobutyric acid [GABA]), and phenolic compound concentrations. Lactate, D-PLA, and ILA tested individually and combined only partially protected against cytokine-induced TER reductions and increases in paracellular permeability of Caco-2 monolayers. The findings show that intestinal barrier-protective compounds are consistently enriched during cabbage fermentations, irrespective of the scale or microbial additions, which may contribute to the health-promoting potential of these foods.

**IMPORTANCE:**

Fermented vegetables are increasingly associated with health benefits. However, the importance of microbial transformations to foods during the fermentation process remains to be determined. We found that the metabolites in spontaneously fermented cabbage protected polarized intestinal epithelial cells against damage induced by proinflammatory cytokines. Cabbage fermentations resulted in consistent metabolome profiles enriched in bioactive compounds known to be made by beneficial members of the human gut microbiome, including D-phenyl-lactate (D-PLA) and indole-3-lactate (ILA). The metabolomes were distinct from raw cabbage and were further differentiated between commercial and lab ferments, sampling time, and the presence of an exogenous *Lactiplantibacillus plantarum* strain. Because only partial protection against intestinal barrier disruption was found when individual metabolites (D-PLA, ILA, and lactate) were applied, the findings indicate that the complex mixture of metabolites in a cabbage fermentation offers advantages over single metabolites to benefit intestinal health.

## INTRODUCTION

Fermented vegetables have been staples of the human diet for thousands of years (1). Besides imparting distinct organoleptic characteristics to foods, the process of fermentation improves the preservation and safety of easily perishable and highly nutritive raw vegetables through microbial production of organic acids, bacteriocins, alcohols, and other fermentation end-products along with the transformation of the nutrients in the ingredients (2, 3). Recently, human (4–8) and model animal (9, 10) studies have shown that fermented vegetables can benefit human health. However, the mechanistic details underpinning these outcomes remain to be determined.

Fermented cabbage is a popular fermented vegetable widely consumed across the world and is known by different names (e.g., sauerkraut, suan cai, curtido, etc.) (11). The basic recipe is essentially mixing shredded cabbage with 2%–3% (wt/wt) sodium chloride and incubating the mixture with minimal exposure to ambient oxygen at room temperature for 2–3 weeks (1). During cabbage fermentation, the lack of oxygen and the presence of salt select for lactic acid bacteria (LAB). LAB encompass a group of phylogenetically related bacteria in the Bacillota (formerly Firmicutes) phylum that were traditionally grouped together because of their saccharolytic fermentation energy conservation metabolism, resulting in lactic acid as the only or the main (> 50%) end-product (12). Culture-dependent and culture-independent analyses have shown that the dominant LAB in cabbage fermentations change over time, with initial enrichments of heterofermentative species, such as *Leuconostoc mesenteroides* and *Weissella* spp., followed by *Lactiplantibacillus plantarum* and *Levilactobacillus brevis* starting a couple of days later (13, 14). It is well-understood that LAB break down sugars and other compounds and produce lactic and acetic acids, mannitol, and CO_2_, as the primary secreted metabolic end-products, sometimes reaching 2% (vol/wt) or more in the ferments (15). Besides their antimicrobial effects and contributions to the sensory properties of fermented foods, compounds such as lactic acid and acetic acid are of interest because of their association with improving intestinal barrier function (16–19), immune function (20, 21), and metabolic health (e.g., fasting blood glucose and insulin sensitivity) (22–24).

Compared with raw cabbage, LAB-guided cabbage fermentations also have higher concentrations of phenolic compounds (polyphenols, phenolic acids) (25, 26), carotenoids (26), glucosinolate breakdown products (ascorbigen, indole-3-carbinol, and isothiocyanates) (27, 28), and other bioactive metabolites (29). The amino acid derivatives, phenyl-lactic acid (PLA) (30) and indole-3-lactic acid (ILA) (31), are of particular interest because cell culture and animal model studies reported intestinal barrier-protective properties following administration or production of microbiota-associated PLA (32, 33) and ILA (34–36). These effects are conferred through the activation of signaling pathways involving PPAR-γ (PLA [32]) and AHR (ILA [37]). PLA and ILA together with organic acids like lactic acid (3, 38) are likely important for the observed immunomodulatory (39), antioxidant (27), and anti-carcinogenic properties (6, 15, 29) of fermented cabbage. Despite these potential benefits, only a few randomized controlled trials investigating the health benefits of fermented cabbage consumption have been performed, and most of those studies focused on kimchi (8, 40, 41). For sauerkraut, a pilot study reported that both pasteurized and unpasteurized sauerkraut significantly improved irritable bowel syndrome (IBS) symptoms (*P* < 0.04) (5).

Of particular interest is the capacity of fermented cabbage and related foods to alter intestinal barrier function. The intestinal epithelium serves as a selectively permeable barrier, which allows the absorption of fluids and nutrients while limiting the translocation of microorganisms, antigens, and other toxic compounds into the sterile serosal circulation of the host from the gut lumen (42, 43). A wide range of substances, such as cytokines, growth factors, dietary components, and microbial metabolites, can influence intestinal barrier integrity and function by modulating junctional complexes (42). For instance, the proinflammatory cytokine tumor necrosis factor-alpha (TNF-α) increases the expression of myosin light chain kinase (MLCK), which then causes the re-organization of the peri-junctional actin and tight junction proteins (e.g., occludin and ZO-1), resulting in increased paracellular permeability (44). Cell culture models (e.g., Caco-2, T84, and HT-29) (45, 46) have played a key role in facilitating a functional and mechanistic understanding of the association between disrupted intestinal barrier integrity and numerous inflammatory and metabolic diseases (e.g., inflammatory bowel disease and obesity) and the testing of food-associated compounds and pharmaceuticals that may be useful for supporting barrier function (42, 47).

To better understand the potential of fermented cabbage to impact intestinal function, we evaluated cell-free preparations of raw cabbage and commercial and laboratory-scale-fermented cabbage (sauerkraut). Homogenates of (fermented) cabbage prepared from an estimated 10 g serving size were tested for their capacity to protect against pro-inflammatory cytokine (IFN-γ and TNF-α)-induced damage in a polarized Caco-2 cell model. Some laboratory fermentations also incorporated *L. plantarum* NCIMB8826R (LP8826R), shown to increase the localization of the tight junction protein ZO-1 in healthy individuals (48) as well as induce intestinal epithelial repair (49–51) and exert anti-inflammatory (52) and anti-obesogenic (53) effects in animal models. To begin to understand the differences between raw cabbage and fermented cabbage, metabolites were quantified with (un)targeted metabolomics using gas chromatography-time of-flight mass spectrometry (GC-TOF/MS) and reverse-phase liquid chromatography high-resolution tandem mass spectrometry (RP-LC-HRMS/MS). Based on those findings, lactate, D-PLA, and ILA were investigated for barrier-protective capacities in the polarized Caco-2 cell model.

## MATERIALS AND METHODS

### Bacterial strain growth conditions and preparation of the inoculum for cabbage fermentations

Isolation of the rifampicin-resistant mutant LP8826R was described previously (52). LP8826R was routinely grown at 37°C in lactobacilli de Man, Rogosa, and Sharpe (MRS) medium (Becton-Dickinson, Franklin Lakes, NJ, USA). When appropriate, 50 µg/mL rifampicin (Thermo Fisher Scientific, Waltham, MA, USA) was added to the MRS for LP8826R enumeration. For inoculation into sauerkraut fermentations, LP8826R was incubated in MRS for 24 h, collected by centrifugation at 9,000 × *g* for 3 min at room temperature, washed twice in sterile phosphate-buffered saline (PBS; 137 mM NaCl, 2.7 mM KCl, 4.3 mM Na_2_HPO_4_*7H_2_O, 1.4 mM KH_2_PO_4_, pH 7.2), and then suspended in sterile PBS.

### Laboratory-scale fermentations

Cabbages (*Brassica oleracea* var. capitata f. alba), purchased from local grocery stores, were shredded using a grater, mixed with 2% canning salt (wt/wt) (Morton Salt, Chicago, IL, USA), dispensed into 28 oz sterile WhirlPak bags with a 330-µm filter (Whirl-Pak, Pleasant Prairie, WI, USA) and covered with a 2% (wt/wt) brine solution of canning salt until all pieces were submerged. After pressing out as much air as possible, the WhirlPak bag containers were closed by rolling the tabs at least three times. Six WhirlPak bag containers with the cabbage and salt mixture were incubated at 22°C. After 3 days, LP8826R in 1.75 to 2 mL PBS was added to half of the containers (*n* = 3) to reach 5 × 10^7^ CFU/g. The same volume of PBS was added to the other laboratory ferments. The inoculation of LP8826R on day 3 was to mimic the succession of heterofermentative LAB by homofermentative LAB such as *L. plantarum* in fruit and vegetable fermentations (54, 55). The LSF were performed on two separate dates, once with a total fermentation time of 14 days, and the other for 21 days. Brine pH (SevenEasy pH meter, Mettler-Toledo, Columbus, OH, USA) and salinity (SPER Scientific, Scottsdale, AZ, USA) were measured for the first 7 days and then at the end of the study.

### Commercial sauerkraut

Containers (*n* = 6) of commercial sauerkraut from the same brand were purchased from local supermarkets between December 2021 and October 2022 (Table 1). They were stored at 4°C and sampled within 3 days of purchase.

**TABLE 1:** Parameters of commercial sauerkraut sampled

Lot no.	Best by date	Sampling date	Brine salinity (% wt/vol)	Homogenate pH	LAB (log_10_ CFU/mL)^*a*^
ND	ND	Dec 08, 2021	ND^*b*^	3.37	ND
ND	ND	Nov 12, 2021	ND	3.20	ND
2022234	012623	Sep 28, 2022	2.67	3.37	6.60
2022209	122922	Sep 28, 2022	2.75	3.30	6.95
2022234	012623	Sep 28, 2022	2.74	3.36	6.99
2022166	111922	Oct 09, 2022	2.53	3.42	1.90

^*a*^ Estimated on MRS laboratory culture medium.

^*b*^ ND, not determined.

### Intestinal epithelial cell culture maintenance

Caco-2 cells ATCC HTB-37 were purchased from the American Type Cell Culture (Rockville, MD, USA) and grown in DMEM as previously described (56). Dulbecco’s Modified Eagle Medium (DMEM), fetal bovine serum (FBS), sodium pyruvate (100×), nonessential amino acids (100×), GlutaMax (100×), penicillin/streptomycin 10,000 U/mL, Trypsin-EDTA (0.25%, 1×), and sterile Dulbecco’s phosphate buffer saline (D-PBS, pH 7.4, no calcium, no magnesium) were obtained from Gibco (Life Technologies, Carlsbad, CA, USA). Briefly, the Caco-2 cells were incubated in complete DMEM containing 20% FBS, 1 mM sodium pyruvate, 0.1 mM nonessential amino acids, 2 mM GlutaMax, and 1% vol/vol penicillin/streptomycin at 37°C in 10% CO_2_, split using Trypsin-EDTA, and passaged in 75 cm^2^ culture flasks (Fisher Scientific, Waltham, MA, USA) until they were ready for seeding.

### Preparation of (fermented) cabbage homogenates for bacterial enumeration and application onto Caco-2 monolayers

Approximately 10 g cabbage (either fermented or raw) and 1 mL brine were collected and homogenized in sterile WhirlPak bags with a 330-µm filter using a rubber mallet. The homogenates were then serially diluted in PBS and plated on MRS agar containing 25 µg/mL natamycin (Dairy Connection, WI, USA), an antifungal compound, for total bacterial cell counts and MRS agar containing both 25 µg/mL natamycin and 50 µg/mL rifampicin for LP8826R enumeration.

For experiments with Caco-2 cells, cabbage homogenates were centrifuged at 10,000 × *g* for 10 min at 4°C. The supernatant was collected and adjusted to a pH of 7.4 using either 1 M or 6 M sodium hydroxide (NaOH) prior to passing through a 0.22 µm, surfactant-free cellulose acetate (SFCA) filter (Globe Scientific, Mahwah, NJ, USA). Homogenate pH was justed in order to simulate the pH environment of the small intestine (between pH 6.1 and pH 7.5) in the Caco-2 monolayer model (57).

To determine the appropriate cabbage homogenate concentration for use on the Caco-2 monolayers, a subset of samples, specifically LSF after 7 days of fermentation (3 samples) and commercial sauerkraut (one sample), was tested at a level of 1%, 10%, or 50% (vol/vol) in supplemented DMEM (containing 1 mM sodium pyruvate, 0.1 mM nonessential amino acids, and 2 mM GlutaMax) in the Caco-2 Transwell model. Based on those findings, cabbage homogenates at 10% (vol/vol) in supplemented DMEM were selected for further testing (*n* = 6 replicates per sample type). A 2.91% (wt/vol) solution of NaCl was made to match the salinity of commercial fermented cabbage homogenates, diluted to 10% (vol/vol) in DMEM, and used to examine the impact of salt on the Caco-2 cell monolayers. The prepared homogenates and salt solution were stored at −80°C until use.

### Lactate, ILA, and D-PLA preparation for application onto Caco-2 monolayers

DL-lactic acid (W261106-1KG-K), DL-indole-3-lactic acid (ILA; I5508-250MG-A), and D-(+)−3-phenyllactic acid (D-PLA; 376906–5G) were obtained from Sigma-Aldrich (St. Louis, MO, USA). Lactate and D-PLA were suspended in sterile water, and ILA was mixed with 0.052% vol/vol dimethyl sulfoxide (DMSO). All three compounds were prepared in concentrations 10-fold higher than intended for use in cell culture. To ensure that D-PLA and ILA were dissolved, the solutions were sonicated for 10 min at 40 kHz in a Branson 8510 Ultrasonic Cleaner bath (Branson Ultrasonics Corp., Danbury, CT, USA). The metabolite solutions were then adjusted to a pH of 7.40 ± 0.04 with NaOH and diluted in supplemented DMEM to reach a final concentration of 50 mM lactate, 60 µM D-PLA, and 25 µM ILA (0.0052% [vol/vol] DMSO), both separately and combined. pH adjustment of the metabolite solutions was performed to match the pH of the (fermented) cabbage homogenates applied onto the Caco-2 cells and obtain the conjugate base forms more likely to be present in pH levels more commonly found in the small intestine. The DMEM suspensions were sterilized using a 0.22 µm SFCA filter and stored at −80°C until use.

### Intestinal epithelial monolayer cytokine stimulation

To form a monolayer, Caco-2 cells between passages 35 and 36 were seeded onto polycarbonate membranes in Transwell inserts (0.33 cm^2^ surface area, 6.5 mm, 0.4 µm pore size, 24 wells; Corning, NY, USA) at a density of 1 × 10^4^ cells/cm^2^. After incubation for at least 21 days in complete DMEM containing 20% FBS, 1 mM sodium pyruvate, 0.1 mM nonessential amino acids, and 2 mM GlutaMax, the cells were fully differentiated and formed polarized monolayers with reduced permeability as quantified by trans-epithelial electrical resistance (TER). TER was measured after 15 min equilibration at room temperature with an epithelial voltohmmeter (World Precision Instruments, Sarasota, FL, USA) equipped with a STX-2 “chopstick” electrode. Caco-2 monolayers with an initial TER of more than 250 Ohm·cm^2^ were used for the subsequent experiments.

Prior to the application of the (fermented) cabbage homogenates or compounds, TER values were measured. Cabbage homogenates (10% vol/vol), selected metabolites (D-PLA, ILA, lactate, or the three combined), sterile water (10% vol/vol), or 0.0052% DMSO were applied to the apical side of the monolayers in supplemented DMEM. After 3 h incubation at 37°C, 100 ng/mL IFN-γ (R&D Systems, Minneapolis, MN, USA) was added to the basolateral compartment. Twenty-one hours later, the medium in the basolateral compartment was replaced with supplemented DMEM containing 10 ng/mL TNF-α (R&D Systems, Minneapolis, MN, USA). TER values of the monolayers were measured immediately prior to TNF-α exposure and 24, 32, and 48 h later.

### Paracellular permeability assay

Paracellular permeability was determined by the flux of fluorescein isothiocyanate-dextran (FITC-dextran; 4 kDa; Sigma-Aldrich, St. Louis, MO, USA) from the apical to basolateral side of differentiated Caco-2 monolayers as previously described (58). After 48 h stimulation with TNF-α, apical DMEM was replaced with FITC-dextran (2 mg/mL) in supplemented DMEM. After 30, 60, 90, and 120 min, FITC-dextran concentrations on the basolateral side were quantified by measuring the fluorescence intensity (485 nm excitation and 530 nm emission) in a microplate reader (Synergy-2, BioTek, Winooski, VT, USA). FITC-dextran levels were calculated using a standard curve of FITC-dextran in DMEM with the indicated supplements. The apparent permeability coefficient, P_app_ (cm/s), was determined using the following equation: P_app_ = [dQ/dt] × [1 / (A × C_0_)], where dQ/dt is the quantity of FITC-dextran transported per second (ng/s), A is the surface area of the filter (cm^2^), and C_0_ is the initial FITC-dextran concentration in DMEM on the apical side (ng/mL).

### ELISA quantification of IL-8

After 48 h stimulation with TNF-α, spent media collected from the basolateral compartment was used for the quantification of interleukin 8 (IL-8) via enzyme-linked immunosorbent assay (ELISA) (Life Technologies, Carlsbad, CA, USA) according to the manufacturer’s instructions.

### RNA extraction and reverse-transcription quantitative PCR (RT-qPCR)

After 48 h stimulation with TNF-α, total RNA from the Caco-2 monolayers was extracted using TRIzol (Invitrogen, Carlsbad, CA, USA) and purified using the TRIzol-chloroform protocol. RNA was quantified on the NanoDrop 2000c (Thermo Fisher, Waltham, MA, USA) and found to be intact (RIN 8.8–9.8) according to the Agilent 2100 Bioanalyzer (Agilent Technologies, Santa Clara, CA, USA). RNA was reverse transcribed to cDNA using the High-Capacity cDNA Reverse Transcription (RT) kit (Thermo Fisher Scientific, Waltham, MA, USA). RT-qPCR was performed using the Fast SYBR Green Master Mix (Thermo Fisher Scientific, Waltham, MA, USA) and 200 nM of the designated primers (Table 2) on a 7500 Fast Real-time PCR system (Applied Biosystems, Carlsbad, CA, USA). Amplification was initiated at 95°C for 20 s and was followed by 40 cycles of 95°C for 3 s and 60°C for 30 s. Primer specificity was assessed using melting curves following the amplification stage. All reactions were performed in duplicate, and reactions lacking RT for each sample were included to check for DNA contamination. Data were normalized using the 2^−ΔΔCt^ method (59) with *GAPDH* as the housekeeping gene and untreated cells as the reference condition.

**TABLE 2:** qRT-PCR primer DNA sequences

Gene	Primer sequence	Product (bp)	Efficiency (%)	Reference
*GAPDH*	F-5′-TCCCATCACCATCTTCCAG-3′	145	103.21	(60)
	R-5′-GATGACCCTTTTGGCTCC-3′			(61)
*TNFR2*	F-5′-TGGCATTTACACCCTACGC-3′	143	96.99	(58)
	R-5′-CACGGTGTCCGAGGTCTT-3′			
*MLCK*	F-5′-GCTTGGTCAGCCTGTTGTT-3′	135	103.09	(58)
	R-5′-CCTTCTTTGACCACAACTCG-3′			
*CLDN2*	F-5′-TGTGACATCTATAGCACCCTTC-3′	168	104.29	(58)
	R-5′-TACCGCCACTCTGTCTTTG-3′			

### Metabolomic analysis of cell-free (fermented) cabbage homogenates

Cabbage homogenates from the LSF and commercial ferments were prepared for gas chromatography-time of flight mass spectrometry (GC-TOF/MS) and reverse-phase liquid chromatography high-resolution tandem mass spectrometry (RP-LC-HRMS/MS) at the West Coast Metabolomics Center (WCMC), UC Davis (https://metabolomics.ucdavis.edu/) as described previously (62, 63). A complete list of detectable metabolites can be found at https://metabolomics.ucdavis.edu/metabolites (File S1).

For GC-TOF/MS, the samples were extracted using 3:3:2 acetonitrile (ACN): isopropyl alcohol (IPA): H_2_O (vol/vol/vol), dried, and then subjected to a two-step derivatization process using methoxyamine hydrochloride in pyridine and then a mixture of N-methyl-N-(trimethylsilyl)trifluoroacetamide (MSTFA) and fatty acid methyl esters (FAMEs) (62). The samples were analyzed with an Agilent 7890A gas chromatograph (Agilent, Böblingen, Germany) coupled to a Leco Pegasus IV TOF mass spectrometer (Leco, St. Joseph, MO, USA). Raw untargeted GC-TOF/MS data files were preprocessed by Leco ChromaTOF software and further analyzed using the BinBase pipeline (64) for identifying metabolites by retention index matching and mass spectra alignment. Peak height was reported for all untargeted analyses. Untargeted metabolite data were normalized using the mTIC method to reduce the impact of instrument sensitivity drift during the run by calculating the sum of all peak heights for all identified metabolites (excluding the unknowns) in each sample as described and performed previously (62, 65, 66). Additionally, the quantities of ILA, PLA, and GABA in (fermented) cabbage homogenates were quantified through targeted GC-TOF/MS with an external 6-point calibration curve. Overall, the GC-TOF/MS method was designed for untargeted and targeted primary metabolite detection and quantification, namely for carbohydrates and sugar phosphates, amino acids, hydroxyl acids, free fatty acids, purines, pyrimidines, and aromatics (62).

For RP-LC-HRMS/MS, the samples were extracted using 80:20 methanol: H_2_O, dried, and then suspended in a 75:25 ratio of H_2_O:ACN containing internal standards (1-cyclohexyl-dodecanoic acid urea [CUDA], D3-L-Carnitine, D4-Daidzein, D5-Hippuric acid-d5, D9-Reserpine, and Val-Tyr-Val) (63). RP-LC-HRMS/MS was performed using a Vanquish LC (Thermo Scientific, Waltham, MA, USA) coupled to a Waters Acquity Premier BEH C18 1.7 µm, 2.1 × 50 mm column, coupled to a Q-Exactive HF^+^ orbital ion trap mass spectrometer (Thermo Scientific) with positive and negative ion electrospray ionization (ESI) modes. MS-DIAL v. 4.92 (67) was used to process untargeted LC-MS/MS data with retention times and MS/MS spectra matching based on the MassBank of North America (68). Using this method, flavonoids, anthocyanins, polyphenols, alkaloids, coumarins, terpene- and polyphenol-glycosides, kaempferols, flavanones, cinnamates, and related compounds are detected.

Information on data acquisition, processing, and raw data normalization can be found in File S2. Untargeted GC-TOF/MS data includes the identified BinBase name, peak heights, retention index, m/z ratio, PubChem ID, KEGG ID, and InChl Key values. RP-LC-HRMS/MS data includes the identified metabolite name, MS-DIAL identifier, peak heights, m/z ratio, ESI mode, InChI key, and retention time.

### Statistical analysis

Data were analyzed using GraphPad Prism 10 software for Windows (GraphPad Software, Inc., La Jolla, CA, USA), unless stated otherwise. The cabbage fermentation (pH, bacterial cell counts), Caco-2 cell culture (TER, FITC-dextran, IL-8, RT-qPCR), and targeted GC-MS data were reported as mean ± standard deviation (SD) and analyzed by analysis of variance (ANOVA) with post hoc tests for comparing multiple treatments.

For untargeted metabolomics data analysis, peak height data sets were uploaded to MetaboAnalyst 6.0 (69, 70). Features (metabolites) with >50% missing values were removed, and the remaining missing values were replaced using the limit of detection (LoD) method (one-fifth of the minimum positive value of each variable) (71). Furthermore, features with >10% inter-quantile range (IQR) variance were filtered out. GC-TOF/MS peak heights were log_10_-transformed prior to analysis as performed previously (63). For the RP-LC-HRMS/MS data, peak heights were normalized to the sums of the peak heights of internal standards (File S3) added to each sample and then subjected to log_10_ transformation (63). Principal component analysis (PCA) was applied to evaluate the compositions of the six different (fermented) cabbage homogenates. To identify metabolites significantly elevated or reduced after fermentation, the relative peak heights of fermented homogenates were compared with the raw cabbage homogenates (D0) using the Wilcoxon rank-sum test (false discovery rate [FDR] of 0.05) (Files S4 and S5). Heatmaps of the top 35 significantly changed metabolites with the lowest *P* values were generated via hierarchical cluster analysis (Euclidean distance measure, Ward clustering method) and visualized after autoscaling to z-scores. Permutational multivariate anaysis of variance (PERMANOVA) and pairwise Adonis tests were performed using the LMSstat package (https://github.com/CHKim5/LMSstat/blob/master/README.md) and the pairwiseAdonis R package (72), respectively, in R 4.3.0 (73).

## RESULTS

### LP8826R dominates and modifies cabbage fermentations

**Fig 1:**
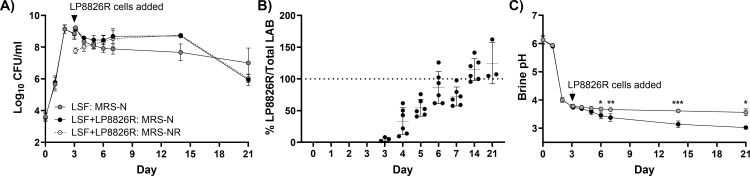
Bacterial numbers and brine pH in laboratory-scale ferments (LSF). (A) LAB were enumerated on MRS with natamycin (25 µg/mL) (MRS-N) and LP8826R was enumerated on MRS containing natamycin (25 µg/mL) and rifampicin (50 µg/mL) (MRS-NR). Rifampicin-resistant bacteria were not detected in the LSF lacking LP8826R additions (below 3.33 × 10^1^ CFU/mL limit). (B) The percentage of LP8826R to total LAB numbers over time and (C) brine pH. The mean ± SD is shown for six replicates. To determine differences in brine pH, two-way mixed effect ANOVA with Tukey’s multiple comparisons test was performed (*P < 0.05; **P < 0.01; ***P < 0.001).

According to culturable bacterial cell numbers on MRS agar, a nutritionally replete medium used to enrich LAB (74), there were 3.61 ± 0.25 log_10_ CFU/mL in the cabbage homogenates (D0) prior to the start of the laboratory scale fermentations (LSF). This number increased to 9.13 ± 0.26 log_10_ CFU/mL after 2 days of incubation at 22°C (Fig. 1A). Bacterial numbers then declined by approximately 10-fold to approximately 8.0 log_10_ CFU/mL by day 4 and were sustained at that level for at least 10 days. By day 21, average cell numbers declined by another 2-fold to 7.00 ± 0.94 log_10_ CFU/mL homogenate (Fig. 1A).

The addition of LP8826R to the LSF on day 3 elevated the total culturable LAB amounts in cabbage fermentations from 8.82 ± 0.25 log_10_ CFU/mL homogenate before inoculation to 9.14 ± 0.14 log_10_ CFU/mL approximately 3 h after inoculation (Fig. 1A). Over the subsequent 3 days, LP8826R numbers increased approximately 5-fold to 8.44 ± 0.28 log_10_ CFU/mL, as estimated on MRS with rifampicin (Fig. 1A). On that day, LP8826R constituted 86.50% ± 25.43% of total culturable LAB in the ferments (Fig. 1A and B). This percentage was sustained until the end of the study (Fig. 1B). Compared with the LSF, the total culturable LAB numbers in the LSF + LP8826R ferments were significantly higher on day 4 (*P* = 0.0320), day 5 (*P* = 0.0374), day 6 (*P* = 0.0298), and day 14 (*P* < 0.0001) (Fig. 1A). At day 21, total culturable LAB numbers between the LSF and LSF + LP8826R were not significantly different (*P* = 0.4015) (Fig. 1A).

In both LSF and LSF + LP8826R, reductions in pH coincided with the increase in bacterial numbers. The pH declined from pH 6.13 ± 0.13 at the start of the study (D0) to pH 4.00 ± 0.08 after the first 2 days of incubation (Fig. 1C). For the LSF, the brine pH continued to decrease, reaching pH of 3.61 ± 0.03 and 3.56 ± 0.12 on days 14 and 21, respectively (Fig. 1C). Brine pH values were significantly lower in the LSF + LP8826R compared with the LSF starting on day 6 of the fermentation (*P* < 0.05) (Fig. 1C). The significant reduction (*P* = 0.0206) in culturable LAB numbers between day 14 and day 21 observed in LSF + LP8826R may have been the result of the lower pH of those ferments.

Brine salinity was measured on days 14 and 21 of the LSF study. Brine salinity in the LSF did not change between day 14 (1.99% ± 0.16% [wt/vol]) and day 21 (1.95% ± 0.02% [wt/vol]). Similarly, brine salinity in the LSF + LP8826R were comparable between day 14 (2.05% ± 0.18% [wt/vol]) and day 21 (2.02% ± 0.05% [wt/vol]). There were also no significant differences between the LSF and LSF + LP8826R brines.

Commercial sauerkraut contained 5.11 ± 2.31 log_10_ CFU/mL homogenate according to growth on MRS agar (Table 1). These bacterial numbers were similar to levels found in the LSF on days 14 and 21 and in LSF + LP8826R on day 21, but less than those detected in LSF + LP8826R on day 14 (*P* < 0.0001). The pH of commercial sauerkraut homogenate (pH 3.34 ± 0.08) was significantly lower compared with LSF on days 14 and 21, but significantly higher compared with the LSF + LP8826R ferments (*P* ≤ 0.0092) (Fig. 1C; Table 1). The salinity of commercial sauerkraut brine was 2.67% ± 0.10% (wt/vol), significantly higher compared with the LSF and LSF + LP8826R (*P* < 0.002) (Table 1).

### Cabbage ferments prevent cytokine-induced intestinal barrier disruption

To establish a suitable range of fermented cabbage homogenate concentrations for assessment on Caco-2 cell monolayers, Caco-2 cell culture medium (DMEM) was adjusted to contain 1%, 10%, or 50% (vol/vol) of filtered, pH-adjusted (pH 7.4) homogenates from day 7 LSF and the commercial product. Compared with untreated controls, the apical application of either 1% or 10% (vol/vol) of the homogenates did not significantly change TER or permeability to FITC-dextran with sequential basolateral exposure to IFN-γ for 21 h and TNF-α for another 48 h (data not shown). However, TER was negatively affected when 50% (vol/vol) was applied, resulting in an average 10-fold reduction within the first 24 h of application (data not shown). This change was not a result of diluting the DMEM culture medium since applying an equal quantity of water (50% vol/vol) to the controls did not affect TER or FITC-dextran permeability (data not shown). To avoid the potential cytotoxic effects of the high (50% vol/vol) homogenate concentration, 10% vol/vol (fermented) cabbage homogenate was selected for use in subsequent experiments.

**Fig 2:**
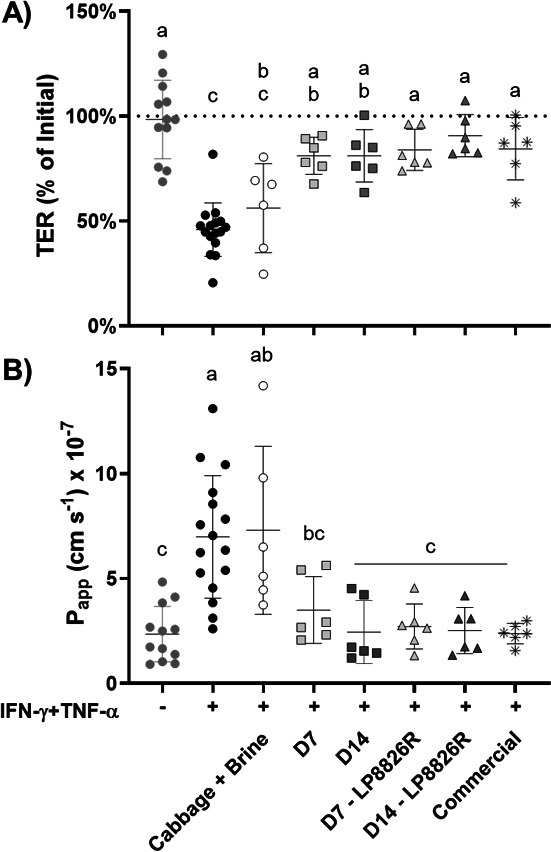
The effect of (fermented) cabbage homogenates on intestinal barrier permeability of cytokine-perturbed Caco-2 monolayers. (A) Trans-epithelial electrical resistance of Caco-2 monolayers 48 h after basolateral IFN-γ and TNF-α sequential additions. Values are normalized to TER immediately prior to when TNF-α was added. (B) Apparent permeability coefficient (P_app_) to FITC-dextran (4kD) of Caco-2 monolayers at the 48 h time point. (A) and (B) Cabbage + Brine constitute the raw cabbage homogenates from the start of the LSF (D0). D7 and D14 were collected on days 7 and 14, respectively, from LSF either with or without the LP8826R addition. Commercial denotes the commercial sauerkraut product. Replicates included Caco-2 monolayers not exposed to IFN-γ and TNF-α (*n* = 12), controls to which the cytokines were applied (*n* = 16), and those exposed to the cytokines and (fermented) cabbage (*n* = 6). The mean ± SD is shown for four independent experiments. The letters indicate significant differences based on one-way ANOVA with Tukey’s multiple comparisons test.

As found previously (58), basolateral incubation with IFN-γ for 21 h followed by TNF-α resulted in significant reductions of TER compared to untreated controls within 24 h after application that were sustained for the duration of the experiment (Fig. 2A; Fig. S1; *P* < 0.0001). IFN-γ and TNF-α induced reductions in TER were not found for monolayers incubated with either the commercial product or the LSF, irrespective of time of sampling or LP8826R, and instead, TER levels were similar to the untreated controls (*P ≥* 0.2008) (Fig. 2A). TER of Caco-2 monolayers exposed to the commercial product or LSF or LSF + LP8826R were also significantly greater than the IFN-γ and TNF-α-treated controls, with exception of the LSF collected at day 14 and measured 24 h after TNF-α addition (Fig. S1). By comparison, raw cabbage homogenates (D0) had reduced TER compared with the untreated controls at all time points (Fig. 2A; Fig. S1) and were not significantly different from the cytokine-treated controls starting 32 h after TNF-α application (Fig. S1). To investigate the effects of cabbage and saline on TER, Caco-2 cell monolayers were either incubated with a raw cabbage homogenate collected after homogenization in water (instead of saline) or DMEM adjusted to contain 10% (vol/vol) of a 2.91% (wt/vol) NaCl brine solution prepared to match the salinity of the commercial product. The former preparation did not result in barrier protection, as shown by the significantly lower TER (*P* ≤ 0.03) compared with untreated controls (Fig. S2A). The addition of NaCl to DMEM resulted in no significant differences in TER compared with either the IFN-γ- and TNF-α-treated or -untreated controls (Fig. S2A).

Assessments of paracellular permeability to FITC-dextran were performed 48 h after TNF-α addition. Significantly higher quantities of FITC-dextran were detected in the basolateral compartment of Caco-2 cell monolayer controls treated with IFN-γ and TNF-α compared with those not exposed to the cytokines (Fig. 2B; P_app_ coefficient; *P* < 0.0001), confirming that the cytokines disrupted barrier integrity. Notably, Caco-2 monolayers to which either LSF, LSF + LP8826R, or the commercial product homogenate were added had significantly lower permeability to FITC-dextran than the cytokine-treated controls (*P* < 0.0292) and were equal to the untreated controls (Fig. 2B). In contrast, the raw cabbage homogenates (D0) conferred no protection against IFN-γ and TNF-α induced permeability (*P* < 0.0007) (Fig. 2B). Similarly, no protection was found for cytokine-treated monolayers exposed to raw cabbage homogenized in water instead of saline (Fig. S2B). Finally, Caco-2 cell monolayers exposed to NaCl (0.291% [wt/vol]) had intermediate levels of FITC-dextran translocation that were not significantly different from either the untreated and cytokine-treated monolayers (Fig. S2B). Together, these results indicate that fermented cabbage, but not raw cabbage or brine, had the capacity to protect against inflammation-induced intestinal barrier damage.

### Fermented cabbage variably altered IL-8 secretion and expression of barrier-modulatory genes

**Fig 3:**
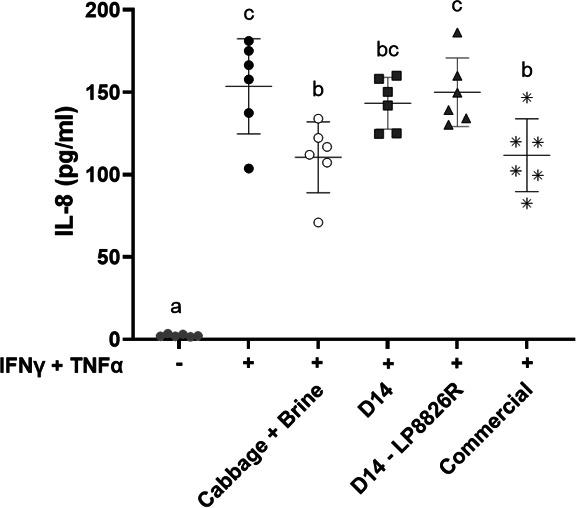
IL-8 production by Caco-2 monolayers after sequential exposure to IFN-γ and TNF-α. DMEM was collected from the basolateral side of Caco-2 monolayers 48 h after sequential application of IFN-γ and TNF-α. Cabbage + Brine constitute the raw cabbage homogenates from the start of the LSF (D0). D14 and D14 LP8826R were collected on day 14 from LSF either without or with LP8826R addition. Commercial denotes the commercial sauerkraut product. The mean ± SD is shown for selected basolateral spent media from four independent experiments (*n* = 6). Six spent media replicates were randomly selected from monolayers not exposed to cytokines (*n* = 12) and controls exposed to cytokines (*n* = 16). Letters indicate significant differences based on one-way ANOVA with Tukey’s multiple comparisons test.

Concentrations of IL-8, a pro-inflammatory chemokine and indicator of inflammatory responses in epithelial cells (75), were significantly higher in the basolateral medium of Caco-2 cell monolayers exposed to IFN-γ and TNF-α compared with the untreated controls (*P* < 0.0001) (Fig. 3). Similarly high levels of IL-8 were found for the LSF with and without LP8826R (Fig. 3). Quantities of IL-8 were significantly reduced for monolayers exposed to the commercial product compared to controls exposed to IFN-γ and TNF-α (*P* = 0.0138), albeit the quantities were still higher than the controls not exposed to the cytokines (Fig. 3). Interestingly, the levels of IL-8 were similarly reduced after exposure to the raw cabbage and brine mixture (D0) (Fig. 3).

RT-qPCR was performed to quantify the expression of the TNF receptor (*TNFR2*), encoding for a cell surface receptor mediating the effects of tumor necrosis factor (TNF) family, myosin light chain kinase (*MLCK*), involved in regulating intestinal barrier function through the phosphorylation of myosin light chain (MLC), and *CLDN2*, encoding for the pore-associated tight junction protein Claudin-2. Transcript levels of *TNFR2* and *CLDN2* were approximately 2-fold higher with IFN-γ and TNF-α exposure, and *MLCK* levels were slightly reduced compared with the untreated controls (Fig. S3). The application of fermented cabbage homogenates did not significantly alter the expression of *TNFR2*, *MLCK*, or *CLDN2*, with the exception of the LSF + LP8826R ferments, which had similar *MLCK* expression levels as found for the untreated controls (Fig. S3).

### LSF and commercial cabbage ferments and raw cabbage have distinct metabolomes

**Fig 4:**
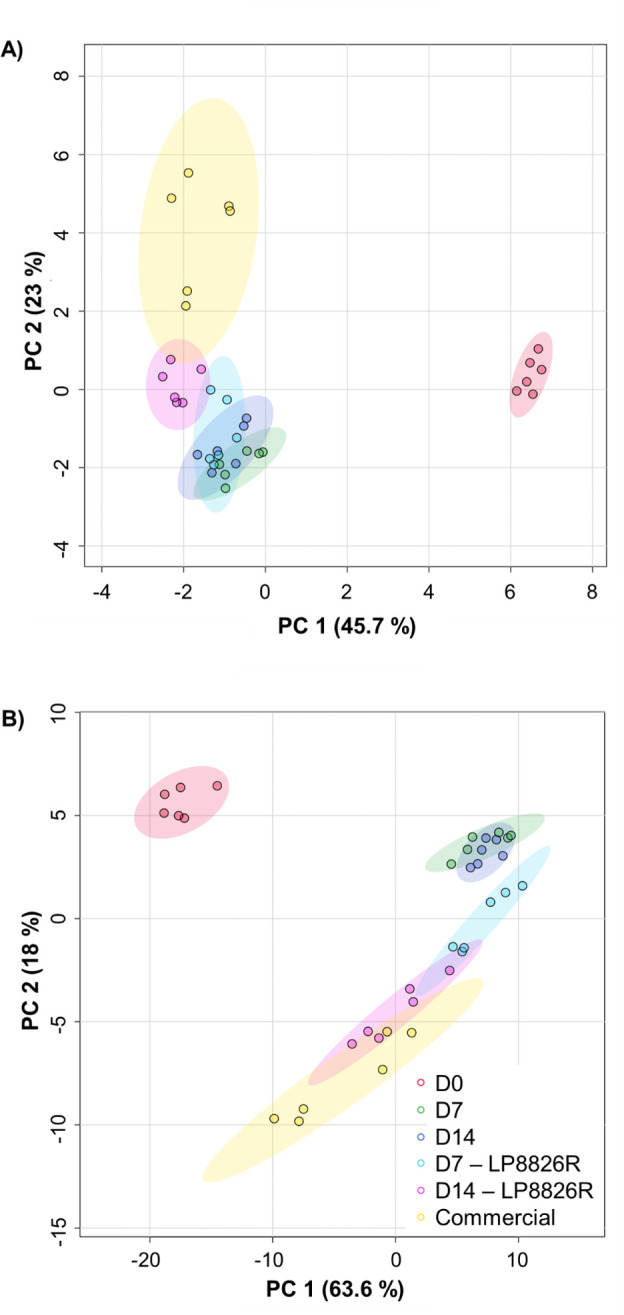
Principal component analysis (PCA) of metabolites identified by (A) GC-TOF/MS (147 metabolites) and (B) RP-LC-HRMS/MS (333 metabolites). Six replicates were tested for homogenates of cabbage and brine from day 0 (D0), day 7 laboratory scale ferments (LSFs) without (D7) and with LP8826R (D7 - LP8826R), day 14 LSFs without (D14) and with LP8826R (D14 - LP8826R), and commercial ferments.

GC-TOF/MS detected 583 metabolites in (fermented) cabbage homogenates, among which 147 were identified (File S4). Principal component analysis (PCA) showed that the metabolome profiles of the raw and fermented homogenates were significantly different (PERMANOVA *P* = 0.001, R_2_ = 0.95305) (Fig. 4A). Metabolites in the raw cabbage (D0) clustered separately from the fermented cabbage homogenates (*p_adj_* ≤0.06, pairwise Adonis test) (Fig. 4A). The commercially prepared ferments were also distinct from the LSF (*p_adj_* = 0.045, pairwise Adonis test) (Fig. 4A). Among the LSF, those with LP8826R were similar to the LSF prepared without this exogenous strain (*p_adj_* = 0.210) on day 7 of the study but then changed and were significantly different from the LSF on day 14 (*p_adj_* = 0.045) and resembled the commercial sauerkraut (*p_adj_* = 0.075, pairwise Adonis test) (Fig. 4A).

**Fig 5:**
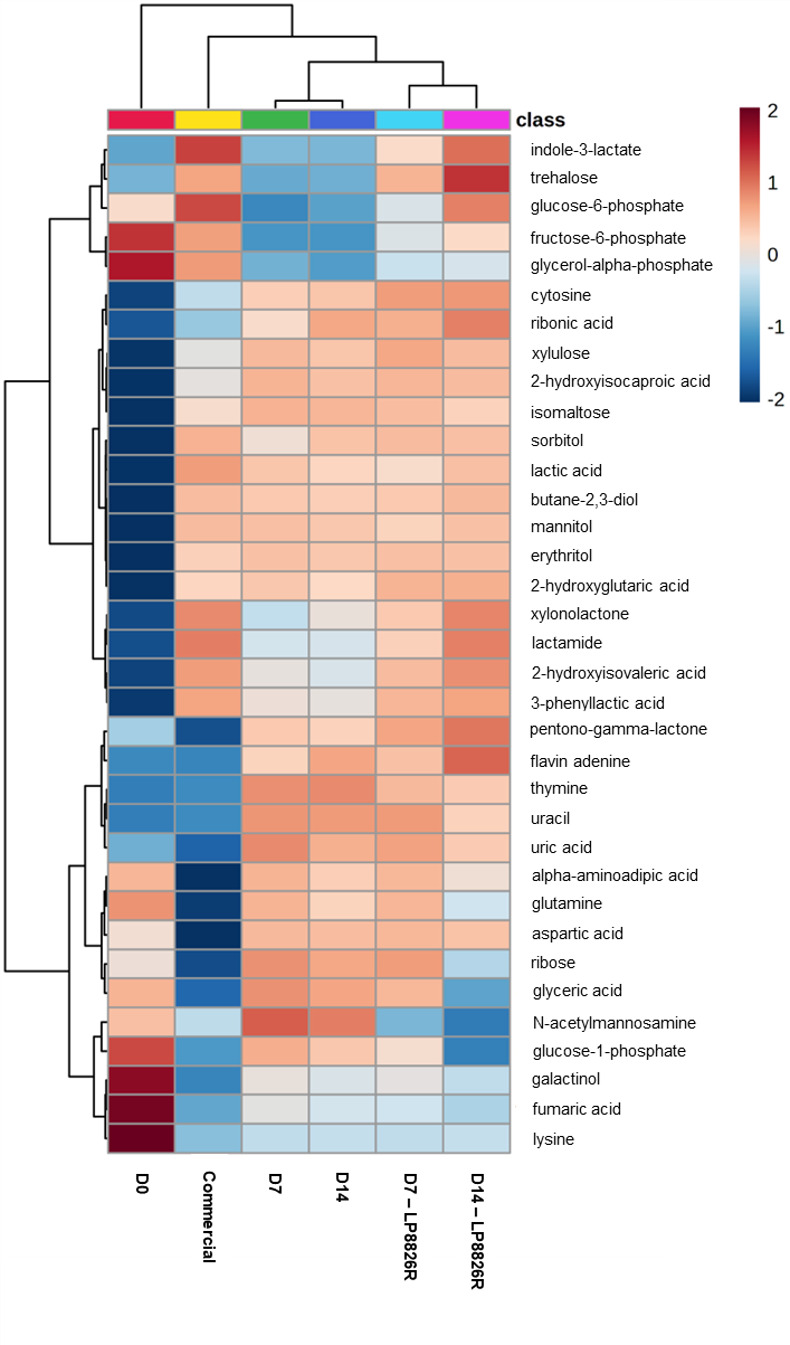
Heatmap of the relative peak heights of the top 35 metabolites detected by GC-TOF/MS that are significantly different between (fermented) cabbage homogenates. Metabolites were detected from homogenates (*n* = 6) of cabbage and brine from day 0 (D0), day 7 laboratory scale ferments (LSFs) without (D7) and with LP8826R (D7 - LP8826R), day 14 LSFs without (D14) and with LP8826R (D14 - LP8826R), and commercial ferments. Visualization of the compounds was created using log_10_ normalized data. The blue color represents lower relative peak heights while the red color represents higher relative peak heights. Metabolite features were standardized through autoscaling to z-scores. Similarity between data points was assessed using the Euclidean distance measure and then hierarchically clustered using Ward’s method. The top 35 features with the lowest *P* values were determined by one-way ANOVA with Tukey’s multiple comparisons test.

A total of 59 metabolites identified using GC-TOF/MS were significantly increased in at least one group of ferments and 20 of these metabolites were significantly increased across all ferments compared with the raw cabbage homogenates (Wilcoxon rank-sum test, *P* < 0.05 and fold change ≥2) (File S4). These compounds mainly encompassed end-products of fermentation metabolism (e.g., lactate and butane-2,3-diol), sugar and sugar alcohols (e.g., sorbitol, mannitol, trehalose, and isomaltose), several amino acids (e.g., phenylalanine, tyrosine, and leucine) and derivatives (e.g., PLA and ILA), and nucleobases (e.g., uracil and cytosine). Conversely, proportions of 49 metabolites were significantly reduced in at least one group of ferments and 15 of them were reduced in all ferments compared with the raw cabbage homogenates, including core metabolism intermediates (e.g., pyruvic acid, citric acid, and alpha-ketoglutarate), sugars and sugar acids (e.g., glucose, fructose, mannose, raffinose, and sucrose), and other amino acids (e.g., lysine, asparagine, and glutamic acid) (Wilcoxon rank-sum test, *P* < 0.05; Fig. 5; Fig. S4; File S4). Based on correlation biplots between metabolites and treatment groups, raw cabbage homogenates (D0) were positively correlated with the levels of sucrose, raffinose, and to a lesser extent, mannose and fructose (Fig. S5A). Cabbage ferments were mainly grouped together according to the proportions of fermentation end-products (e.g., sorbitol, lactic acid, mannitol, and butane-2,3-diol), PLA, ILA, and trehalose (Fig. 5; Fig. S4 and S5A). Commercial sauerkraut homogenates were further distinguished from LSF homogenates by their higher levels of putrescine and glucose-6-phosphate (Fig. S4 and S5A).

RP-LC-HRMS/MS detected 6,778 metabolites in (fermented) cabbage homogenates, among which 333 metabolites were identified. As found with GC-TOF/MS, the composition and proportions (peak heights) of those identified were significantly different between the (fermented) cabbage homogenate types (PERMANOVA, *P* = 0.001, R_2_ = 0.80297) (Fig. 4B). Metabolomes of the raw cabbage and brine (D0) homogenates clustered separately from fermented cabbage homogenates (*p_adj_* ≤0.06, pairwise Adonis test) (Fig. 4B). The composition and abundance of these metabolites in the LSF were similar across time points. The commercial sauerkraut was distinct from the LSF (*p_adj_* <0.05, pairwise Adonis test), except for the ferments with added LP8826R at day 14 (*p_adj_* = 0.075, pairwise Adonis test) (Fig. 4B). Hence, as found for GC-TOF/MS, the RP-LC-HRMS/MS metabolite profiles show that fermentation changes the metabolomes and also that the addition of LP8826R results in a metabolome which better resembles commercial sauerkraut (Fig. 4B).

Comparing the two metabolomics methods, 41 metabolites were identified by both GC-TOF/MS and RP-LC-HRMS/MS (e.g., some amino acids, sugars, and amines), covering approximately one-third (41 of 147 metabolites; 28%) of the GC-TOF/MS identified compounds and approximately 12% (41 of 333 metabolites) of RP-LC-HRMS/MS identified compounds. The other remaining metabolites identified only with RP-LC-HRMS/MS (292 of 333 metabolites; ~88%) mainly consisted of di-peptides, fatty acids, organo-sulfur, and poly-cyclic (e.g., epigallocatechin) compounds.

**Fig 6:**
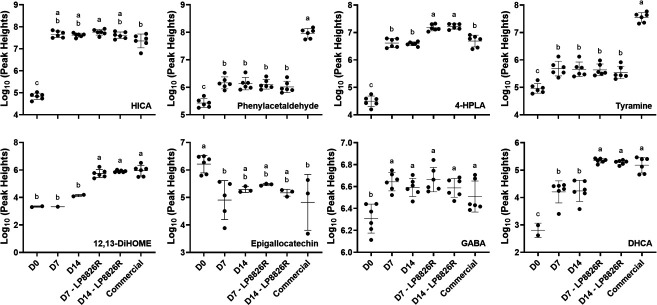
Representative metabolites detected by RP-LC-HRMS/MS. D0: cabbage + brine homogenates from the start of the LSF. D7 and D14 were collected on days 7 and 14, respectively, from LSF either with or without the LP8826R addition. Commercial denotes the commercial sauerkraut product.. Mean values not sharing letters are significantly different based on one-way ANOVA with Tukey’s multiple comparisons test. Mean ± SD for *n* = 6 replicates. Missing data points indicate that the metabolite is not detected. HICA: 2-hydroxyisocaproic acid. 4-HPLA: 4-hydroxyphenyllactic acid. 12,13-DiHOME: 12,13-dihydroxy-9Z-octadecenoic acid. GABA: γ-aminobutyric acid. DHCA: 3,4-hydroxylhydrocinnamic acid.

In total, 254 compounds detected and identified by RP-LC-HRMS/MS were significantly increased in at least one of the fermented cabbage homogenates and 110 metabolites were significantly elevated across all ferments compared to the raw cabbage homogenates (D0) (Wilcoxon rank-sum test, *P* < 0.05 and fold change ≥2) (File S5). Among these compounds, 27 metabolites were also detected by GC-TOF/MS and included certain amino acids (e.g., tryptophan and tyrosine) and derivatives (e.g., PLA, ILA, 2-hydroxyisocaproic acid [HICA]) and sugar alcohols (e.g., mannitol and sorbitol). The other compounds significantly enriched in at least one of the fermented cabbage homogenates were only detected by RP-LC-HRMS/MS and mainly consisted of small di- and tri-peptides (144 of 254 compounds; 56.7%) (e.g., phe-phe), phenolic and heterocyclic compounds (17 compounds; e.g., gentisic acid, 3,4-dihydroxyhydrocinnamic acid [DHCA]), other amino acid derivatives (38 compounds; e.g., 4-hydroxyphenyllactic acid [4-HPLA]), fatty acids (20 compounds; e.g., mevalonic acid), and vitamins and derivatives (four compounds; e.g., vitamin C, vitamin B5) (Fig. 6; Fig. S6; File S5). On the other hand, the relative abundances of 49 compounds were significantly lower in at least one of the ferments and nine metabolites were decreased in all ferments relative to the raw cabbage homogenates. Among these 49 compounds, nine metabolites were also detected by GC-TOF/MS and included sugars (e.g., fructose, sucrose), pyrimidines and purines (e.g., uracil, uridine), and organic acids (citric acid, gluconic acid). The remaining 40 compounds only detected by RP-LC-HRMS/MS encompassed other sugars (e.g., turanose) and other fatty acids (e.g., (9Z)−5,8,11-trihydroxyoctadec-9-enoic acid) (Wilcoxon rank-sum test, *P* < 0.05; File S5).

Based on correlation biplots between metabolites and treatment groups, the raw cabbage homogenates were distinguished from ferments by the lower relative abundance of epigallocatechin (Fig. S5B; Fig. 6). Commercial sauerkraut and LSF + LP8826R homogenates were further differentiated from other LSF homogenates by their higher relative levels of 12,13-dihydroxy-9Z-octadecenoic acid (12,13-DiHOME), DHCA, ILA, and PLA (Fig. 6; Fig. S5B and S6). Compared with other cabbage ferments and raw cabbage homogenate, commercial ferments had higher levels of tyramine and phenylacetaldehyde (Fig. 6; Fig. S6).

**Fig 7:**
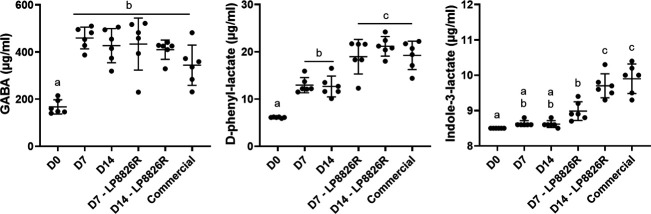
Quantities of ILA, D-PLA, and GABA in (fermented) cabbage. Metabolites were quantified in homogenates of cabbage and brine from day 0 (D0), day 7 laboratory scale ferments (LSFs) without (D7) and with LP8826R (D7 - LP8826R), day 14 LSFs without (D14) and with LP8826R (D14 - LP8826R), and commercial ferments. Mean ± SD shown for *n* = 6 replicates. Mean values not sharing letters are significantly different based on one-way ANOVA with Tukey’s multiple comparisons test.

To verify the findings of untargeted metabolomics and gain estimates of total quantities of known bioactive compounds in the ferments, targeted GC-TOF/MS was applied to measure quantities of ILA, D-PLA, and GABA. ILA, D-PLA, and GABA were selected due to their known production by LABs, such as *L. plantarum* (76–78) and reported presence in fermented cabbage (31, 79–81). The findings of the targeted analyses (Fig. 7) were similar to those reached by untargeted GC-TOF/MS (ILA, D-PLA) and RP-LC-HRMS/MS (ILA, D-PLA, and GABA) (Fig. 5 and 6). Of those three compounds, GABA was detected in the highest concentrations in the raw cabbage homogenate (D0, 167.1 ± 30.4 µg/mL). GABA levels more than doubled after fermentation (*P* < 0.0001). There were no differences in GABA quantities between the LSF and commercial ferments (Fig. 6 and 7). Fermentation also resulted in significant increases in PLA in all ferments compared with the controls (D0) (Fig. 7). PLA levels increased from approximately 6 µg/mL (~36 µM) in raw cabbage and brine (D0) to the highest concentrations of approximately 20 µg/mL (120 µM) in the LSF + LP8826R and the commercial ferments. ILA levels were significantly increased in the commercial sauerkraut and LSF + LP8826R, but not LSF (Fig. 7). ILA concentrations increased from approximately 8.5 µg/mL (~41 µM) in cabbage and brine to the highest concentrations of approximately 10 µg/mL (~50 µM) in the day 14 LSF + LP8826R homogenate and the commercial ferments.

### D-PLA, ILA, and lactate protect Caco-2 cell monolayers from cytokine-induced damage

**Fig 8:**
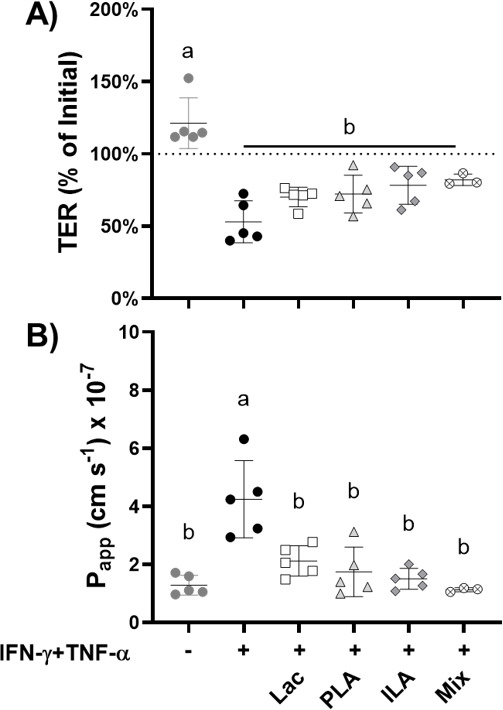
Effect of lactate, PLA, and ILA separately and combined (mix) on intestinal barrier permeability of cytokine-perturbed Caco-2 monolayers. (A) Trans-epithelial electrical resistance (TER) of Caco-2 monolayers 48 h after IFN-γ and TNF-α sequential additions. TER values were normalized to TER measured immediately prior to TNF-α addition. (B) Apparent permeability coefficient (P_app_) to FITC-dextran (4kD) of Caco-2 monolayers measured at the 48 h time point. Replicates included Caco-2 monolayers not exposed to IFN-γ and TNF-α (*n* = 5), controls to which the cytokines were applied (*n* = 5), and those exposed to the cytokines and either 50 mM (4504 µg/mL) lactate (Lac) (*n* = 5), 60 µM (10 µg/mL) D-phenyl-lactate (PLA) (*n* = 5), 25 µM (5 µg/mL) indole-3-lactate (ILA) (*n* = 5), or a mixture of the three metabolites (Mix) (*n* = 3). Mean ± SD. Mean values not sharing any letter are significantly different based on (TER) two-way or (FITC-dextran) one-way ANOVA with Tukey’s multiple comparisons test.

We next tested whether individual metabolites enriched in fermented cabbage are sufficient to protect Caco-2 cell monolayers against IFN-γ and TNF-α induced damage. Lactate, D-PLA, and ILA were selected because of their association with exerting beneficial impacts on intestinal barrier function, immune development, and lipid metabolism (19, 30, 32, 34, 82). GABA was not included because it may require signaling from enteric neuronal or endocrine-like cells, which are not a part of our Caco-2 cell model. The monolayers were exposed to apical applications of pH-adjusted (pH 7.4) and filter-sterilized 50 mM lactate (approximately 4.5 mg/mL), 60 µM D-PLA (approximately 10 µg/mL), or 25 µM ILA (approximately 5 µg/mL), and a mixture of all three compounds at the specified concentrations (Fig. 8). The compounds were applied in concentrations comparable with those detected for PLA and ILA in fermented cabbage based on targeted GC-TOF/MS analysis, taking into account the homogenate dilutions applied onto the Caco-2 cell monolayers (Fig. 7) and lactate concentrations in fermented cabbage found in prior studies (81, 83).

**Fig 9:**
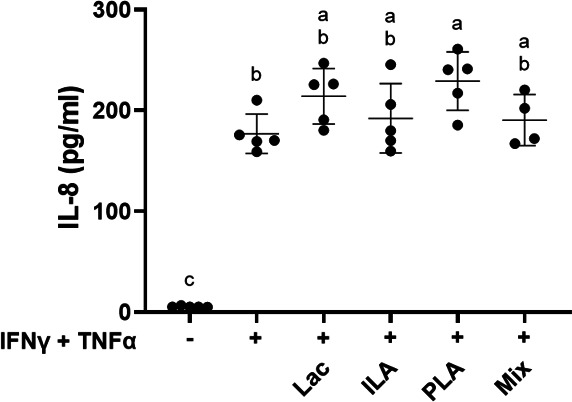
IL-8 production by Caco-2 monolayers after sequential exposure to IFN-γ and TNF-α. DMEM was collected from the basolateral side of Caco-2 monolayers 48 h after sequential application of IFN-γ and TNF-α. Replicates included Caco-2 monolayers not exposed to IFN-γ and TNF-α (*n* = 5), controls to which the cytokines were applied (*n* = 5), and those exposed to the cytokines and either 50 mM (4504 µg/mL) lactate (Lac) (*n* = 5), 60 µM (10 µg/mL) D-phenyl-lactate (PLA) (*n* = 5), 25 µM (5 µg/mL) indole-3-lactate (ILA) (*n* = 5), or a mixture of the three metabolites (Mix) (*n* = 4). The mean ± SD is shown. Letters indicate significant differences based on one-way ANOVA with Tukey’s multiple comparisons test.

Apical applications of D-PLA, ILA, and lactate, individually and combined, did not prevent cytokine-induced reductions in TER when measured 24 and 48 h after TNF-α addition (Fig. 8A; Fig. S7). A modest benefit was found at the 32 h time point such that the TER was significantly higher related to the cytokine-treated controls (*P* < 0.01, two-way ANOVA with Tukey’s multiple comparisons test) (Fig. S7). Despite the minimal effect on TER, D-PLA, ILA, and lactate, both individually and combined, attenuated the increase in paracellular permeability such that the levels of FITC-dextran were significantly reduced (*P* ≤ 0.002, one-way ANOVA with Tukey’s multiple comparisons test) compared with the cytokine-treated controls, and permeability was similar to the untreated controls (*P* > 0.4997) (Fig. 8B). As found for the fermented cabbage, IL-8 levels were significantly elevated for cytokine-challenged Caco-2 cells incubated with the three metabolites, separately and combined, compared with untreated controls (*P* < 0.0001, one-way ANOVA with Tukey’s multiple comparisons test) (Fig. 9). Cytokine-challenged monolayers incubated with D-PLA had significantly higher IL-8 production compared with the cytokine controls (*P* = 0.0343) (Fig. 9). These results show the capacity of the individual metabolites to protect Caco-2 cell monolayers and that combining the three of them resulted in a more consistent, although not greater effect, suggesting possible contributions of multiple beneficial metabolites to intestinal barrier protection.

## DISCUSSION

To investigate whether fermented cabbage has the potential to support intestinal epithelial barrier function, we examined both laboratory-scale and commercial cabbage ferments in a polarized Caco-2 cell model. This showed that soluble filtrates of fermented cabbage homogenates, and not comparable preparations of raw cabbage or brine, protected against the cytokine (IFN-γ and TNF-α) induced barrier disruption. Differences in intestinal barrier protection may be explained by fermentation-mediated changes to the cabbage metabolome and enrichment in gut barrier-protective compounds. To this regard, the application of fermentation-enriched metabolites, D-PLA, ILA, and lactate protected against IFN-γ and TNF-α induced increases to paracellular permeability measured with FITC-dextran, although they were not effective according to TER, a metric which assesses the ionic conductance of the intestinal epithelial monolayer. The findings suggest that the intestinal epithelial barrier protective bioactivity of fermented cabbage likely involves the synergistic effect of multiple metabolites produced or modified by microorganisms during fermentation. These compounds were present across the ferments and were sufficient to prevent barrier disruption, although differences between the fermented cabbage metabolomes were found. Our results indicate that fermented cabbage contains a core metabolome that protects the intestine from inflammatory damage.

Application of a sterile filtrate of soluble fermented cabbage metabolites alleviated the increased Caco-2 monolayer permeability induced by sequential exposure to IFN-γ and TNF-α. Fermented cabbage homogenates prevented cytokine-induced reductions in TER by approximately 40% by 48 h after basolateral TNF-α application and prevented cytokine-induced paracellular FITC-dextran translocation. A similar trend was also observed for filtrates of milk fermented by *Lacticaseibacillus paracasei* BL23, which prevented the TER reductions and lowered permeability to FITC-dextran compared with cytokine-challenged monolayers (58). The capacity to prevent inflammation-induced disruption to intestinal barrier integrity in Caco-2 monolayers has also been demonstrated for probiotics (46, 84) and LAB isolated from fermented cabbage (85, 86), food extracts (e.g., cocoa polyphenols, wine phenolic extracts) (87), fermented food (e.g., yogurt [88]), and specific bioactive compounds (e.g., short chain fatty acids [89]). Although findings from these studies are insufficient to confirm that those dietary components are able to prevent acute or chronic impacts of intestinal inflammation, the current, but notably limited, findings from human studies on lacto-fermented fruits and vegetables also reported anti-inflammatory effects (3).

Notably, despite the capacity of fermented cabbage homogenates to maintain barrier function, basolateral IL-8 levels were only reduced for commercial ferments, and surprisingly for the raw cabbage controls (D0). Fermented cabbage also had no detectable effect on *TNFR2*, *MLCK*, and *CLDN2* transcript levels. These results indicate that the influence of fermented cabbage on barrier function were distinct from the IFN-γ- and TNF-α-induced Caco-2 inflammatory response. Aryl hydrocarbon receptor (AHR) transcription factor activation, such as by LAB fermentation-derived ILA (36), and interaction with RelB, one of the five transcription factors in the NF-ĸB family, may contribute to increased IL-8 levels (90), whereas NF-ĸB induced MLCK elevation may be inhibited at a different point of the signaling pathway through the activation of peroxisome proliferator-activated receptor gamma (PPAR-γ) (91, 92) by LAB fermentation-derived ligands like PLA (93). Thus, the diversity of fermentation-derived metabolites present in the cabbage ferments may have contributed to this separation between TNF-α induced NF-ĸB activation of the inflammatory response (e.g., IL-8 production) and maintenance of intestinal barrier permeability. Future studies should focus on activation status levels of NF-ĸB subunits and other potential signaling factors (e.g., AHR, PPAR-γ) over time (94), rather than at a single time point, to uncover the underlying mechanisms responsible for modulation of epithelial barrier permeability by fermented cabbage metabolites.

Untargeted metabolome analysis was used to identify the specific compounds responsible for the observed barrier-protective effects of the fermented cabbage homogenates. GC-TOF/MS and RP-LC-HRMS/MS analyses showed that compared with the raw cabbage (D0), fermented cabbage homogenates were significantly different in ways predictable based on other LAB fermentations (e.g., higher and lower levels of lactic acid and sugars, respectively), such as observed for artisanal sauerkrauts (81) and home fermented (80), and laboratory scale Chinese northeast sauerkrauts (83, 95). Other changes to the fermented cabbage metabolomes remain to be further investigated (e.g., increases in fatty acids such as mevalonic acid, 12,13-dihydroxy-9Z-octadecenoic acid [12,13-DiHOME], and cis-pinonic acid).

In addition to metabolites that were consistently modified across more than one group of ferments, putrescine and tyramine biogenic amines were significantly increased in the commercial product. The higher levels of putrescine may have been at least partially due to the storage of the product at 4°C, as found previously (96). Additionally, PCA biplot analysis showed that the metabolites in the LSF + LP8826R ferments collected on day 14 were associated with higher levels of lactic acid, butane-2,3-diol, sorbitol, and mannitol compared with the other LSF, indicating that the addition of the probiotic caused significant changes to the microbial communities and their metabolic activities.

Interestingly, LP8826R altered the LSF cabbage metabolomes and Caco-2 responses such that those ferments better resembled the commercial product. LP8826R dominated the total culturable LAB population three days after addition, leading to elevated total LAB numbers and lower brine pH compared to LSF without LP8826R. A similar result was found after the addition of a *L. plantarum* strain to Chinese northeastern sauerkraut fermentations (83). These findings show the potential application of adding probiotics or other starter cultures in vegetable fermentations for accelerating the fermentation process and for selective enrichment of desired compounds in the final product.

Targeted GC-TOF/MS quantification confirmed the presence of GABA, PLA, and ILA in the (fermented) cabbage homogenates. Increases in GABA were expected because GABA is a product of LAB glutamine deamidation, a pathway that prevents reductions in intracellular pH in acidic environments (97). GABA levels in the LSF and commercial ferments were approximately 400 µg/mL, quantities higher than detected in previous studies on fermented cabbage (80, 81). PLA and ILA were similar to (PLA) or higher (ILA) than levels in vegetable ferments (e.g., sauerkraut, fermented carrots, and kimchi) measured previously (31, 98). However, there was variation in PLA and ILA over time and between the laboratory-scale and commercial ferments, indicating that the quantities of these compounds in the ferments were affected by strain- and microbial-community-specific differences.

Treatment of the Caco-2 monolayers with lactate, ILA, and PLA metabolites individually and as a mixture protected against cytokine-induced increase in paracellular permeability, as measured by FITC-dextran translocation, but neither sustained protection against TER reductions nor led to reductions in IL-8 production. Although the quantities of PLA and ILA in the ferments were relatively low, prior studies suggest they were higher than needed to induce a response in human cells (30). Similarly, lactate levels were greater than needed for activation of the human HCA1/GPR81 receptor (16, 82). Thus, at least for protection against IFN-γ and TNF-α as measured in the Caco-2 monolayer model here, the complexity of metabolites in the whole food (filtered homogenate) appears to be more protective compared to the individual metabolites. With this noted, other metabolites have yet to be tested, such as phenolic compounds (e.g., DHCA, gentisic acid, cynarin, 3-coumaric acid) recognized for their antioxidant capacity, contributing to oxidative stress reduction properties of fermented plant-based foods (99). GABA may also be of interest because of its neuroprotective (100) and anti-hypertensive (101) properties. These compounds were not tested here because they comprise a complex set of compounds that should be more systematically examined (phenolic compounds) or should be studied in culture systems with more diverse cell types (GABA).

Remarkably, all cabbage ferments exhibited intestinal barrier protective effects, despite the differences in their metabolomes. Other fermented cabbage (sauerkraut) products should be tested to determine whether barrier protection is a robust trait and to identify the presence of a core metabolome that confers bioactivity. These products should encompass ferments made commercially as well as LSF ferments with different ingredients, strain inoculants, and fermentation and post-fermentation storage times. Additional studies are also needed to explore the impacts of homogenization methods on the soluble fermented cabbage metabolome. This work can incorporate measurements of the extent to which cabbage is metabolized in the human digestive tract (102). Furthermore, assessments of the effects of cabbage ferments and their metabolites should be performed with other intestinal *in vitro* models (e.g., non-transformed intestinal epithelial cells and intestinal organoids).

In conclusion, based on our findings, forthcoming studies should elucidate the specific metabolites and signaling pathways via which fermented cabbage may improve intestinal epithelial barrier function. Verifying the potential health-promoting properties of fermented fruit and vegetable foods can also support the identification of starter culture strains that produce desired bioactive compounds. Ultimately, human studies are required to determine recommended intakes of fermented cabbage that will yield physiologically relevant benefits to gut health.

## Data Availability

The GC-TOF/MS and RP-LC-HRMS/MS peak height data sets used in the untargeted metabolomic analysis are available in the Harvard Dataverse repository at https://doi.org/10.7910/DVN/EM6HXV.

## SUPPLEMENTAL MATERIAL

File S1 aem.02234-24-s0001.pdf Metabolites available for detection.

File S2 aem.02234-24-s0002.pdf GC methods.

File S3 aem.02234-24-s0003.xlsx Peak heights of internal standards used for untargeted LC-RP-HRMS/MS.

File S4 aem.02234-24-s0004.xlsx GC-TOF/MS metabolites significantly changed between ferments and raw cabbage.

File S5 aem.02234-24-s0005.xlsx LC-RP-HRMS/MS metabolites significantly changed in ferments compared to raw cabbage.

Supplemental figures aem.02234-24-s0006.docx Figures S1 to S7 and legends for Files S1 to S5.

ASM does not own the copyrights to Supplemental Material that may be linked to, or accessed through, an article. The authors have granted ASM a non-exclusive, world-wide license to publish the Supplemental Material files. Please contact the corresponding author directly for reuse.
