# Supplementary material for: The fermented cabbage metabolome and its protection against cytokine-induced intestinal barrier disruption of Caco-2 monolayers
Source: Appl Environ Microbiol. 2025 Apr 7;91(5):e02234-24. doi: 10.1128/aem.02234-24 (PMC12093966; doi:10.1128/aem.02234-24)
Supplement: File S1 — Metabolites available for detection. [file aem.02234-24-s0001.pdf]

Metabolites

Metabolites We Can Detect

If you are looking for metabolomics service this page will help guide you to the right lab. The table below lists all of the metabolites that the center regularly sees and in which labs they are detected in. If your compound of interest is not on the list just contact us and we can still help you out. There are lots more compounds we can detect and many of our labs are open for method development projects if it is something new.

Press Ctrl + F to search the list

Compound Name	PubChem CID	Platform	Lab	Exact Mass	Formula
15-HETrE (C20:3n6)	25063347	Lipid Mediators	Newman	350.209	C20H30O5
RvE1 (C20:5n3)	25063347	Lipid Mediators	Newman	350.209	C20H30O5
Osbonate (C22:5n6)	6441454	Lipid Mediators	Newman	330.256	C22H34O2
Nonadeca-10(Z)-enoate (C19:1n9)	5312513	Lipid Mediators	Newman	296.272	C19H36O2
16(17)-EpDoPE (C22:6n3)	14392758	Lipid Mediators	Newman	344.235	C22H32O3
15-HpETE (C20:4n6)	5280893	Lipid Mediators	Newman	336.23	C20H32O4
PGD2 (C20:4n6)	448457	Lipid Mediators	Newman	352.225	C20H32O5
LTB5 (C20:5n3)	5283125	Lipid Mediators	Newman	334.214	C20H30O4
Eicosenoate (C20:1n9)	5282768	Lipid Mediators	Newman	310.287	C20H38O2
12(13)-EpODE (C18:3n3)	16061061	Lipid Mediators	Newman	294.219	C18H30O3
14,15-DiHETE (C20:5n3)	16061119	Lipid Mediators	Newman	336.23	C20H32O4

Compound Name	PubChem CID	Platform	Lab	Exact Mass	Formula
OEA (C18:1n9)	5283454	Lipid Mediators	Newman	325.298	C20H39NO2
PGE3 (C20:5n3)	5280937	Lipid Mediators	Newman	350.209	C20H30O5
12(13)-EpOME (C18:2n6)	5356421	Lipid Mediators	Newman	296.235	C18H32O3
12,13-DiHOME (C18:2n6)	10236635	Lipid Mediators	Newman	314.246	C18H34O4
8(9)-EpETrE (C20:4n6)	5283203	Lipid Mediators	Newman	320.235	C20H32O3
8,9-DiHETrE (C20:4n6)	5283144	Lipid Mediators	Newman	338.246	C20H34O4
1-AG (C20:4n6)	16019980	Lipid Mediators	Newman	378.277	C23H38O4
α Linolenate (C18:3n3)	5282822	Lipid Mediators	Newman	278.225	C18H30O2
11(12)-EpETrE (C20:4n6)	5283204	Lipid Mediators	Newman	320.235	C20H32O3
19,20-DiHDoPE (C22:6n3)	16061148	Lipid Mediators	Newman	362.246	C22H34O4
DEA (C22:4n6)	5282273	Lipid Mediators	Newman	375.314	C24H41NO2
5-HEPE (C20:5n3)	6439678	Lipid Mediators	Newman	318.219	C20H30O3
11-HETE (C20:4n6)	5312981	Lipid Mediators	Newman	320.235	C20H32O3
DHEA (C22:6n3)	44373477	Lipid Mediators	Newman	371.282	C24H37NO2
5,6-DiHETrE (C20:4n6)	5283142	Lipid Mediators	Newman	338.246	C20H34O4
PGE1 (C20:3n6)	5280723	Lipid Mediators	Newman	354.241	C20H34O5
17(18)-EpETE (C20:5n3)	16061089	Lipid Mediators	Newman	318.219	C20H30O3
aLEA (C18:3n3)	5283449	Lipid Mediators	Newman	321.267	C20H35NO2

Compound Name	PubChem CID	Platform	Lab	Exact Mass	Formula
15(16)-EpODE (C18:3n3)	16061062	Lipid Mediators	Newman	294.219	C18H30O3
13-HODE (C18:2n6)	6443013	Lipid Mediators	Newman	296.235	C18H32O3
Dihomo-γ-linolenate (C20:3n6)	5280581	Lipid Mediators	Newman	306.256	C20H34O2
NO-Gly (C18:1n9)	6436908	Lipid Mediators	Newman	339.277	C20H37NO3
PEA (C16:0)	4671	Lipid Mediators	Newman	299.282	C18H37NO2
2-LG (C18:2n6)	5365676	Lipid Mediators	Newman	354.277	C21H38O4
Palmitate (C16:0)	985	Lipid Mediators	Newman	256.24	C16H32O2
Lipoxin A4 (C20:4n6)	5280914	Lipid Mediators	Newman	352.225	C20H32O5
Timnodonate; EPA (C20:5n3)	446284	Lipid Mediators	Newman	302.225	C20H30O2
Bovinate (C18:2(9c,11t)-CLA)	5280644	Lipid Mediators	Newman	280.24	C18H32O2
13-HpODE (C18:2n6)	5280720	Lipid Mediators	Newman	312.23	C18H32O4
9-HpODE (C18:2n6)	6439847	Lipid Mediators	Newman	312.23	C18H32O4
13-KODE (C18:2n6)	6446027	Lipid Mediators	Newman	294.219	C18H30O3
Stearidonate (C18:4n3)	5282837	Lipid Mediators	Newman	276.209	C18H28O2
15-HETE (C20:4n6)	5280724	Lipid Mediators	Newman	320.235	C20H32O3
9(10)-EpODE (C18:3n3)	16061060	Lipid Mediators	Newman	294.219	C18H30O3
9-HETE (C20:4n6)	5312978	Lipid Mediators	Newman	320.235	C20H32O3
Heptadecanoate (C17:0)	10465	Lipid Mediators	Newman	270.256	C17H34O2

Compound Name	PubChem CID	Platform	Lab	Exact Mass	Formula
PGD2 EA (PGD2)	5283120	Lipid Mediators	Newman	395.267	C22H37NO5
LTB4 (C20:4n6)	5280888	Lipid Mediators	Newman	370.236	C20H34O6
6-keto-PGF1a (C20:4n6)	5280888	Lipid Mediators	Newman	370.236	C20H34O6
5-HETE (C20:4n6)	5280733	Lipid Mediators	Newman	320.235	C20H32O3
13-HOTE (C18:3n3)	10469728	Lipid Mediators	Newman	294.219	C18H30O3
LEA (C18:2n6)	5283446	Lipid Mediators	Newman	323.282	C20H37NO2
AEA (C20:4n6)	5281969	Lipid Mediators	Newman	347.282	C22H37NO2
15,16-DiHODE (C18:3n3)	16061068	Lipid Mediators	Newman	312.23	C18H32O4
11,12-DiHETrE (C20:4n6)	5283146	Lipid Mediators	Newman	338.246	C20H34O4
9-KODE (C18:2n6)	9839084	Lipid Mediators	Newman	294.219	C18H30O3
Cervonate; DHA (C22:6n3)	445580	Lipid Mediators	Newman	328.24	C22H32O2
12-HEPE (C20:5n3)	10041593	Lipid Mediators	Newman	318.219	C20H30O3
9,12,13-TriHOME (C18:2n6)	9858729	Lipid Mediators	Newman	330.241	C18H34O5
5-KETE (C20:4n6)	5283159	Lipid Mediators	Newman	318.219	C20H30O3
8-HETE (C20:4n6)	5283154	Lipid Mediators	Newman	320.235	C20H32O3
20-HETE (C20:4n6)	5283157	Lipid Mediators	Newman	320.235	C20H32O3
8,15-DiHETE (C20:4n6)	5312973	Lipid Mediators	Newman	336.23	C20H32O4
9-HODE (C18:2n6)	5282945	Lipid Mediators	Newman	296.235	C18H32O3

Compound Name	PubChem CID	Platform	Lab	Exact Mass	Formula
9,10,13-TriHOME (C18:2n6)	5282965	Lipid Mediators	Newman	330.241	C18H34O5
RvD1 (C22:6n3)	16061135	Lipid Mediators	Newman	376.225	C22H32O5
19(20)-EpDoPE (C22:6n3)	11631565	Lipid Mediators	Newman	344.235	C22H32O3
SEA (C18:0)	27902	Lipid Mediators	Newman	327.314	C20H41NO2
LTE4 (C20:4n6)	5280749	Lipid Mediators	Newman	439.239	C23H37NO5S
Linoleate (C18:2n6)	5280450	Lipid Mediators	Newman	280.24	C18H32O2
Laurate (C12:0)	3893	Lipid Mediators	Newman	200.178	C12H24O2
PGB2 (C20:4n6)	5288144	Lipid Mediators	Newman	334.214	C20H30O4
11,12,15 TriHETrE (C20:4n6)	11954043	Lipid Mediators	Newman	354.241	C20H34O5
20-hydroxy-LTB4 (C20:4n6)	5280745	Lipid Mediators	Newman	352.225	C20H32O5
PGF2a (C20:4n6)	5283078	Lipid Mediators	Newman	354.241	C20H34O5
Stearate (C18:0)	5281	Lipid Mediators	Newman	284.272	C18H36O2
9,10-DiHODE (C18:3n3)	16061066	Lipid Mediators	Newman	312.23	C18H32O4
20-HETE EA (20-HETE)	35027640	Lipid Mediators	Newman	363.277	C22H37NO3
12(13)Ep-9-KODE (C18:2n6)	5283007	Lipid Mediators	Newman	310.214	C18H30O4
2-AG (C20:4n6)	5282280	Lipid Mediators	Newman	378.277	C23H38O4
12,13-DiHODE (C18:3n3)	16061067	Lipid Mediators	Newman	312.23	C18H32O4
14(15)-EpETE (C20:5n3)	16061088	Lipid Mediators	Newman	318.219	C20H30O3

Compound Name	PubChem CID	Platform	Lab	Exact Mass	Formula
9-HOTE (C18:3n3)	6439873	Lipid Mediators	Newman	294.219	C18H30O3
1-OG (C18:1n9)	12178130	Lipid Mediators	Newman	356.293	C21H40O4
Palmitoleate (C16:1n7)	5282745	Lipid Mediators	Newman	254.225	C16H30O2
Palmitelaidate (C16:1n7t)	5282745	Lipid Mediators	Newman	254.225	C16H30O2
Hepoxilin A3 (C20:4n6)	5460414	Lipid Mediators	Newman	336.23	C20H32O4
17-HDoHE (C22:6n3)	6439179	Lipid Mediators	Newman	344.235	C22H32O3
20-carboxy-LTB4 (C20:4n6)	5280877	Lipid Mediators	Newman	366.204	C20H30O6
14,15-DiHETrE (C20:4n6)	5283147	Lipid Mediators	Newman	338.246	C20H34O4
Myristate (C14:0)	11005	Lipid Mediators	Newman	228.209	C14H28O2
δ 12-PGJ2 (C20:4n6)	5280885	Lipid Mediators	Newman	334.214	C20H30O4
Adrenate (C22:4n6)	5282844	Lipid Mediators	Newman	332.272	C22H36O2
DGLA EA (C20:3n6)	5282272	Lipid Mediators	Newman	349.298	C22H39NO2
Mead acid (C20:3n9)	5312531	Lipid Mediators	Newman	306.256	C20H34O2
2-OG (C18:1n9)	5319879	Lipid Mediators	Newman	356.293	C21H40O4
PGJ2 (C20:4n6)	5280884	Lipid Mediators	Newman	334.214	C20H30O4
Vaccenate (C18:1n7)	5281127	Lipid Mediators	Newman	282.256	C18H34O2
5,15-DiHETE (C20:4n6)	5283158	Lipid Mediators	Newman	336.23	C20H32O4
15-deoxy PGJ2 (C20:4n6)	5311211	Lipid Mediators	Newman	316.204	C20H28O3

Compound Name	PubChem CID	Platform	Lab	Exact Mass	Formula
6-trans-LTB4 (C20:4n6)	5283128	Lipid Mediators	Newman	336.23	C20H32O4
γ-Linolenate (C18:3n6)	5280933	Lipid Mediators	Newman	278.225	C18H30O2
1-LG (C18:2n6)	6436630	Lipid Mediators	Newman	354.277	C21H38O4
15-HEPE (C20:5n3)	5353282	Lipid Mediators	Newman	318.219	C20H30O3
14(15)-EpETrE (C20:4n6)	11954058	Lipid Mediators	Newman	336.23	C20H32O4
Pentadecanoate (C15:0)	13849	Lipid Mediators	Newman	242.225	C15H30O2
PGF2a EA (PGF2a)	5283076	Lipid Mediators	Newman	397.283	C22H39NO5
9,10-DiHOME (C18:2n6)	9966640	Lipid Mediators	Newman	314.246	C18H34O4
9(10)-EpOME (C18:2n6)	9966640	Lipid Mediators	Newman	314.246	C18H34O4
PGE2 (C20:4n6)	5280360	Lipid Mediators	Newman	352.225	C20H32O5
TXB2 (C20:4n6)	5283137	Lipid Mediators	Newman	370.236	C20H34O6
17,18-DiHETE (C20:5n3)	16061120	Lipid Mediators	Newman	336.23	C20H32O4
15-KETE (C20:4n6)	5280701	Lipid Mediators	Newman	318.219	C20H30O3
NA-Gly (20:4n6)	5283389	Lipid Mediators	Newman	361.262	C22H35NO3
Clupanodonate; DPA (C22:5n3)	5497182	Lipid Mediators	Newman	330.256	C22H34O2
Arachidonate (C20:4n6)	444899	Lipid Mediators	Newman	304.24	C20H32O2
12-HpETE (C20:4n6)	5280892	Lipid Mediators	Newman	336.23	C20H32O4
12-HETE (C20:4n6)	5312983	Lipid Mediators	Newman	320.235	C20H32O3

Compound Name	PubChem CID	Platform	Lab	Exact Mass	Formula
Oleate (C18:1n9)	445639	Lipid Mediators	Newman	282.256	C18H34O2
PGD3	5282260	Oxylipin profiling	Hammock	350.209	C20H30O5
Resolvin E1	10473088	Oxylipin profiling	Hammock	350.209	C20H30O5
allopregnanolone	31402	Neurosteroids profiling	Hammock	318.256	C21H34O2
16(17)-EpDPE	14392758	Oxylipin profiling	Hammock	344.235	C22H32O3
PGD2	448457	Oxylipin profiling	Hammock	352.225	C20H32O5
LTB5	5283125	Oxylipin profiling	Hammock	334.214	C20H30O4
14,15-DiHETE	16061119	Oxylipin profiling	Hammock	336.23	C20H32O4
PGE3	5280937	Oxylipin profiling	Hammock	350.209	C20H30O5
12(13)-EpOME	5356421	Oxylipin profiling	Hammock	296.235	C18H32O3
PGD1	5280936	Oxylipin profiling	Hammock	354.241	C20H34O5
12,13-DiHOME	10236635	Oxylipin profiling	Hammock	314.246	C18H34O4
3alpha,5alpha-tetrahydrodeoxycorticosterone	91475	Neurosteroids profiling	Hammock	334.251	C21H34O3
8(9)-EpETrE alt	40490662	Oxylipin profiling	Hammock	320.235	C20H32O3
8(9)-EpETrE	1901	Oxylipin profiling	Hammock	320.235	C20H32O3
13(14)-EpDPE	11674605	Oxylipin profiling	Hammock	344.235	C22H32O3
8,9-DiHETrE	5283144	Oxylipin profiling	Hammock	338.246	C20H34O4
11,12-DiHETE	16061121	Oxylipin profiling	Hammock	336.23	C20H32O4

Compound Name	PubChem CID	Platform	Lab	Exact Mass	Formula
7,8-DiHDPE	16061144	Oxylipin profiling	Hammock	362.246	C22H34O4
9(10)-EpOME	6246154	Oxylipin profiling	Hammock	296.235	C18H32O3
24R, 25-(OH)2D3	5283748	Vitamin D profiling	Hammock	416.329	C27H44O3
19,20-DiHDPE	16061148	Oxylipin profiling	Hammock	362.246	C22H34O4
5-HEPE	6439678	Oxylipin profiling	Hammock	318.219	C20H30O3
11-HETE	5312981	Oxylipin profiling	Hammock	320.235	C20H32O3
5,6-DiHETrE	5283142	Oxylipin profiling	Hammock	338.246	C20H34O4
1α,25- (OH)2D3	5280453	Vitamin D profiling	Hammock	416.329	C27H44O3
PGE1	5280723	Oxylipin profiling	Hammock	354.241	C20H34O5
17(18)-EpETE	131367	Oxylipin profiling	Hammock	318.219	C20H30O3
12-oxo-ETE	5283162	Oxylipin profiling	Hammock	318.219	C20H30O3
15(16)-EpODE	16061062	Oxylipin profiling	Hammock	294.219	C18H30O3
13-HODE	6443013	Oxylipin profiling	Hammock	296.235	C18H32O3
15(S)-HETrE	5283145	Oxylipin profiling	Hammock	322.251	C20H34O3
LXA4	5280914	Oxylipin profiling	Hammock	352.225	C20H32O5
14(15)-EpETrE	1431	Oxylipin profiling	Hammock	320.235	C20H32O3
13-oxo-ODE	6446027	Oxylipin profiling	Hammock	294.219	C18H30O3
15-HETE	5280724	Oxylipin profiling	Hammock	320.235	C20H32O3

Compound Name	PubChem CID	Platform	Lab	Exact Mass	Formula
9(10)-EpODE	16061060	Oxylipin profiling	Hammock	294.219	C18H30O3
25-(OH)D3	5283731	Vitamin D profiling	Hammock	400.334	C27H44O2
Cortisol	5754	Neurosteroids profiling	Hammock	362.209	C21H30O5
9-HETE	5312978	Oxylipin profiling	Hammock	320.235	C20H32O3
6-keto-PGF1a	5280888	Oxylipin profiling	Hammock	370.236	C20H34O6
5-HETE	5280733	Oxylipin profiling	Hammock	320.235	C20H32O3
25-(OH)D2	5710148	Vitamin D profiling	Hammock	412.334	C28H44O2
13-HOTrE	16061072	Oxylipin profiling	Hammock	294.219	C18H30O3
13,14-DiHDPE	16061146	Oxylipin profiling	Hammock	362.246	C22H34O4
15,16-DiHODE	16061068	Oxylipin profiling	Hammock	312.23	C18H32O4
11,12-DiHETrE	5283146	Oxylipin profiling	Hammock	338.246	C20H34O4
9-oxo-ODE	9839084	Oxylipin profiling	Hammock	294.219	C18H30O3
12-HEPE	10041593	Oxylipin profiling	Hammock	318.219	C20H30O3
9,12,13-TriHOME	9858729	Oxylipin profiling	Hammock	330.241	C18H34O5
5-oxo-ETE	5283159	Oxylipin profiling	Hammock	318.219	C20H30O3
8-HETE	6438380	Oxylipin profiling	Hammock	320.235	C20H32O3
LTB3	6439476	Oxylipin profiling	Hammock	338.246	C20H34O4
20-HETE	5283157	Oxylipin profiling	Hammock	320.235	C20H32O3

Compound Name	PubChem CID	Platform	Lab	Exact Mass	Formula
8,15-DiHETE	53480358	Oxylipin profiling	Hammock	336.23	C20H32O4
9-HODE	5282945	Oxylipin profiling	Hammock	296.235	C18H32O3
9,10,13-TriHOME	14968868	Oxylipin profiling	Hammock	330.241	C18H34O5
8,9-DiHETE	16061118	Oxylipin profiling	Hammock	336.23	C20H32O4
10,11-DiHDPE	16061145	Oxylipin profiling	Hammock	362.246	C22H34O4
7(8)-EpDPE	11653103	Oxylipin profiling	Hammock	344.235	C22H32O3
Corticosterone	5753	Neurosteroids profiling	Hammock	346.214	C21H30O4
Pregnenolone	8955	Neurosteroids profiling	Hammock	316.24	C21H32O2
19(20)-EpDPE	11631565	Oxylipin profiling	Hammock	344.235	C22H32O3
Ganaxolone	38022	Neurosteroids profiling	Hammock	332.272	C22H36O2
Aldosterone	5839	Neurosteroids profiling	Hammock	360.194	C21H28O5
PGB2	5280881	Oxylipin profiling	Hammock	334.214	C20H30O4
20-OH-LTB4	5280745	Oxylipin profiling	Hammock	352.225	C20H32O5
PGF2a	20849107	Oxylipin profiling	Hammock	354.241	C20H34O5
11(12)-EpETE	53394022	Oxylipin profiling	Hammock	318.219	C20H30O3
9,10-DiHODE	16061066	Oxylipin profiling	Hammock	312.23	C18H32O4
EKODE	53394018	Oxylipin profiling	Hammock	310.214	C18H30O4
12,13-DiHODE	16061067	Oxylipin profiling	Hammock	312.23	C18H32O4

Compound Name	PubChem CID	Platform	Lab	Exact Mass	Formula
14(15)-EpETE	53394073	Oxylipin profiling	Hammock	318.219	C20H30O3
9-HOTrE	6439873	Oxylipin profiling	Hammock	294.219	C18H30O3
Progesterone	5994	Neurosteroids profiling	Hammock	314.225	C21H30O2
17-HDoHE	6439179	Oxylipin profiling	Hammock	344.235	C22H32O3
20-COOH-LTB4	5280877	Oxylipin profiling	Hammock	366.204	C20H30O6
14,15-DiHETrE	5283147	Oxylipin profiling	Hammock	338.246	C20H34O4
PGJ2	5280884	Oxylipin profiling	Hammock	334.214	C20H30O4
5,15-DiHETE	5283158	Oxylipin profiling	Hammock	336.23	C20H32O4
5(6)-EpETrE	1778	Oxylipin profiling	Hammock	320.235	C20H32O3
15-deoxy-PGJ2	5311211	Oxylipin profiling	Hammock	316.204	C20H28O3
LTB4	5280492	Oxylipin profiling	Hammock	336.23	C20H32O4
d4-LTB4	16061113	Oxylipin profiling	Hammock	336.23	C20H32O4
6-trans-LTB4	5283128	Oxylipin profiling	Hammock	336.23	C20H32O4
THF diol	8064	Oxylipin profiling	Hammock	90.068	C4H10O2
Deoxycortisol	440707	Neurosteroids profiling	Hammock	346.214	C21H30O4
15-HEPE	5353282	Oxylipin profiling	Hammock	318.219	C20H30O3
8-HEPE	53394250	Oxylipin profiling	Hammock	318.219	C20H30O3
9,10-DiHOME	9966640	Oxylipin profiling	Hammock	314.246	C18H34O4

Compound Name	PubChem CID	Platform	Lab	Exact Mass	Formula
PGE2	5280360	Oxylipin profiling	Hammock	352.225	$C_{20}H_{32}O_5$
d4-PGE2	5283031	Oxylipin profiling	Hammock	352.225	$C_{20}H_{32}O_5$
TXB2	5283137	Oxylipin profiling	Hammock	370.236	$C_{20}H_{34}O_6$
d4-TXB2	16061116	Oxylipin profiling	Hammock	370.236	$C_{20}H_{34}O_6$
17,18-DiHETE	16061120	Oxylipin profiling	Hammock	336.23	$C_{20}H_{32}O_4$
15-oxo-ETE	5280701	Oxylipin profiling	Hammock	318.219	$C_{20}H_{30}O_3$
8(9)-EpETE	53394044	Oxylipin profiling	Hammock	318.219	$C_{20}H_{30}O_3$
16,17-DiHDPE	16061147	Oxylipin profiling	Hammock	362.246	$C_{22}H_{34}O_4$
10(11)-EpDPE	11638767	Oxylipin profiling	Hammock	344.235	$C_{22}H_{32}O_3$
1α, 25-(OH)2D2	6437855	Vitamin D profiling	Hammock	428.329	$C_{28}H_{44}O_3$
12-HETE	5312983	Oxylipin profiling	Hammock	320.235	$C_{20}H_{32}O_3$
11,12-,15-TriHETrE	11954042	Oxylipin profiling	Hammock		
5,6-DiHETE	na	Oxylipin profiling	Hammock		(chemspider 444[illegible]
4,5-DiHDPE	na	Oxylipin profiling	Hammock		
12(13)-EpODE	na	Oxylipin profiling	Hammock		
11(12)-EpETrE	na	Oxylipin profiling	Hammock		
d4-6-keto-PGF1a	na	Oxylipin profiling	Hammock		
d11-14,15-DiHETrE	na	Oxylipin profiling	Hammock		

Compound Name	PubChem CID	Platform	Lab	Exact Mass	Formula
d6-20-HETE	na	Oxylipin profiling	Hammock		
d4-9-HODE	na	Oxylipin profiling	Hammock		
d8-12-HETE	na	Oxylipin profiling	Hammock		
d8-5-HETE	na	Oxylipin profiling	Hammock		
d11-11(12)-EpETrE	na	Oxylipin profiling	Hammock		
d4-9(10)-EpOME	na	Oxylipin profiling	Hammock		
d8-AA	na	Oxylipin profiling	Hammock		
Androstenedione	6432604	Steroid profiling	Gaikwad	286.193	C19H26O2
4-hydroxy-estradiol-2-glutatione	NA	Steroid profiling	Gaikwad		
4-methoxy-estradiol	68578	Steroid profiling	Gaikwad	302.188	C19H26O3
2-hydroxy-estrone-1-N-acetylcysteine	NA	Steroid profiling	Gaikwad		
2-methoxy-estradiol	66414	Steroid profiling	Gaikwad	302.188	C19H26O3
2-hydroxy-estrone-1-glutatione	NA	Steroid profiling	Gaikwad		
2-hydroxy-estradiol	16219475	Steroid profiling	Gaikwad	288.173	C18H24O3
Estrone	6710658	Steroid profiling	Gaikwad	270.162	C18H22O2
4-hydroxy-estradiol-1-N-7-guanine	NA	Steroid profiling	Gaikwad		
2-hydroxy-estrone-6-N-3-adenine	NA	Steroid profiling	Gaikwad		
2-hydroxy-estradiol-4-N-acetylcysteine	NA	Steroid profiling	Gaikwad		

Compound Name	PubChem CID	Platform	Lab	Exact Mass	Formula
Estrone-Sulfate	10861069	Steroid profiling	Gaikwad	350.119	C18H22O5S
4-hydroxy-estradiol-2-N-acetylcysteine	NA	Steroid profiling	Gaikwad		
4-hydroxy-estrone-2-cysteine	NA	Steroid profiling	Gaikwad		
4-hydroxy-estrone-1-N-7-guanine	9889189	Steroid profiling	Gaikwad	435.191	C23H25N5O4
4-hydroxy-estrone-1-N-3-adenine	NA	Steroid profiling	Gaikwad		
4-hydroxy-estradiol-1-N-3-adenine	NA	Steroid profiling	Gaikwad		
2-OH-3-methoxy-estradiol	NA	Steroid profiling	Gaikwad		
Testosterone	19615208	Steroid profiling	Gaikwad	288.209	C19H28O2
16α-hydroxy-estradiol	16757678	Steroid profiling	Gaikwad	288.173	C18H24O3
4-methoxy-estrone	168393	Steroid profiling	Gaikwad	357.194	C8H8.C7H12O2.(
4-hydroxy-estradiol	49823447	Steroid profiling	Gaikwad	288.173	C18H24O3
2-hydroxy-estradiol-1-glutatione	10257895	Steroid profiling	Gaikwad	593.241	C28H39N3O9S
2-hydroxy-estradiol-6-N-3-adenine	NA	Steroid profiling	Gaikwad		
2-hydroxy-estrone	54328033	Steroid profiling	Gaikwad	286.157	C18H22O3
4-hydroxy-estradiol-2-cysteine	NA	Steroid profiling	Gaikwad		
4-hydroxy-estrone-2-glutatione	NA	Steroid profiling	Gaikwad		
2-hydroxy-estradiol-1+4-cysteine	NA	Steroid profiling	Gaikwad		
2-hydroxy-estrone-1-cysteine	NA	Steroid profiling	Gaikwad		

Compound Name	PubChem CID	Platform	Lab	Exact Mass	Formula
2-hydroxy-estradiol-4-glutatione	10393673	Steroid profiling	Gaikwad	593.241	C28H39N3O9S
Estradiol	43912931	Steroid profiling	Gaikwad	272.178	C18H24O2
2-methoxy-estrone	9686	Steroid profiling	Gaikwad	300.173	C19H24O3
4-hydroxy-estrone-2-N-acetylcysteine	NA	Steroid profiling	Gaikwad		
16α-hydroxy-estrone	115116	Steroid profiling	Gaikwad	286.157	C18H22O3
2-hydroxy-estrone-4-cysteine	NA	Steroid profiling	Gaikwad		
4-hydroxy-estrone	9971251	Steroid profiling	Gaikwad	286.157	C18H22O3
2-OH-3-methoxy-estrone	13831793	Steroid profiling	Gaikwad	300.173	C19H24O3
2-hydroxy-estrone-4-N-acetylcysteine	NA	Steroid profiling	Gaikwad		
2-hydroxy-estradiol-1-N-acetylcysteine	NA	Steroid profiling	Gaikwad		
2-hydroxy-estrone-4-glutatione	NA	Steroid profiling	Gaikwad		
quinic acid	6508	Primary Metabolites	Fiehn	192.063	C7H12O6
benzyl thiocyanate	18170	Primary Metabolites	Fiehn	149.03	C8H7NS
5-Dihydrocortisol	164838	Primary Metabolites	Fiehn	364.225	C21H32O5
α-D-glucosamine phosphate	121988	Primary Metabolites	Fiehn	259.046	C6H14NO8P
4-androsten-3,17-dione	6128	Primary Metabolites	Fiehn	286.193	C19H26O2
glycolic acid	757	Primary Metabolites	Fiehn	76.016	C2H4O3
phytosphingosine	122121	Primary Metabolites	Fiehn	317.293	C18H39NO3

Compound Name	PubChem CID	Platform	Lab	Exact Mass	Formula
N-methylaniline	7515	Primary Metabolites	Fiehn	107.073	C7H9N
2-hydroxybutyric acid	11266	Primary Metabolites	Fiehn	104.047	C4H8O3
2-hydroxybutanoic acid	440864	Primary Metabolites	Fiehn	104.047	C4H8O3
acenaphthenequinone	6724	Primary Metabolites	Fiehn	182.037	C12H6O2
(R)-(+)-1,2-dithiolane-3-pentanoic acid (lipoic acid)	6112	Primary Metabolites	Fiehn	206.044	C8H14O2S2
L-Isoleucine	6306	Primary Metabolites	Fiehn	131.095	C6H13NO2
3-aminopropionitrile	1647	Primary Metabolites	Fiehn	70.053	C3H6N2
L-ornithine	6262	Primary Metabolites	Fiehn	132.09	C5H12N2O2
hesperetin	3593	Primary Metabolites	Fiehn	302.079	C16H14O6
3-hydroxypropanoic acid	68152	Primary Metabolites	Fiehn	90.032	C3H6O3
4-aminobenzoic acid (p-Aminobenzoic acid)	978	Primary Metabolites	Fiehn	137.048	C7H7NO2
loganin	16212335	Primary Metabolites	Fiehn	390.153	C17H26O10
2-METHYLGLUTARIC ACID	12046	Primary Metabolites	Fiehn	146.058	C6H10O4
neohesperidin	442439	Primary Metabolites	Fiehn	610.19	C28H34O15
Tricetin	5281701	Primary Metabolites	Fiehn	302.043	C15H10O7
2-Deoxyerythritol	18302	Primary Metabolites	Fiehn	106.063	C4H10O3
phosphoglycolic acid	529	Primary Metabolites	Fiehn	155.982	C2H5O6P
Acylcarnitine C18:1	53477789	Complex lipids	Fiehn	425.351	C25H47NO4

Compound Name	PubChem CID	Platform	Lab	Exact Mass	Formula
LysoPC(16:0)	460602	Complex lipids	Fiehn	495.332	C24H50NO7P
beta-Hydroxymyristic acid	16064	Primary Metabolites	Fiehn	244.204	C14H28O3
3beta-Hydroxy-5beta-pregnane-20-one	228491	Primary Metabolites	Fiehn	318.256	C21H34O2
5β-PREGNAN-3β-OL-20-ONE	31402	Primary Metabolites	Fiehn	318.256	C21H34O2
hydroxylamine	787	Primary Metabolites	Fiehn	33.021	H3NO
D-(glycerol 1-phosphate)	439162	Primary Metabolites	Fiehn	172.014	C3H9O6P
glycerol 1-phosphate	754	Primary Metabolites	Fiehn	172.014	C3H9O6P
L-alpha-Glycerophophate	439276	Primary Metabolites	Fiehn	172.014	C3H9O6P
L-threonine	6288	Primary Metabolites	Fiehn	119.058	C4H9NO3
L-allothreonine	99289	Primary Metabolites	Fiehn	119.058	C4H9NO3
threonine	205	Primary Metabolites	Fiehn	119.058	C4H9NO3
4-hydroxybenzyl cyanide	26548	Primary Metabolites	Fiehn	133.053	C8H7NO
Gentiobiose	92807	Primary Metabolites	Fiehn	342.116	C12H22O11
p-benzoquinone	4650	Primary Metabolites	Fiehn	108.021	C6H4O2
β-glutamic acid	73064	Primary Metabolites	Fiehn	147.053	C5H9NO4
2-Monopalmitin	123409	Primary Metabolites	Fiehn	330.277	C19H38O4
3-methyloxyindole (3-Methyl-2-oxindole)	150923	Primary Metabolites	Fiehn	147.068	C9H9NO
azelaic acid	2266	Primary Metabolites	Fiehn	188.105	C9H16O4

Compound Name	PubChem CID	Platform	Lab	Exact Mass	Formula
5-methoxypsoralen	2355	Primary Metabolites	Fiehn	216.042	C12H8O4
phenylethylamine	1001	Primary Metabolites	Fiehn	121.089	C8H11N
cholic acid	221493	Primary Metabolites	Fiehn	408.288	C24H40O5
5-Hydroxyindole-2-carboxylic acid	88958	Primary Metabolites	Fiehn	177.043	C9H7NO3
6-phosphogluconic acid	91493	Primary Metabolites	Fiehn	276.025	C6H13O10P
triacontanoic acid methyl ester	12400	Primary Metabolites	Fiehn	466.475	C31H62O2
cis-11-Eicosenoic acid	5282768	Primary Metabolites	Fiehn	310.287	C20H38O2
3-hydroxy-L-proline	440575	Primary Metabolites	Fiehn	131.058	C5H9NO3
L-Malic acid	222656	Primary Metabolites	Fiehn	134.022	C4H6O5
D-malic acid	92824	Primary Metabolites	Fiehn	134.022	C4H6O5
sorbose	6904	Primary Metabolites	Fiehn	180.063	C6H12O6
D-tagatose	92092	Primary Metabolites	Fiehn	180.063	C6H12O6
L- sorbose (D-Psicose)	1101	Primary Metabolites	Fiehn	180.063	C6H12O6
fructose	5984	Primary Metabolites	Fiehn	180.063	C6H12O6
dioctyl phthalate	8343	Primary Metabolites	Fiehn	390.277	C24H38O4
arbutin	440936	Primary Metabolites	Fiehn	272.09	C12H16O7
2-ketoisocaproic acid (alpha-ketoisocaproate)	70	Primary Metabolites	Fiehn	130.063	C6H10O3
4-methyl-5-thiazoleethanol	1136	Primary Metabolites	Fiehn	143.04	C6H9NOS

Compound Name	PubChem CID	Platform	Lab	Exact Mass	Formula
6-hydroxynicotinic acid	72924	Primary Metabolites	Fiehn	139.027	C6H5NO3
phytol (cis-Phytol)	6430833	Primary Metabolites	Fiehn	296.308	C20H40O
isopropyl β-D-1-thiogalactopyranoside	656894	Primary Metabolites	Fiehn	238.087	C9H18O5S
glycocyamine (Guanidoacetic acid)	763	Primary Metabolites	Fiehn	117.054	C3H7N3O2
aspirin (O-acetylsalicylic acid)	2244	Primary Metabolites	Fiehn	180.042	C9H8O4
4-aminobutyric acid (L-A-Amino-N-butyric acid)	119	Primary Metabolites	Fiehn	103.063	C4H9NO2
4-nitrophenol	980	Primary Metabolites	Fiehn	139.027	C6H5NO3
1-HYDROXYANTHRAQUINONE	8512	Primary Metabolites	Fiehn	224.047	C14H8O3
phenylpyruvate	997	Primary Metabolites	Fiehn	164.047	C9H8O3
TES	446188	Primary Metabolites	Fiehn	290.225	C19H30O2
3-Cyanoalanine	13538	Primary Metabolites	Fiehn	114.043	C4H6N2O2
β-cyano-L-alanine	439742	Primary Metabolites	Fiehn	114.043	C4H6N2O2
1-Hexadecanol	2682	Primary Metabolites	Fiehn	242.261	C16H34O
N,N-dimethyl-1,4-phenylenediamine	7472	Primary Metabolites	Fiehn	136.1	C8H12N2
O-phospho-L-serine (O-Phosphoserine)	68841	Primary Metabolites	Fiehn	185.009	C3H8NO6P
METHYL PHOSPHATE	13130	Primary Metabolites	Fiehn	111.993	CH5O4P
lanosterol	246983	Primary Metabolites	Fiehn	426.386	C30H50O
Androstanediol	32801	Primary Metabolites	Fiehn	292.24	C19H32O2

Compound Name	PubChem CID	Platform	Lab	Exact Mass	Formula
N-acetyl-L-phenylalanine	74839	Primary Metabolites	Fiehn	207.09	C11H13NO3
3-Hydroxypalmitic acid	301590	Primary Metabolites	Fiehn	272.235	C16H32O3
methyl cinnamate	637520	Primary Metabolites	Fiehn	162.068	C10H10O2
allo-inositol (myo-inositol)(muco-Inositol)	892	Primary Metabolites	Fiehn	180.063	C6H12O6
2-aminophenol	5801	Primary Metabolites	Fiehn	109.053	C6H7NO
trans-3,5-dimethoxy-4-hydroxycinnamaldehyde	5280802	Primary Metabolites	Fiehn	208.074	C11H12O4
piceatannol	667639	Primary Metabolites	Fiehn	244.074	C14H12O4
3,4-dihydroxyphenylacetic acid	547	Primary Metabolites	Fiehn	168.042	C8H8O4
DL-4-hydroxy-3-methoxymandelic acid (Vanillylmandelic acid)	1245	Primary Metabolites	Fiehn	198.053	C9H10O5
Zymosterol	92746	Primary Metabolites	Fiehn	384.339	C27H44O
3-(2-hydroxyphenyl)propanoic acid	873	Primary Metabolites	Fiehn	166.063	C9H10O3
N-benzyloxycarbonylglycine (Carbobenzyloxyglycine)	14349	Primary Metabolites	Fiehn	209.069	C10H11NO4
hexachlorobenzene	8370	Primary Metabolites	Fiehn	281.813	C6Cl6
aspartic acid	5960	Primary Metabolites	Fiehn	133.038	C4H7NO4
D-Aspartic acid	424	Primary Metabolites	Fiehn	133.038	C4H7NO4
4-isopropylbenzoic acid (CUMIC ACID)	10820	Primary Metabolites	Fiehn	164.084	C10H12O2
2,5-dihydroxybenzaldehyde	70949	Primary Metabolites	Fiehn	138.032	C7H6O3
phenylphosphoric acid	12793	Primary Metabolites	Fiehn	174.008	C6H7O4P

Compound Name	PubChem CID	Platform	Lab	Exact Mass	Formula
PE(22:6(4Z,7Z,10Z,13Z,16Z,19Z)/16:0)	52924862	Complex lipids	Fiehn	763.515	C43H74NO8P
5-methoxy-3-indoleacetic acid	18986	Primary Metabolites	Fiehn	205.074	C11H11NO3
phenylalanine	994	Primary Metabolites	Fiehn	165.079	C9H11NO2
phlorobenzophenone (2,4,6-Trihydroxybenzophenone)	77093	Primary Metabolites	Fiehn	230.058	C13H10O4
PC(P-16:0/18:1(9Z))	24779384	Complex lipids	Fiehn	743.583	C42H82NO7P
farnesol	1549107	Primary Metabolites	Fiehn	222.198	C15H26O
trans-trans-farnesol	445070	Primary Metabolites	Fiehn	222.198	C15H26O
D-(+)-Ribonic acid gamma-lactone	111064	Primary Metabolites	Fiehn	148.037	C5H8O5
Creatine	586	Complex lipids	Fiehn	131.069	C4H9N3O2
chlorogenic acid	6537496	Primary Metabolites	Fiehn	354.095	C16H18O9
taxifolin	439533	Primary Metabolites	Fiehn	304.058	C15H12O7
(+/-)-Taxifolin	471	Primary Metabolites	Fiehn	304.058	C15H12O7
N-methyltryptophan	914	Primary Metabolites	Fiehn	218.106	C12H14N2O2
N-2-fluorenylacetamide	5897	Primary Metabolites	Fiehn	223.1	C15H13NO
sucrose	5988	Primary Metabolites	Fiehn	342.116	C12H22O11
hexadecane	11006	Primary Metabolites	Fiehn	226.266	C16H34
L-asparagine	236	Primary Metabolites	Fiehn	132.053	C4H8N2O3
1,4-Cyclohexanedione	12511	Primary Metabolites	Fiehn	112.052	C6H8O2

Compound Name	PubChem CID	Platform	Lab	Exact Mass	Formula
creatinine	588	Primary Metabolites	Fiehn	113.059	C4H7N3O
beta-Mannosylglycerate	5460194	Primary Metabolites	Fiehn	268.079	C9H16O9
Ala-Ala	5484352	Primary Metabolites	Fiehn	160.085	C6H12N2O3
D-Ala-D-Ala	601	Primary Metabolites	Fiehn	160.085	C6H12N2O3
nicotinamide (Nicotinacid-amide)	936	Primary Metabolites	Fiehn	122.048	C6H6N2O
Acylcarnitine C18:3	53477821	Complex lipids	Fiehn	421.319	C25H43NO4
β-glycerolphosphate (beta-Glycerophosphoric acid)	2526	Primary Metabolites	Fiehn	172.014	C3H9O6P
glycine-d5 deuterated	2723970	Primary Metabolites	Fiehn	75.032	C2H5NO2
glycine	750	Primary Metabolites	Fiehn	75.032	C2H5NO2
mono(2-ethylhexyl)phthalate	20393	Primary Metabolites	Fiehn	278.152	C16H22O4
4-hydroxy-3-methoxycinnamaldehyde	5280536	Primary Metabolites	Fiehn	178.063	C10H10O3
2-Butyne-1,4-diol	8066	Primary Metabolites	Fiehn	86.037	C4H6O2
prunin	92794	Primary Metabolites	Fiehn	434.121	C21H22O10
melibiose	440658	Primary Metabolites	Fiehn	342.116	C12H22O11
isomaltose	439193	Primary Metabolites	Fiehn	342.116	C12H22O11
Melibiose	11458	Primary Metabolites	Fiehn	342.116	C12H22O11
3-indoleacetonitrile	351795	Primary Metabolites	Fiehn	156.069	C10H8N2
ergosterol	444679	Primary Metabolites	Fiehn	396.339	C28H44O

Compound Name	PubChem CID	Platform	Lab	Exact Mass	Formula
estrone	5870	Primary Metabolites	Fiehn	270.162	C18H22O2
PC(20:4(5Z,8Z,11Z,14Z)/18:0)	52923291	Complex lipids	Fiehn	809.593	C46H84NO8P
PE(18:2(9Z,12Z)/18:1(9Z))	52924364	Complex lipids	Fiehn	741.531	C41H76NO8P
melatonin	896	Primary Metabolites	Fiehn	232.121	C13H16N2O2
uridine	6029	Primary Metabolites	Fiehn	244.07	C9H12N2O6
D-saccharic acid	33037	Primary Metabolites	Fiehn	210.038	C6H10O8
N-epsilon-Acetyl-L-lysine	92832	Primary Metabolites	Fiehn	188.116	C8H16N2O3
linolenic acid	5280934	Primary Metabolites	Fiehn	278.225	C18H30O2
phenylacetaldehyde	998	Primary Metabolites	Fiehn	120.058	C8H8O
PE(20:4(5Z,8Z,11Z,14Z)/16:0)	52924875	Complex lipids	Fiehn	739.515	C41H74NO8P
5-hydroxyindole-3-acetic acid	1826	Primary Metabolites	Fiehn	191.058	C10H9NO3
5-Aminoimidazole-4-carboxamide	9679	Primary Metabolites	Fiehn	126.054	C4H6N4O
PE(18:1(9Z)/16:0)	9546802	Complex lipids	Fiehn	717.531	C39H76NO8P
Leucrose	165577	Primary Metabolites	Fiehn	342.116	C12H22O11
4,2',4'-trihydroxychalcone	638278	Primary Metabolites	Fiehn	256.074	C15H12O4
cycloserine	401	Primary Metabolites	Fiehn	102.043	C3H6N2O2
2-Carboxybenzaldehyde	8406	Primary Metabolites	Fiehn	150.032	C8H6O3
2-Deoxytetronic acid	150929	Primary Metabolites	Fiehn	120.042	C4H8O4

Compound Name	PubChem CID	Platform	Lab	Exact Mass	Formula
tyramine	5610	Primary Metabolites	Fiehn	137.084	C8H11NO
9-phenanthrenol	10229	Primary Metabolites	Fiehn	194.073	C14H10O
N-acetyl-D-tryptophan	439917	Primary Metabolites	Fiehn	246.1	C13H14N2O3
benzoylformic acid	11915	Primary Metabolites	Fiehn	150.032	C8H6O3
D-sorbitol	5780	Primary Metabolites	Fiehn	182.079	C6H14O6
D-mannitol	6251	Primary Metabolites	Fiehn	182.079	C6H14O6
Pelargonic acid	8158	Primary Metabolites	Fiehn	158.131	C9H18O2
lipoamide (Thioctamide)	863	Primary Metabolites	Fiehn	205.06	C8H15NOS2
hypoxanthine	790	Primary Metabolites	Fiehn	136.039	C5H4N4O
tartaric acid	444305	Primary Metabolites	Fiehn	150.016	C4H6O6
D,L-Tartaric acid	875	Primary Metabolites	Fiehn	150.016	C4H6O6
L-methionine	6137	Primary Metabolites	Fiehn	149.051	C5H11NO2S
methionine	876	Primary Metabolites	Fiehn	149.051	C5H11NO2S
2-ketoadipate	71	Primary Metabolites	Fiehn	160.037	C6H8O5
PE(P-18:0/22:6(4Z,7Z,10Z,13Z,16Z,19Z))	42607458	Complex lipids	Fiehn	775.552	C45H78NO7P
4-hydroxybenzoic acid	135	Primary Metabolites	Fiehn	138.032	C7H6O3
PE(20:4(5Z,8Z,11Z,14Z)/18:0)	52924644	Complex lipids	Fiehn	767.547	C43H78NO8P
hexadecanoic acid methyl ester (methyl hexadecanoate)	8181	Primary Metabolites	Fiehn	270.256	C17H34O2

Compound Name	PubChem CID	Platform	Lab	Exact Mass	Formula
Dehydroepiandrosterone	76	Primary Metabolites	Fiehn	288.209	C19H28O2
trans-dehydroandrosterone	5881	Primary Metabolites	Fiehn	288.209	C19H28O2
Acylcarnitine C18:0	52922056	Complex lipids	Fiehn	427.366	C25H49NO4
ribulose-5-phosphate	439184	Primary Metabolites	Fiehn	230.019	C5H11O8P
L-canavanine	275	Primary Metabolites	Fiehn	176.091	C5H12N4O3
Maleamate	11187	Primary Metabolites	Fiehn	115.027	C4H5NO3
maleamic acid	5280451	Primary Metabolites	Fiehn	115.027	C4H5NO3
sarcosine	1088	Primary Metabolites	Fiehn	89.048	C3H7NO2
naringenin	439246	Primary Metabolites	Fiehn	272.068	C15H12O5
4-vinylphenol	62453	Primary Metabolites	Fiehn	120.058	C8H8O
Acylcarnitine C12:0	168381	Complex lipids	Fiehn	343.272	C19H37NO4
liquiritigenin (4',7-dihyroxyflavanone)	1889	Primary Metabolites	Fiehn	256.074	C15H12O4
3-hydroxyphenylacetic acid	12122	Primary Metabolites	Fiehn	152.047	C8H8O3
PE(22:6(4Z,7Z,10Z,13Z,16Z,19Z)/18:1(9Z))	52924844	Complex lipids	Fiehn	789.531	C45H76NO8P
O-methylthreonine	222906	Primary Metabolites	Fiehn	133.074	C5H11NO3
maltotriose	192826	Primary Metabolites	Fiehn	504.169	C18H32O16
acetanilide	904	Primary Metabolites	Fiehn	135.068	C8H9NO
S-carboxymethylcysteine	193653	Primary Metabolites	Fiehn	179.025	C5H9NO4S

Compound Name	PubChem CID	Platform	Lab	Exact Mass	Formula
4-hydroxypyridine	12290	Primary Metabolites	Fiehn	95.037	C5H5NO
N-Methyl-DL-alanine	4377	Primary Metabolites	Fiehn	103.063	C4H9NO2
Methyl jasmonate (methyljasmonate)	62388	Primary Metabolites	Fiehn	224.141	C13H20O3
adenine	190	Primary Metabolites	Fiehn	135.054	C5H5N5
1-Methyladenosine	16219662	Primary Metabolites	Fiehn	281.112	C11H15N5O4
benzene-1,2,4-triol	10787	Primary Metabolites	Fiehn	126.032	C6H6O3
2,3-DIHYDROXYPYRIDINE	28115	Primary Metabolites	Fiehn	111.032	C5H5NO2
resorcinol	5054	Primary Metabolites	Fiehn	110.037	C6H6O2
pantothenic acid	6613	Primary Metabolites	Fiehn	219.111	C9H17NO5
capric acid	2969	Primary Metabolites	Fiehn	172.146	C10H20O2
quinolinic acid (Pyridine-2,3-dicarboxylic acid)	1066	Primary Metabolites	Fiehn	167.022	C7H5NO4
Octadecanol	8221	Primary Metabolites	Fiehn	270.292	C18H38O
2,3-dihydroxybenzoic acid	19	Primary Metabolites	Fiehn	154.027	C7H6O4
isoxanthopterin	10729	Primary Metabolites	Fiehn	179.044	C6H5N5O2
geraniol	637566	Primary Metabolites	Fiehn	154.136	C10H18O
2,6-Diaminopimelic acid	865	Primary Metabolites	Fiehn	190.095	C7H14N2O4
O-succinylhomoserine	439406	Primary Metabolites	Fiehn	219.074	C8H13NO6
1,2-Didecanoylglycerol	1321	Primary Metabolites	Fiehn	400.319	C23H44O5

Compound Name	PubChem CID	Platform	Lab	Exact Mass	Formula
2,2-DIMETHYLSUCCINIC ACID	11701	Primary Metabolites	Fiehn	146.058	C6H10O4
Diglycerol	42953	Primary Metabolites	Fiehn	166.084	C6H14O5
PC(16:1(9Z)/16:1(9Z))	24778764	Complex lipids	Fiehn	729.531	C40H76NO8P
3-HYDROXYPYRIDINE	7971	Primary Metabolites	Fiehn	95.037	C5H5NO
inosine 5'-monophosphate	8582	Primary Metabolites	Fiehn	348.047	C10H13N4O8P
D-Fructose 6-phosphate	69507	Primary Metabolites	Fiehn	260.03	C6H13O9P
benzylsuccinic acid (DL-Benzylsuccinic acid)	3858	Primary Metabolites	Fiehn	208.074	C11H12O4
trans-aconitic acid	444212	Primary Metabolites	Fiehn	174.016	C6H6O6
cellobiose	10712	Primary Metabolites	Fiehn	342.116	C12H22O11
maltose	6255	Primary Metabolites	Fiehn	342.116	C12H22O11
lactose	84571	Primary Metabolites	Fiehn	342.116	C12H22O11
alpha tocopherol	14985	Primary Metabolites	Fiehn	430.381	C29H50O2
adipamide	12364	Primary Metabolites	Fiehn	144.09	C6H12N2O2
thymidine 5'-monophosphate	9700	Primary Metabolites	Fiehn	322.057	C10H15N2O8P
D (+)altrose	94780	Primary Metabolites	Fiehn	180.063	C6H12O6
D-allose (allose)	102288	Primary Metabolites	Fiehn	180.063	C6H12O6
D-(+)-Glucose	107526	Primary Metabolites	Fiehn	180.063	C6H12O6
aldohexose (generic)	24749	Primary Metabolites	Fiehn	180.063	C6H12O6

Compound Name	PubChem CID	Platform	Lab	Exact Mass	Formula
N-ethylmaleamic acid	5369191	Primary Metabolites	Fiehn	143.058	C6H9NO3
cyclohexylsulfamic acid	7533	Primary Metabolites	Fiehn	179.062	C6H13NO3S
cis-4-hydroxycyclohexanecarboxylic acid	151138	Primary Metabolites	Fiehn	144.079	C7H12O3
stigmasterol	5280794	Primary Metabolites	Fiehn	412.371	C29H48O
4-hydroxyquinoline-2-carboxylic acid (kynurenic acid)	3845	Primary Metabolites	Fiehn	189.043	C10H7NO3
D-(+) trehalose (α,α-Trehalose)	7427	Primary Metabolites	Fiehn	342.116	C12H22O11
xylitol	6912	Primary Metabolites	Fiehn	152.068	C5H12O5
D-arabitol	94154	Primary Metabolites	Fiehn	152.068	C5H12O5
ribitol (Adonitol)	827	Primary Metabolites	Fiehn	152.068	C5H12O5
beta-hydroxypyruvate (3-hydroxypyruvate)	964	Primary Metabolites	Fiehn	104.011	C3H4O4
1,3-Cyclohexanedione	10434	Primary Metabolites	Fiehn	112.052	C6H8O2
formononetin	5280378	Primary Metabolites	Fiehn	268.074	C16H12O4
carbamoyl-aspartate	279	Primary Metabolites	Fiehn	176.043	C5H8N2O5
L-histidine	6274	Primary Metabolites	Fiehn	155.069	C6H9N3O2
citraconic acid	643798	Primary Metabolites	Fiehn	130.027	C5H6O4
2-methylfumarate (mesaconate)	638129	Primary Metabolites	Fiehn	130.027	C5H6O4
4-hydroxymandelonitrile	166768	Primary Metabolites	Fiehn	149.048	C8H7NO2
3,5-dihydroxyphenylglycine (1α-2S-Dihydroxy Phenylglycine)	443586	Primary Metabolites	Fiehn	183.053	C8H9NO4

Compound Name	PubChem CID	Platform	Lab	Exact Mass	Formula
Acylcarnitine C24:1	n/a	Complex lipids	Fiehn		
4-methylumbelliferone	5280567	Primary Metabolites	Fiehn	176.047	C10H8O3
Sedoheptulose	5459879	Primary Metabolites	Fiehn	210.074	C7H14O7
methyl heptadecanoate	15609	Primary Metabolites	Fiehn	284.272	C18H36O2
3-hydroxyflavone	11349	Primary Metabolites	Fiehn	238.063	C15H10O3
5-Cholesten-3-beta-ol = Cholesterol	5997	Primary Metabolites	Fiehn	386.355	C27H46O
cholestrol d6	16213400	Primary Metabolites	Fiehn	386.355	C27H46O
cholesterol	304	Primary Metabolites	Fiehn	386.355	C27H46O
4-pyridoxic acid	6723	Primary Metabolites	Fiehn	183.053	C8H9NO4
Pipecolinic acid	849	Primary Metabolites	Fiehn	129.079	C6H11NO2
pipecolic acid	439227	Primary Metabolites	Fiehn	129.079	C6H11NO2
albendazole	2082	Primary Metabolites	Fiehn	265.088	C12H15N3O2S
α-D-Glucose 1-phosphate	439165	Primary Metabolites	Fiehn	260.03	C6H13O9P
Glucose-1-phosphate	65533	Primary Metabolites	Fiehn	260.03	C6H13O9P
Ethanolamine	700	Primary Metabolites	Fiehn	61.053	C2H7NO
D-galacturonic acid	84740	Primary Metabolites	Fiehn	194.043	C6H10O7
3-hydroxybenzaldehyde	101	Primary Metabolites	Fiehn	122.037	C7H6O2
cytidine-5'-monophosphate	6131	Primary Metabolites	Fiehn	323.052	C9H14N3O8P

Compound Name	PubChem CID	Platform	Lab	Exact Mass	Formula
homogentisic acid	780	Primary Metabolites	Fiehn	168.042	C8H8O4
Digalacturonic acid	439694	Primary Metabolites	Fiehn	370.075	C12H18O13
LysoPC(18:0)	497299	Complex lipids	Fiehn	523.364	C26H54NO7P
3-hydroxybenzoic acid	7420	Primary Metabolites	Fiehn	138.032	C7H6O3
nonanoic acid methyl ester (Methyl pelargonate)	15606	Primary Metabolites	Fiehn	172.146	C10H20O2
3-isochromanone	78092	Primary Metabolites	Fiehn	148.052	C9H8O2
N,N-dimethyl-L-histidine (N(alpha),N(alpha)-dimethyl-L-histidine)	440274	Primary Metabolites	Fiehn	183.101	C8H13N3O2
PC(P-16:0/20:4(5Z,8Z,11Z,14Z))	24779388	Complex lipids	Fiehn	765.567	C44H80NO7P
palmitic acid	985	Primary Metabolites	Fiehn	256.24	C16H32O2
PC(16:1(9Z)/16:0)	52922440	Complex lipids	Fiehn	731.547	C40H78NO8P
thymidine	5789	Primary Metabolites	Fiehn	242.09	C10H14N2O5
uracil	1174	Primary Metabolites	Fiehn	112.027	C4H4N2O2
benzoin	8400	Primary Metabolites	Fiehn	212.084	C14H12O2
(-)-Adenosine 3',5'-cyclic monophosphate	6076	Primary Metabolites	Fiehn	329.053	C10H12N5O6P
p-cresol	2879	Primary Metabolites	Fiehn	108.058	C7H8O
6-hydroxy caproic acid	14490	Primary Metabolites	Fiehn	132.079	C6H12O3
2-hydroxy-2-phenylacetic acid (Mandelic acid)	1292	Primary Metabolites	Fiehn	152.047	C8H8O3
mandelic acid	439616	Primary Metabolites	Fiehn	152.047	C8H8O3

Compound Name	PubChem CID	Platform	Lab	Exact Mass	Formula
methyl palmitoleate	643801	Primary Metabolites	Fiehn	268.24	C17H32O2
tropic acid	643328	Primary Metabolites	Fiehn	166.063	C9H10O3
ε-caprolactam	7768	Primary Metabolites	Fiehn	113.084	C6H11NO
lactulose	11333	Primary Metabolites	Fiehn	342.116	C12H22O11
methyl yellow	6053	Primary Metabolites	Fiehn	225.127	C14H15N3
GLUTARIC ACID (Glutarate)	743	Primary Metabolites	Fiehn	132.042	C5H8O4
methyl octanoate (octanoic acid methyl ester)	8091	Primary Metabolites	Fiehn	158.131	C9H18O2
Aminomalonic acid	100714	Primary Metabolites	Fiehn	119.022	C3H5NO4
3-METHYLBENZYL ALCOHOL	11476	Primary Metabolites	Fiehn	122.073	C8H10O
5-aminovaleric acid	138	Primary Metabolites	Fiehn	117.079	C5H11NO2
coniferyl alcohol	1549095	Primary Metabolites	Fiehn	180.079	C10H12O3
p-toluenesulfonic acid	6101	Primary Metabolites	Fiehn	172.019	C7H8O3S
Threonic acid	151152	Primary Metabolites	Fiehn	136.037	C4H8O5
2-Hydroxyvaleric acid	98009	Primary Metabolites	Fiehn	118.063	C5H10O3
N-acetyl-ornithine	439232	Primary Metabolites	Fiehn	174.1	C7H14N2O3
3-ureidopropionate	111	Primary Metabolites	Fiehn	132.053	C4H8N2O3
5-METHOXYTRYPTAMINE (methoxytryptamine)	1833	Primary Metabolites	Fiehn	190.111	C11H14N2O
DL-p-hydroxyphenyllactic acid	9378	Primary Metabolites	Fiehn	182.058	C9H10O4

Compound Name	PubChem CID	Platform	Lab	Exact Mass	Formula
L-(+) lactic acid	107689	Primary Metabolites	Fiehn	90.032	C3H6O3
lactic acid	612	Primary Metabolites	Fiehn	90.032	C3H6O3
Digitoxose	258531	Primary Metabolites	Fiehn	148.074	C6H12O4
kyotorphin	123804	Primary Metabolites	Fiehn	337.175	C15H23N5O4
shikimic acid	8742	Primary Metabolites	Fiehn	174.053	C7H10O5
hydrocortisone	5754	Primary Metabolites	Fiehn	362.209	C21H30O5
lactobionic acid	16219560	Primary Metabolites	Fiehn	358.111	C12H22O12
Mevalonic acid lactone	10428	Primary Metabolites	Fiehn	130.063	C6H10O3
N-gamma-acetyl-N-2-formyl-5-methoxykynurenamine (N-Acetyl-N-formyl-5-methoxykynurenamine)	171161	Primary Metabolites	Fiehn	264.111	C13H16N2O4
N-(2-hydroxyethyl)iminodiacetic acid	7152	Primary Metabolites	Fiehn	177.064	C6H11NO5
Ethyl cinnamate	7649	Primary Metabolites	Fiehn	176.084	C11H12O2
2-Ketovaleric acid	74563	Primary Metabolites	Fiehn	116.047	C5H8O3
L-lysine	5962	Primary Metabolites	Fiehn	146.106	C6H14N2O2
succinic acid	1110	Primary Metabolites	Fiehn	118.027	C4H6O4
heptadecanoic acid	10465	Primary Metabolites	Fiehn	270.256	C17H34O2
oxaloacetic acid	970	Primary Metabolites	Fiehn	132.006	C4H4O5
2′-deoxyadenosine 5′-monophosphate	621	Primary Metabolites	Fiehn	331.068	C10H14N5O6P
putrescine (1,4-Diaminobutane)	1045	Primary	Fiehn	88.1	C4H12N2

Compound Name	PubChem CID	Platform	Lab	Exact Mass	Formula
		Metabolites			
PC(16:0/16:0)	452110	Complex lipids	Fiehn	733.562	C40H80NO8P
4-hydroxyphenylpyruvic acid	979	Primary Metabolites	Fiehn	180.042	C9H8O4
terephthalic acid	7489	Primary Metabolites	Fiehn	166.027	C8H6O4
3-hydroxycinnamic acid (m-coumaric acid)	637541	Primary Metabolites	Fiehn	164.047	C9H8O3
3-Methylthiopropylamine (3-(Methylthio)-propylamine)	77743	Primary Metabolites	Fiehn	105.061	C4H11NS
2,3-Dimethylsuccinic acid	11848	Primary Metabolites	Fiehn	146.058	C6H10O4
4-Methylbenzyl alcohol	11505	Primary Metabolites	Fiehn	122.073	C8H10O
alpha-ketoglutaric acid (2-Ketoglutaric acid)	51	Primary Metabolites	Fiehn	146.022	C5H6O5
prunetin (4',5-dihydroxy-7-methoxyisoflavone)	5281804	Primary Metabolites	Fiehn	284.068	C16H12O5
Nicotianamine	9882882	Primary Metabolites	Fiehn	303.143	C12H21N3O6
citric acid	311	Primary Metabolites	Fiehn	192.027	C6H8O7
ferulic acid	445858	Primary Metabolites	Fiehn	194.058	C10H10O4
Betaine	247	Complex lipids	Fiehn	117.079	C5H11NO2
glucoheptonic acid	25588	Primary Metabolites	Fiehn	226.069	C7H14O8
ACETOPHENONE	7410	Primary Metabolites	Fiehn	120.058	C8H8O
benzamide	2331	Primary Metabolites	Fiehn	121.053	C7H7NO
SM(d18:1/14:0)	11433862	Complex lipids	Fiehn	674.536	C37H75N2O6P

Compound Name	PubChem CID	Platform	Lab	Exact Mass	Formula
β-Sitosterol	222284	Primary Metabolites	Fiehn	414.386	C29H50O
gly-pro (N-Glycyl-L-Proline)	79101	Primary Metabolites	Fiehn	172.085	C7H12N2O3
L-valine	6287	Primary Metabolites	Fiehn	117.079	C5H11NO2
valine	1182	Primary Metabolites	Fiehn	117.079	C5H11NO2
trehalose-6-phosphate	122336	Primary Metabolites	Fiehn	422.083	C12H23O14P
DL-2-amino-3-phosphonopropionic acid	177120	Primary Metabolites	Fiehn	169.014	C3H8NO5P
N-carbamyl-L-glutamic acid	3679006	Primary Metabolites	Fiehn	190.059	C6H10N2O5
pyruvic acid	1060	Primary Metabolites	Fiehn	88.016	C3H4O3
5-hydroxytryptophan	144	Primary Metabolites	Fiehn	220.085	C11H12N2O3
5-hydroxy-L-tryptophan	439280	Primary Metabolites	Fiehn	220.085	C11H12N2O3
uric acid	1175	Primary Metabolites	Fiehn	168.028	C5H4N4O3
L-cystine	67678	Primary Metabolites	Fiehn	240.024	C6H12N2O4S2
LysoPC(16:1(9Z))	24779461	Complex lipids	Fiehn	493.317	C24H48NO7P
4-androsten-7-alpha-ol-3,17-dione (7-Hydroxy-4-androstene-3,17-dione)	65542	Primary Metabolites	Fiehn	302.188	C19H26O3
anandamide	5281969	Primary Metabolites	Fiehn	347.282	C22H37NO2
3-Hydroxynorvaline (DL-ß-Hydroxynorvaline)	65097	Primary Metabolites	Fiehn	133.074	C5H11NO3
guaiacol	460	Primary Metabolites	Fiehn	124.052	C7H8O2
2-Hydroxyquinoline	6038	Primary Metabolites	Fiehn	145.053	C9H7NO

Compound Name	PubChem CID	Platform	Lab	Exact Mass	Formula
L-4-Hydroxyphenylglycine	36143	Primary Metabolites	Fiehn	167.058	C8H9NO3
N-Acetyl-beta-alanine	76406	Primary Metabolites	Fiehn	131.058	C5H9NO3
5,6 - dimethylbenzimidazole	675	Primary Metabolites	Fiehn	146.084	C9H10N2
PE(P-16:0/18:2(9Z,12Z))	52925127	Complex lipids	Fiehn	699.52	C39H74NO7P
2-Hydroxybiphenyl	7017	Primary Metabolites	Fiehn	170.073	C12H10O
adenosine-3'-monophosphate (3'-adenylic acid)	41211	Primary Metabolites	Fiehn	347.063	C10H14N5O7P
gallic acid	370	Primary Metabolites	Fiehn	170.022	C7H6O5
urocanic acid	1178	Primary Metabolites	Fiehn	138.043	C6H6N2O2
15-Keto-prostaglandin F2alpha	5280887	Primary Metabolites	Fiehn	352.225	C20H32O5
acetylisatin (N-Acetylisatin)	11321	Primary Metabolites	Fiehn	189.043	C10H7NO3
Dodecanol	8193	Primary Metabolites	Fiehn	186.198	C12H26O
xanthine	1188	Primary Metabolites	Fiehn	152.033	C5H4N4O2
L-norleucine	21236	Primary Metabolites	Fiehn	131.095	C6H13NO2
2-phenylacetamide	7680	Primary Metabolites	Fiehn	135.068	C8H9NO
6-methylprevitamin D	5283772	Primary Metabolites	Fiehn	398.355	C28H46O
resveratrol	445154	Primary Metabolites	Fiehn	228.079	C14H12O3
itaconic acid	811	Primary Metabolites	Fiehn	130.027	C5H6O4
Bis(2-hydroxypropyl)amine	8086	Primary Metabolites	Fiehn	133.11	C6H15NO2

Compound Name	PubChem CID	Platform	Lab	Exact Mass	Formula
pyridoxine	1054	Primary Metabolites	Fiehn	169.074	C8H11NO3
sinapyl alcohol	5280507	Primary Metabolites	Fiehn	210.089	C11H14O4
Tryptophol	10685	Primary Metabolites	Fiehn	161.084	C10H11NO
dihydrolanosterol	440560	Primary Metabolites	Fiehn	428.402	C30H52O
20alpha-Hydroxycholesterol (5-CHOLESTEN-3β,20α-DIOL)	121935	Primary Metabolites	Fiehn	402.35	C27H46O2
cortisone	222786	Primary Metabolites	Fiehn	360.194	C21H28O5
thymol	6989	Primary Metabolites	Fiehn	150.104	C10H14O
4-acetylbutyric acid	18407	Primary Metabolites	Fiehn	130.063	C6H10O3
21-hydroxypregnenolone	440705	Primary Metabolites	Fiehn	332.235	C21H32O3
1,5-Anhydroglucitol	64960	Primary Metabolites	Fiehn	164.068	C6H12O5
2-Amino-2-norbornanecarboxylic acid	115288	Primary Metabolites	Fiehn	155.095	C8H13NO2
purine riboside (Purine-9-D-ribofuranoside)	68368	Primary Metabolites	Fiehn	252.086	C10H12N4O4
L-serine	5951	Primary Metabolites	Fiehn	105.043	C3H7NO3
serine	617	Primary Metabolites	Fiehn	105.043	C3H7NO3
DL-3,4-dihydroxyphenyl glycol	91528	Primary Metabolites	Fiehn	170.058	C8H10O4
DL-Anabasine	2181	Primary Metabolites	Fiehn	162.116	C10H14N2
oxalic acid	971	Primary Metabolites	Fiehn	89.995	C2H2O4
testosterone	6013	Primary Metabolites	Fiehn	288.209	C19H28O2

Compound Name	PubChem CID	Platform	Lab	Exact Mass	Formula
N-acetyl-5-hydroxytryptamine	903	Primary Metabolites	Fiehn	218.106	C12H14N2O2
vanillin	1183	Primary Metabolites	Fiehn	152.047	C8H8O3
PE(18:1(9Z)/18:1(9Z))	9546757	Complex lipids	Fiehn	743.546	C41H78NO8P
tyrosine methyl ester	70652	Primary Metabolites	Fiehn	195.09	C10H13NO3
2-Deoxyuridine	13712	Primary Metabolites	Fiehn	228.075	C9H12N2O5
piperine	638024	Primary Metabolites	Fiehn	285.136	C17H19NO3
prostaglandin A2	5280880	Primary Metabolites	Fiehn	334.214	C20H30O4
nornicotine ((+/-)-Nornicotine)	412	Primary Metabolites	Fiehn	148.1	C9H12N2
SM(d18:1/18:1(9Z))	6443882	Complex lipids	Fiehn	728.583	C41H81N2O6P
phosphoric acid (ortho-Phosphoric acid)	1004	Primary Metabolites	Fiehn	97.977	H3O4P
iminodiacetic acid	8897	Primary Metabolites	Fiehn	133.038	C4H7NO4
PC(22:6(4Z,7Z,10Z,13Z,16Z,19Z)/18:1(9Z))	52923693	Complex lipids	Fiehn	831.578	C48H82NO8P
(−)-perillyl alcohol	369312	Primary Metabolites	Fiehn	152.12	C10H16O
phenyl-beta-glucopyranoside (Phenyl beta-D-glucopyranoside)	65080	Primary Metabolites	Fiehn	256.095	C12H16O6
L-menthone	26447	Primary Metabolites	Fiehn	154.136	C10H18O
atrazin-2-hydroxy	16553	Primary Metabolites	Fiehn	197.128	C8H15N5O
DEHYDROABIETIC ACID	94391	Primary Metabolites	Fiehn	300.209	C20H28O2
salicin	5145	Primary Metabolites	Fiehn	286.105	C13H18O7

Compound Name	PubChem CID	Platform	Lab	Exact Mass	Formula
erythrose 4-phosphate	122357	Primary Metabolites	Fiehn	200.009	C4H9O7P
(+)-6-aminopenicillanic acid	11082	Primary Metabolites	Fiehn	216.057	C8H12N2O3S
4-hydroxycinnamic acid (D-erythro-sphingosine) (p-Coumaric acid)	637542	Primary Metabolites	Fiehn	164.047	C9H8O3
pyridoxal 5′-phosphate	1051	Primary Metabolites	Fiehn	247.025	C8H10NO6P
fluorene	6853	Primary Metabolites	Fiehn	166.078	C13H10
cycloleucine	2901	Primary Metabolites	Fiehn	129.079	C6H11NO2
3-(4-hydroxyphenyl)propionic acid	10394	Primary Metabolites	Fiehn	166.063	C9H10O3
mandelonitrile	10758	Primary Metabolites	Fiehn	133.053	C8H7NO
3-hydroxy-3-methylglutaric acid (dicrotalic acid)	1662	Primary Metabolites	Fiehn	162.053	C6H10O5
Aminooxyacetic acid	286	Primary Metabolites	Fiehn	91.027	C2H5NO3
PE(P-16:0/20:2(11Z,14Z))	52925059	Complex lipids	Fiehn	727.552	C41H78NO7P
Allantoic acid	203	Primary Metabolites	Fiehn	176.055	C4H8N4O4
octanal (Octyl Aldehyde)	454	Primary Metabolites	Fiehn	128.12	C8H16O
5β-ANDROSTAN-17β-OL-3-ONE (dihydrotestosterone)	10635	Primary Metabolites	Fiehn	290.225	C19H30O2
2-Amino-3-methyl-1-butanol (Levoglucosan)	79019	Primary Metabolites	Fiehn	103.1	C5H13NO
guanosine	6802	Primary Metabolites	Fiehn	283.092	C10H13N5O5
(+) -4-cholesten-3-one	91477	Primary Metabolites	Fiehn	384.339	C27H44O
4-Cholesten-3-one	440	Primary Metabolites	Fiehn	384.339	C27H44O

Compound Name	PubChem CID	Platform	Lab	Exact Mass	Formula
isocitric acid	1198	Primary Metabolites	Fiehn	192.027	C6H8O7
L-pyroglutamic acid (oxoproline)	7405	Primary Metabolites	Fiehn	129.043	C5H7NO3
arginine (L-Arginine)	6322	Primary Metabolites	Fiehn	174.112	C6H14N4O2
malonic acid	867	Primary Metabolites	Fiehn	104.011	C3H4O4
2,4-diaminobutyric acid	470	Primary Metabolites	Fiehn	118.074	C4H10N2O2
biuret	7913	Primary Metabolites	Fiehn	103.038	C2H5N3O2
4-isopropylbenzyl alcohol (cuminic alcohol)	325	Primary Metabolites	Fiehn	150.104	C10H14O
1,2-CYCLOHEXANEDIONE	13006	Primary Metabolites	Fiehn	112.052	C6H8O2
orcinol (5-METHYLRESORCINOL)	10436	Primary Metabolites	Fiehn	124.052	C7H8O2
adenosine	60961	Primary Metabolites	Fiehn	267.097	C10H13N5O4
5,6-dihydrouracil	649	Primary Metabolites	Fiehn	114.043	C4H6N2O2
3-hydroxybenzyl alcohol	102	Primary Metabolites	Fiehn	124.052	C7H8O2
CORTICOSTERONE	5753	Primary Metabolites	Fiehn	346.214	C21H30O4
L-proline	145742	Primary Metabolites	Fiehn	115.063	C5H9NO2
o-Hydroxyhippuric acid	10253	Primary Metabolites	Fiehn	195.053	C9H9NO4
4-hydroxy-6-methyl-2-pyrone	12663	Primary Metabolites	Fiehn	126.032	C6H6O3
Cytosine	597	Primary Metabolites	Fiehn	111.043	C4H5N3O
diethyl oxalpropionate (2,8-Dihydroxyquinoline)	97750	Primary Metabolites	Fiehn	202.084	C9H14O5

Compound Name	PubChem CID	Platform	Lab	Exact Mass	Formula
N-acetyl-L-aspartic acid	65065	Primary Metabolites	Fiehn	175.048	C6H9NO5
DL-dihydrosphingosine	3126	Primary Metabolites	Fiehn	301.298	C18H39NO2
D-Tyrosine	71098	Primary Metabolites	Fiehn	181.074	C9H11NO3
L-tyrosine	6057	Primary Metabolites	Fiehn	181.074	C9H11NO3
2-amino-3-(4-hydroxyphenyl)propanoic acid (DL-Tyrosine)	1153	Primary Metabolites	Fiehn	181.074	C9H11NO3
N-Acetyl-D-glucosamine	24139	Primary Metabolites	Fiehn	221.09	C8H15NO6
N-acetyl-D-mannosamine	13201636	Primary Metabolites	Fiehn	221.09	C8H15NO6
N-Acetyl-D-galactosamine	35717	Primary Metabolites	Fiehn	221.09	C8H15NO6
linoleic acid	5280450	Primary Metabolites	Fiehn	280.24	C18H32O2
alpha-Aminoadipic acid	469	Primary Metabolites	Fiehn	161.069	C6H11NO4
5-cholesten-3-beta-7-alpha-diol (7-alpha-Hydroxycholesterol)(7β-Hydroxycholesterol)	107722	Primary Metabolites	Fiehn	402.35	C27H46O2
cyclohexylamine (cyclohexanamine)	7965	Primary Metabolites	Fiehn	99.105	C6H13N
1-Aminocyclopropanecarboxylic acid	535	Primary Metabolites	Fiehn	101.048	C4H7NO2
aniline	6115	Primary Metabolites	Fiehn	93.058	C6H7N
cis-sinapinic acid	1549091	Primary Metabolites	Fiehn	224.068	C11H12O5
3,5-dimethoxy-4-hydroxycinnamic acid (sinapinic acid)	637775	Primary Metabolites	Fiehn	224.068	C11H12O5
glycerol	753	Primary Metabolites	Fiehn	92.047	C3H8O3
MALEIMIDE	10935	Primary	Fiehn	97.016	C4H3NO2

Compound Name	PubChem CID	Platform	Lab	Exact Mass	Formula
		Metabolites			
coprostan-3-one	85881	Primary Metabolites	Fiehn	386.355	C27H46O
5alpha-cholestan-3-one	92128	Primary Metabolites	Fiehn	386.355	C27H46O
spermine	1103	Primary Metabolites	Fiehn	202.216	C10H26N4
(+)-catechin	9064	Primary Metabolites	Fiehn	290.079	C15H14O6
(-)-epicatechin	72276	Primary Metabolites	Fiehn	290.079	C15H14O6
trans-cyclohexane-1,2-diol	92886	Primary Metabolites	Fiehn	116.084	C6H12O2
cyclohexane-1,2-diol	13601	Primary Metabolites	Fiehn	116.084	C6H12O2
3-methylcatechol	340	Primary Metabolites	Fiehn	124.052	C7H8O2
(S)-Carnitine	2724480	Primary Metabolites	Fiehn	161.105	C7H15NO3
Carnitine	288	Primary Metabolites	Fiehn	161.105	C7H15NO3
L-carnitine	10917	Primary Metabolites	Fiehn	161.105	C7H15NO3
Carnitine	10917	Complex lipids	Fiehn	161.105	C7H15NO3
D-(+)-Gluconic acid δ-lactone	7027	Primary Metabolites	Fiehn	178.048	C6H10O6
3-(1-Pyrazolyl)-L-alanine	151491	Primary Metabolites	Fiehn	155.069	C6H9N3O2
PC(20:4(5Z,8Z,11Z,14Z)/18:1(9Z))	52923293	Complex lipids	Fiehn	807.578	C46H82NO8P
sucrose-6-phosphate	439762	Primary Metabolites	Fiehn	422.083	C12H23O14P
4-aminophenol	403	Primary Metabolites	Fiehn	109.053	C6H7NO

Compound Name	PubChem CID	Platform	Lab	Exact Mass	Formula
trans-4-hydroxy-L-proline	5810	Primary Metabolites	Fiehn	131.058	C5H9NO3
2-hydroxycinnamic acid (o-coumaric acid)	637540	Primary Metabolites	Fiehn	164.047	C9H8O3
cis-o-coumarinic acid	5280841	Primary Metabolites	Fiehn	164.047	C9H8O3
4-hydroxyquinoline	69141	Primary Metabolites	Fiehn	145.053	C9H7NO
D-(+)-Fucose	94270	Primary Metabolites	Fiehn	164.068	C6H12O5
L-(−)-fucose	3034656	Primary Metabolites	Fiehn	164.068	C6H12O5
tetracosane	12592	Primary Metabolites	Fiehn	338.391	C24H50
lauric acid	3893	Primary Metabolites	Fiehn	200.178	C12H24O2
ribose-5-phosphate	77982	Primary Metabolites	Fiehn	230.019	C5H11O8P
ALDOSTERONE	5839	Primary Metabolites	Fiehn	360.194	C21H28O5
estriol	5756	Primary Metabolites	Fiehn	288.173	C18H24O3
PC(20:4(5Z,8Z,11Z,14Z)/16:0)	24779073	Complex lipids	Fiehn	781.562	C44H80NO8P
nicotinic acid	938	Primary Metabolites	Fiehn	123.032	C6H5NO2
palatinose	439559	Primary Metabolites	Fiehn	342.116	C12H22O11
11-beta-prostaglandin-F-2-alpha	5280886	Primary Metabolites	Fiehn	354.241	C20H34O5
orotic acid	967	Primary Metabolites	Fiehn	156.017	C5H4N2O4
ribose	5311110	Primary Metabolites	Fiehn	150.053	C5H10O5
D-lyxose	65550	Primary Metabolites	Fiehn	150.053	C5H10O5

Compound Name	PubChem CID	Platform	Lab	Exact Mass	Formula
D-(+)-Xylose	644160	Primary Metabolites	Fiehn	150.053	C5H10O5
N-formyl-L-methionine	439750	Primary Metabolites	Fiehn	177.046	C6H11NO3S
LysoPE(18:1n9)	9547071	Complex lipids	Fiehn	479.301	C23H46NO7P
sophorose	92797	Primary Metabolites	Fiehn	342.116	C12H22O11
3,4-dihydroxycinnamic acid	689043	Primary Metabolites	Fiehn	180.042	C9H8O4
caffeic acid	1549111	Primary Metabolites	Fiehn	180.042	C9H8O4
sulfuric acid	1118	Primary Metabolites	Fiehn	97.967	H2O4S
phloroglucinol	359	Primary Metabolites	Fiehn	126.032	C6H6O3
DL-3-aminoisobutyric acid	64956	Primary Metabolites	Fiehn	103.063	C4H9NO2
methionine sulfoxide	847	Primary Metabolites	Fiehn	165.046	C5H11NO3S
L-methionine sulfoxide	158980	Primary Metabolites	Fiehn	165.046	C5H11NO3S
icasonic acid methyl ester (methyl icosanoate)	14259	Primary Metabolites	Fiehn	326.318	C21H42O2
androsterone	5879	Primary Metabolites	Fiehn	290.225	C19H30O2
3-methyl-2-oxobutanoic acid (2-keto-isovalerate)	49	Primary Metabolites	Fiehn	116.047	C5H8O3
PE(16:1(9Z)/16:0)	52924223	Complex lipids	Fiehn	689.5	C37H72NO8P
1-Monopalmitin	14900	Primary Metabolites	Fiehn	330.277	C19H38O4
hippuric acid	464	Primary Metabolites	Fiehn	179.058	C9H9NO3
hydroquinone	785	Primary Metabolites	Fiehn	110.037	C6H6O2

Compound Name	PubChem CID	Platform	Lab	Exact Mass	Formula
stearic acid	5281	Primary Metabolites	Fiehn	284.272	C18H36O2
tryptophan	1148	Primary Metabolites	Fiehn	204.09	C11H12N2O2
L-tryptophan	6305	Primary Metabolites	Fiehn	204.09	C11H12N2O2
PC(18:1(9Z)/16:1(9Z))	24778935	Complex lipids	Fiehn	757.562	C42H80NO8P
PC(20:1(11Z)/18:1(9Z))	52923113	Complex lipids	Fiehn	813.625	C46H88NO8P
4-Hydroxyquinazoline	63112	Primary Metabolites	Fiehn	146.048	C8H6N2O
alanine (DL-Alanine)	602	Primary Metabolites	Fiehn	89.048	C3H7NO2
1-Indanone	6735	Primary Metabolites	Fiehn	132.058	C9H8O
cis-1,2-dihydro-1,2-naphthalenediol ((1R,2S)-cis-1,2-dihydro-1,2-naphthalenediol)	440294	Primary Metabolites	Fiehn	162.068	C10H10O2
N-Oleoyldopamine	5282106	Primary Metabolites	Fiehn	417.324	C26H43NO3
SM(d16:1/18:1)	52931143	Complex lipids	Fiehn	700.552	C39H77N2O6P
ciliatine	339	Primary Metabolites	Fiehn	125.024	C2H8NO3P
Homovanillic acid	1738	Primary Metabolites	Fiehn	182.058	C9H10O4
docosanoic acid, methyl ester	13584	Primary Metabolites	Fiehn	354.35	C23H46O2
3-(3-hydroxyphenyl)propionic acid	91	Primary Metabolites	Fiehn	166.063	C9H10O3
melezitose (D-(+)-melezitose)	92817	Primary Metabolites	Fiehn	504.169	C18H32O16
o-cresol	335	Primary Metabolites	Fiehn	108.058	C7H8O
Butyryl carnitine	213144	Complex	Fiehn	231.147	C11H21NO4

Compound Name	PubChem CID	Platform	Lab	Exact Mass	Formula
		lipids			
xanthotoxin	4114	Primary Metabolites	Fiehn	216.042	C12H8O4
Cholestan-3beta-ol	6665	Primary Metabolites	Fiehn	388.371	C27H48O
cholecalciferol	5280795	Primary Metabolites	Fiehn	384.339	C27H44O
Lignoceric acid	11197	Primary Metabolites	Fiehn	368.365	C24H48O2
1,3,5(10)-estratrien-3,6- beta-17-beta-triol	440168	Primary Metabolites	Fiehn	288.173	C18H24O3
5beta-androstane-3,17-dione	440114	Primary Metabolites	Fiehn	288.209	C19H28O2
5β-ANDROSTAN-3,17-DIONE	222865	Primary Metabolites	Fiehn	288.209	C19H28O2
D-Glyceric acid	439194	Primary Metabolites	Fiehn	106.027	C3H6O4
Acetylcarnitine	7045767	Complex lipids	Fiehn	203.116	C9H17NO4
N-acetyl-L-glutamic acid	70914	Primary Metabolites	Fiehn	189.064	C7H11NO5
alizarin	6293	Primary Metabolites	Fiehn	240.042	C14H8O4
4-hydroxybenzaldehyde	126	Primary Metabolites	Fiehn	122.037	C7H6O2
3,4-dihydroxymandelic acid	85782	Primary Metabolites	Fiehn	184.037	C8H8O5
Galactonic acid	128869	Primary Metabolites	Fiehn	196.058	C6H12O7
gluconic acid	604	Primary Metabolites	Fiehn	196.058	C6H12O7
2'-Deoxycytidine 5'-triphosphate	65091	Primary Metabolites	Fiehn	466.99	C9H16N3O13P3
L-citrulline	9750	Primary Metabolites	Fiehn	175.096	C6H13N3O3

Compound Name	PubChem CID	Platform	Lab	Exact Mass	Formula
tetrahydrocorticosterone	65553	Primary Metabolites	Fiehn	350.246	C21H34O4
1-Methylhydantoin	69217	Primary Metabolites	Fiehn	114.043	C4H6N2O2
5-beta-cholestan-3-alpha-7-alpha-12-alpha-triol (3,7,12-Trihydroxycoprostane)	160520	Primary Metabolites	Fiehn	420.36	C27H48O3
progesterone	5994	Primary Metabolites	Fiehn	314.225	C21H30O2
lyxonic acid, 1,4-lactone, 3TMS	6427169	Primary Metabolites	Fiehn	364.156	C14H32O5Si3
hyoscyamine (atropine)	3661	Primary Metabolites	Fiehn	289.168	C17H23NO3
phytanic acid	26840	Primary Metabolites	Fiehn	312.303	C20H40O2
m-cresol	342	Primary Metabolites	Fiehn	108.058	C7H8O
3-isopropylmalate	5462261	Primary Metabolites	Fiehn	176.068	C7H12O5
2-isopropylmalate	36	Primary Metabolites	Fiehn	176.068	C7H12O5
tartronic acid	45	Primary Metabolites	Fiehn	120.006	C3H4O5
scopoletin	5280460	Primary Metabolites	Fiehn	192.042	C10H8O4
leucine	857	Primary Metabolites	Fiehn	131.095	C6H13NO2
L-leucine	6106	Primary Metabolites	Fiehn	131.095	C6H13NO2
guanosine-5'-monophosphate	6804	Primary Metabolites	Fiehn	363.058	C10H14N5O8P
PC(18:1(9Z)/16:0)	24778933	Complex lipids	Fiehn	759.578	C42H82NO8P
3-Indolepyruvic acid	803	Primary Metabolites	Fiehn	203.058	C11H9NO3
abietic acid	10569	Primary Metabolites	Fiehn	302.225	C20H30O2

Compound Name	PubChem CID	Platform	Lab	Exact Mass	Formula
PC(18:2(9Z,12Z)/18:1(9Z))	52922727	Complex lipids	Fiehn	783.578	C44H82NO8P
chrysin	5281607	Primary Metabolites	Fiehn	254.058	C15H10O4
turanose	5460935	Primary Metabolites	Fiehn	342.116	C12H22O11
SM(d18:1/16:0)	9939941	Complex lipids	Fiehn	702.568	C39H79N2O6P
thymine	1135	Primary Metabolites	Fiehn	126.043	C5H6N2O2
L-glutathione	124886	Primary Metabolites	Fiehn	307.084	C10H17N3O6S
Monoolein (1-Oleoyl-rac-glycerol)	5283468	Primary Metabolites	Fiehn	356.293	C21H40O4
adrenosterone	223997	Primary Metabolites	Fiehn	300.173	C19H24O3
palmitoleic acid	445638	Primary Metabolites	Fiehn	254.225	C16H30O2
3-indoleacetic acid	802	Primary Metabolites	Fiehn	175.063	C10H9NO2
palatinitol	3034828	Primary Metabolites	Fiehn	344.132	C12H24O11
norepinephrine (noradrenaline)	439260	Primary Metabolites	Fiehn	169.074	C8H11NO3
noradrenaline	951	Primary Metabolites	Fiehn	169.074	C8H11NO3
D-Erythronic acid gamma-Lactone	5325915	Primary Metabolites	Fiehn	118.027	C4H6O4
6-deoxy-D-glucose	441480	Primary Metabolites	Fiehn	164.068	C6H12O5
picolinic acid	1018	Primary Metabolites	Fiehn	123.032	C6H5NO2
1-Hydroxy-2-naphthoic acid	6844	Primary Metabolites	Fiehn	188.047	C11H8O3
4-hydroxybutyrate	10413	Primary Metabolites	Fiehn	104.047	C4H8O3

Compound Name	PubChem CID	Platform	Lab	Exact Mass	Formula
dehydroshikimic acid	80	Primary Metabolites	Fiehn	172.037	C7H8O5
2-FUROIC ACID	6919	Primary Metabolites	Fiehn	112.016	C5H4O3
salicylaldehyde	6998	Primary Metabolites	Fiehn	122.037	C7H6O2
norvaline	65098	Primary Metabolites	Fiehn	117.079	C5H11NO2
PC(18:1(9Z)/18:1(9Z))	10350317	Complex lipids	Fiehn	785.593	C44H84NO8P
D-panthenol	131204	Primary Metabolites	Fiehn	205.131	C9H19NO4
oxamic acid	974	Primary Metabolites	Fiehn	89.011	C2H3NO3
Hydantoin, 5-(4-hydroxybutyl)	95514	Primary Metabolites	Fiehn	172.085	C7H12N2O3
O-phosphocolamine (O-phosphorylethanolamine)	1015	Primary Metabolites	Fiehn	141.019	C2H8NO4P
Acylcarnitine C20:2	n/a	Complex lipids	Fiehn		
N-cyclohexylformamide	13017	Primary Metabolites	Fiehn	127.1	C7H13NO
2-HYDROXYESTRONE	440623	Primary Metabolites	Fiehn	286.157	C18H22O3
1,3,5(10)-ESTRATRIEN-2,3-DIOL-17-ONE	9685	Primary Metabolites	Fiehn	286.157	C18H22O3
4-Hydroxy-4-methyl-2-pentanone	31256	Primary Metabolites	Fiehn	116.084	C6H12O2
2-Amino-3-methoxybenzoic acid	255720	Primary Metabolites	Fiehn	167.058	C8H9NO3
lactamide	94220	Primary Metabolites	Fiehn	89.048	C3H7NO2
glutaraldehyde	3485	Primary Metabolites	Fiehn	100.052	C5H8O2
L-gulonic acid γ-lactone	439373	Primary Metabolites	Fiehn	178.048	C6H10O6

Compound Name	PubChem CID	Platform	Lab	Exact Mass	Formula
myristic acid	11005	Primary Metabolites	Fiehn	228.209	C14H28O2
Acylcarnitine C12:1	n/a	Complex lipids	Fiehn		
dibenzofuran	568	Primary Metabolites	Fiehn	168.058	C12H8O
trans,trans-muconic acid	5356793	Primary Metabolites	Fiehn	142.027	C6H6O4
2-oxobutyrate	58	Primary Metabolites	Fiehn	102.032	C4H6O3
L-ascorbic acid	5785	Primary Metabolites	Fiehn	176.032	C6H8O6
ascorbate	235	Primary Metabolites	Fiehn	176.032	C6H8O6
xanthosine	1189	Primary Metabolites	Fiehn	284.076	C10H12N4O6
2-hydroxypyridine	8871	Primary Metabolites	Fiehn	95.037	C5H5NO
Beta- alanine	239	Primary Metabolites	Fiehn	89.048	C3H7NO2
adrenaline ((-)-Epinephrine)	5816	Primary Metabolites	Fiehn	183.09	C9H13NO3
Adenosine 5'-monophosphate	6083	Primary Metabolites	Fiehn	347.063	C10H14N5O7P
adenosine-5-monophosphate	224	Primary Metabolites	Fiehn	347.063	C10H14N5O7P
2-aminoethanethiol	6058	Primary Metabolites	Fiehn	77.03	C2H7NS
naphthalene	931	Primary Metabolites	Fiehn	128.063	C10H8
Glucosaminic acid	73563	Primary Metabolites	Fiehn	195.074	C6H13NO6
inosine	6021	Primary Metabolites	Fiehn	268.081	C10H12N4O5
cytidine	596	Primary Metabolites	Fiehn	243.086	C9H13N3O5

Compound Name	PubChem CID	Platform	Lab	Exact Mass	Formula
PE(18:1(9Z)/16:1(9Z))	52924899	Complex lipids	Fiehn	715.515	C39H74NO8P
6-methylmercaptopurine	5778	Primary Metabolites	Fiehn	166.031	C6H6N4S
N-(3-aminopropyl)-morpholine	61055	Primary Metabolites	Fiehn	144.126	C7H16N2O
succinate semialdehyde	1112	Primary Metabolites	Fiehn	102.032	C4H6O3
PC(16:0/14:0)	24778679	Complex lipids	Fiehn	705.531	C38H76NO8P
carbazole	6854	Primary Metabolites	Fiehn	167.073	C12H9N
L-homoserine	12647	Primary Metabolites	Fiehn	119.058	C4H9NO3
behenic acid	8215	Primary Metabolites	Fiehn	340.334	C22H44O2
(R)-(-)-carvone	7439	Primary Metabolites	Fiehn	150.104	C10H14O
2-amino-1-phenylethanol	1000	Primary Metabolites	Fiehn	137.084	C8H11NO
2-INDANONE	11983	Primary Metabolites	Fiehn	132.058	C9H8O
D-threitol	169019	Primary Metabolites	Fiehn	122.058	C4H10O4
alpha-Ecdysone	19212	Primary Metabolites	Fiehn	464.314	C27H44O6
2-Monoolein (2-oleoylglycerol)	5319879	Primary Metabolites	Fiehn	356.293	C21H40O4
dodecanoic acid methyl ester (methyl dodecanoate)	8139	Primary Metabolites	Fiehn	214.193	C13H26O2
8-aminocaprylic acid	66085	Primary Metabolites	Fiehn	159.126	C8H17NO2
PE(P-18:0/20:4)	9547058	Complex lipids	Fiehn	751.552	C43H78NO7P
N-carbobenzyloxy-L-leucine	74840	Primary Metabolites	Fiehn	265.131	C14H19NO4

Compound Name	PubChem CID	Platform	Lab	Exact Mass	Formula
O-phospho-L-threonine	3246323	Primary Metabolites	Fiehn	199.025	C4H10NO6P
O-phosphonothreonine	1016	Primary Metabolites	Fiehn	199.025	C4H10NO6P
PC(20:3(8Z,11Z,14Z)/18:0)	52923231	Complex lipids	Fiehn	811.609	C46H86NO8P
phenaceturic acid	68144	Primary Metabolites	Fiehn	193.074	C10H11NO3
PE(P-16:0/20:4(5Z,8Z,11Z,14Z))	52925126	Complex lipids	Fiehn	723.52	C41H74NO7P
2-amino-2-methyl-1,3-propanediol	1531	Primary Metabolites	Fiehn	105.079	C4H11NO2
lumazine	10250	Primary Metabolites	Fiehn	164.033	C6H4N4O2
4-acetamidobutyric acid	18189	Primary Metabolites	Fiehn	145.074	C6H11NO3
1-Kestose	440080	Primary Metabolites	Fiehn	504.169	C18H32O16
Monostearin (1-Stearoyl-rac-glycerol)	24699	Primary Metabolites	Fiehn	358.308	C21H42O4
2-Keto-L-gulonic acid	102424	Primary Metabolites	Fiehn	194.043	C6H10O7
galactinol	439451	Primary Metabolites	Fiehn	342.116	C12H22O11
D-glucose-6-phosphate	439958	Primary Metabolites	Fiehn	260.03	C6H13O9P
PE(20:4(5Z,8Z,11Z,14Z)/18:1(9Z))	52924645	Complex lipids	Fiehn	765.531	C43H76NO8P
phloretin	4788	Primary Metabolites	Fiehn	274.084	C15H14O5
flavone	10680	Primary Metabolites	Fiehn	222.068	C15H10O2
L-dithiothreitol	439196	Primary Metabolites	Fiehn	154.012	C4H10O2S2
Dithioerythritol	19001	Primary Metabolites	Fiehn	154.012	C4H10O2S2

**Compound Name**	**PubChem CID**	**Platform**	**Lab**	**Exact Mass**	**Formula**
1,4-dithioerythritol	439352	Primary Metabolites	Fiehn	154.012	C4H10O2S2
hexacosanoic acid methyl ester (METHYL HEXACOSANOATE)	22048	Primary Metabolites	Fiehn	410.412	C27H54O2
arachidic acid	10467	Primary Metabolites	Fiehn	312.303	C20H40O2
dihydrocoumarin	660	Primary Metabolites	Fiehn	148.052	C9H8O2
1,4-Dihydroxy-2-naphthoic acid	671	Primary Metabolites	Fiehn	204.042	C11H8O4
3-phenyllactic acid	3848	Primary Metabolites	Fiehn	166.063	C9H10O3
Epiestradiol	68570	Primary Metabolites	Fiehn	272.178	C18H24O2
1,3,5(10)-ESTRATRIEN-3,17α-DIOL (estradiol)	5757	Primary Metabolites	Fiehn	272.178	C18H24O2
maltitol	16217663	Primary Metabolites	Fiehn	344.132	C12H24O11
lactitol	91476	Primary Metabolites	Fiehn	344.132	C12H24O11
Cellobiotol	160514	Primary Metabolites	Fiehn	344.132	C12H24O11
4-quinolinecarboxylic acid	10243	Primary Metabolites	Fiehn	173.048	C10H7NO2
2-deoxy-D-glucose (2-Deoxy-D-galactose)	108223	Primary Metabolites	Fiehn	164.068	C6H12O5
hydroxyurea	3657	Primary Metabolites	Fiehn	76.027	CH4N2O2
guanidinosuccinic acid	97856	Primary Metabolites	Fiehn	175.059	C5H9N3O4
flavin adenine dinucleotide	643975	Primary Metabolites	Fiehn	785.157	C27H33N9O15P2
LysoPC(14:0)	460604	Complex lipids	Fiehn	467.301	C22H46NO7P
fumaric acid	444972	Primary Metabolites	Fiehn	116.011	C4H4O4

Compound Name	PubChem CID	Platform	Lab	Exact Mass	Formula
maleic acid	444266	Primary Metabolites	Fiehn	116.011	C4H4O4
acetyl-L-serine (O-acetylserine)	189	Primary Metabolites	Fiehn	147.053	C5H9NO4
cinnamic acid	444539	Primary Metabolites	Fiehn	148.052	C9H8O2
benzylamine	7504	Primary Metabolites	Fiehn	107.073	C7H9N
Cortexolone (Reichstein´s Substances)	9050	Primary Metabolites	Fiehn	346.214	C21H30O4
3-hydroxybutyric acid	441	Primary Metabolites	Fiehn	104.047	C4H8O3
(S)-3-Hydroxybutyric acid	94318	Primary Metabolites	Fiehn	104.047	C4H8O3
2-METHOXYESTRONE [1,3,5(10)-ESTRATRIEN-2,3-DIOL-17-ONE2-METHYLETHER]	440624	Primary Metabolites	Fiehn	300.173	C19H24O3
glutamic acid	611	Primary Metabolites	Fiehn	147.053	C5H9NO4
L-Glutamic acid	33032	Primary Metabolites	Fiehn	147.053	C5H9NO4
Methoxamedrine	6082	Primary Metabolites	Fiehn	211.121	C11H17NO3
3,4-dimethoxybenzaldehyde	8419	Primary Metabolites	Fiehn	166.063	C9H10O3
3-hydroxyanthranilic acid	86	Primary Metabolites	Fiehn	153.043	C7H7NO3
vanillic acid (4-hydroxy-3-methoxybenzoic acid)	8468	Primary Metabolites	Fiehn	168.042	C8H8O4
pimelic acid	385	Primary Metabolites	Fiehn	160.074	C7H12O4
PHENYLACETIC ACID	999	Primary Metabolites	Fiehn	136.052	C8H8O2
adipic acid	196	Primary Metabolites	Fiehn	146.058	C6H10O4
3-Methylamino-1,2-propanediol	2734122	Primary	Fiehn	105.079	C4H11NO2

Compound Name	PubChem CID	Platform	Lab	Exact Mass	Formula
		Metabolites			
benzoic acid	243	Primary Metabolites	Fiehn	122.037	C7H6O2
pentadecanoic acid	13849	Primary Metabolites	Fiehn	242.225	C15H30O2
pyrogallol	1057	Primary Metabolites	Fiehn	126.032	C6H6O3
galactose	6036	Primary Metabolites	Fiehn	180.063	C6H12O6
pyrrole -2-carboxylic acid	12473	Primary Metabolites	Fiehn	111.032	C5H5NO2
malonamide	7911	Primary Metabolites	Fiehn	102.043	C3H6N2O2
(-)-Dihydrocarveol	37894	Primary Metabolites	Fiehn	154.136	C10H18O
4-androsten-11-beta-ol-3,17-dione	94141	Primary Metabolites	Fiehn	302.188	C19H26O3
3-(3,4-Dihydroxyphenyl)-L-alanine [L-DOPA]	6047	Primary Metabolites	Fiehn	197.069	C9H11NO4
L-3,4-Dihydroxyphenylalanine	836	Primary Metabolites	Fiehn	197.069	C9H11NO4
citral	638011	Primary Metabolites	Fiehn	152.12	C10H16O
Linoleic acid methyl ester	5284421	Primary Metabolites	Fiehn	294.256	C19H34O2
5,7-dihydroxy-3-(4-methoxyphenyl)chromen-4-one (Biochanin A = 5,7-Dihydroxy-4'-Methoxyisoflavone)	5280373	Primary Metabolites	Fiehn	284.068	C16H12O5
5′-deoxy-5′-(methylthio)adenosine (5'-methylthioadenosine)	439176	Primary Metabolites	Fiehn	297.09	C11H15N5O3S
benzyl alcohol	244	Primary Metabolites	Fiehn	108.058	C7H8O
PE(P-16:0/22:6)	5283497	Complex lipids	Fiehn	747.52	C43H74NO7P
N-methylanthranilic acid	67069	Primary Metabolites	Fiehn	151.063	C8H9NO2

Compound Name	PubChem CID	Platform	Lab	Exact Mass	Formula
Sphingosine	5280335	Complex lipids	Fiehn	299.282	C18H37NO2
caprylic acid	379	Primary Metabolites	Fiehn	144.115	C8H16O2
N-acetyl-L-leucine	70912	Primary Metabolites	Fiehn	173.105	C8H15NO3
gentisic acid	3469	Primary Metabolites	Fiehn	154.027	C7H6O4
paraoxon ethyl	9395	Primary Metabolites	Fiehn	275.056	C10H14NO6P
3,6-Anhydro-D-galactose	16069996	Primary Metabolites	Fiehn	162.053	C6H10O5
Prostaglandin E2	158	Primary Metabolites	Fiehn	352.225	C20H32O5
1,3-diaminopropane	428	Primary Metabolites	Fiehn	74.084	C3H10N2
citramalic acid	439766	Primary Metabolites	Fiehn	148.037	C5H8O5
3-indolelactic acid (Indolelactate)	92904	Primary Metabolites	Fiehn	205.074	C11H11NO3
4-Androsten-19-ol-3,17-dione	252379	Primary Metabolites	Fiehn	302.188	C19H26O3
maltotriitol	3625615	Primary Metabolites	Fiehn	506.185	C18H34O16
alpha-santonin	221071	Primary Metabolites	Fiehn	246.126	C15H18O3
3-METHYLGLUTARIC ACID	12284	Primary Metabolites	Fiehn	146.058	C6H10O4
4-nitrocatechol	3505109	Primary Metabolites	Fiehn	155.022	C6H5NO4
Nω-acetylhistamine (N-omega-Acetylhistamine)	69602	Primary Metabolites	Fiehn	153.09	C7H11N3O
N-Methyl-L-glutamic acid	439377	Primary Metabolites	Fiehn	161.069	C6H11NO4
acetol	8299	Primary Metabolites	Fiehn	74.037	C3H6O2

Compound Name	PubChem CID	Platform	Lab	Exact Mass	Formula
Cerotinic acid	10469	Primary Metabolites	Fiehn	396.397	C26H52O2
hydrocinnamic acid	107	Primary Metabolites	Fiehn	150.068	C9H10O2
epigallocatechin	72277	Primary Metabolites	Fiehn	306.074	C15H14O7
5-alpha-pregnan-3,20-dione (5-alpha-Dihydroprogesterone; 5α-PREGNAN-3,20-DIONE)	92810	Primary Metabolites	Fiehn	316.24	C21H32O2
phthalic acid	1017	Primary Metabolites	Fiehn	166.027	C8H6O4
2-ketocaproic acid	159664	Primary Metabolites	Fiehn	130.063	C6H10O3
taurine	1123	Primary Metabolites	Fiehn	125.015	C2H7NO3S
Acylcarnitine C16:0	16902	Complex lipids	Fiehn	399.335	C23H45NO4
pyrophosphate	1023	Primary Metabolites	Fiehn	177.943	H4O7P2
D-Fructose 1,6-bisphosphate	84951	Primary Metabolites	Fiehn	339.996	C6H14O12P2
4-hydroxyphenylacetic acid	127	Primary Metabolites	Fiehn	152.047	C8H8O3
urea	1176	Primary Metabolites	Fiehn	60.032	CH4N2O
tetracosanoic acid methyl ester (Methyl tetracosanoate)	75546	Primary Metabolites	Fiehn	382.381	C25H50O2
L-Cysteine	5862	Primary Metabolites	Fiehn	121.02	C3H7NO2S
L-cysteine	594	Primary Metabolites	Fiehn	121.02	C3H7NO2S
2-piperidone (5-aminovaleric acid lactam)	12665	Primary Metabolites	Fiehn	99.068	C5H9NO
Glutaconic acid	5280498	Primary Metabolites	Fiehn	130.027	C5H6O4
L-cysteic acid	72886	Primary	Fiehn	169.004	C3H7NO5S

Compound Name	PubChem CID	Platform	Lab	Exact Mass	Formula
		Metabolites			
4-nitrophenyl phosphate	378	Primary Metabolites	Fiehn	218.993	C6H6NO6P
PC(22:6(4Z,7Z,10Z,13Z,16Z,19Z)/16:0)	24779131	Complex lipids	Fiehn	805.562	C46H80NO8P
LysoPC(18:1(9Z))	16081932	Complex lipids	Fiehn	521.348	C26H52NO7P
biotin	171548	Primary Metabolites	Fiehn	244.088	C10H16N2O3S
2-(4-hydroxyphenyl)ethanol	10393	Primary Metabolites	Fiehn	138.068	C8H10O2
catechol	289	Primary Metabolites	Fiehn	110.037	C6H6O2
5-Dihydrocortisone [5α-PREGNAN-17,21-DIOL-3,11,20-TRIONE]	65554	Primary Metabolites	Fiehn	362.209	C21H30O5
N,N-dimethylarginine	123831	Primary Metabolites	Fiehn	202.143	C8H18N4O2
L-kynurenine	161166	Primary Metabolites	Fiehn	208.085	C10H12N2O3
salicylic acid (o-Hydroxybenzoic acid)	338	Primary Metabolites	Fiehn	138.032	C7H6O3
3-Hexenedioic acid	107550	Primary Metabolites	Fiehn	144.042	C6H8O4
4-nitroquinoline N-oxide	5955	Primary Metabolites	Fiehn	190.038	C9H6N2O3
farnesal	5280598	Primary Metabolites	Fiehn	220.183	C15H24O
DL-4-hydroxymandelic acid	328	Primary Metabolites	Fiehn	168.042	C8H8O4
1-INDANOL	22819	Primary Metabolites	Fiehn	134.073	C9H10O
oxamide	10113	Primary Metabolites	Fiehn	88.027	C2H4N2O2
10-Hydroxydecanoic acid	74300	Primary Metabolites	Fiehn	188.141	C10H20O3

Compound Name	PubChem CID	Platform	Lab	Exact Mass	Formula
2'-deoxyguanosine	187790	Primary Metabolites	Fiehn	267.097	C10H13N5O4
2,3-dihydroxybiphenyl (3-phenylcatechol)	254	Primary Metabolites	Fiehn	186.068	C12H10O2
9-fluorenone	10241	Primary Metabolites	Fiehn	180.058	C13H8O
phosphomycin	31669	Primary Metabolites	Fiehn	138.008	C3H7O4P
CHOLESTAN-3β,5α,6β-TRIOL (Cholestane-3,5,6-triol, (3.beta.,5.alpha.,6.beta.)-)	91498	Primary Metabolites	Fiehn	420.36	C27H48O3
phenanthrene	995	Primary Metabolites	Fiehn	178.078	C14H10
N-ethylglycine	316542	Primary Metabolites	Fiehn	103.063	C4H9NO2
d-Glucoheptose	76599	Primary Metabolites	Fiehn	210.074	C7H14O7
3,4-dihydroxybenzoic acid	72	Primary Metabolites	Fiehn	154.027	C7H6O4
(+/-)-Synephrine	667452	Primary Metabolites	Fiehn	167.095	C9H13NO2
synephrine	7172	Primary Metabolites	Fiehn	167.095	C9H13NO2
decanoic acid methyl ester (methyl decanoate)	8050	Primary Metabolites	Fiehn	186.162	C11H22O2
Erythrose	94176	Primary Metabolites	Fiehn	120.042	C4H8O4
citric acid	311	Primary Metabolites	Fiehn	192.027	C6H8O7
Fructose 2,6-biphosphate	105021	Primary Metabolites	Fiehn	339.996	C6H14O12P2
squalene	638072	Primary Metabolites	Fiehn	410.391	C30H50
arachidonic acid	444899	Primary Metabolites	Fiehn	304.24	C20H32O2
tocopherol acetate	86472	Primary	Fiehn	472.392	C31H52O3

Compound Name	PubChem CID	Platform	Lab	Exact Mass	Formula
		Metabolites			
tetradecanoic acid methyl ester (Methyl myristate)	31284	Primary Metabolites	Fiehn	242.225	C15H30O2
4-methylcatechol	9958	Primary Metabolites	Fiehn	124.052	C7H8O2
nicotinoylglycine	68499	Primary Metabolites	Fiehn	180.053	C8H8N2O3
halostachine	6950649	Primary Metabolites	Fiehn	151.1	C9H13NO
3,4-Dihydroxypyridine	105085	Primary Metabolites	Fiehn	111.032	C5H5NO2
saccharopine	1087	Primary Metabolites	Fiehn	276.132	C11H20N2O6
L-Saccharopine	160556	Primary Metabolites	Fiehn	276.132	C11H20N2O6
L-glutamine	5961	Primary Metabolites	Fiehn	146.069	C5H10N2O3
Allylmalonic acid	75748	Primary Metabolites	Fiehn	144.042	C6H8O4
4-hydroxy-3-methoxybenzyl alcohol	62348	Primary Metabolites	Fiehn	154.063	C8H10O3
p-anisic acid	7478	Primary Metabolites	Fiehn	152.047	C8H8O3
conduritol beta-epoxide	2859	Primary Metabolites	Fiehn	162.053	C6H10O5
methylmalonic acid	487	Primary Metabolites	Fiehn	118.027	C4H6O4
22-Ketocholesterol	101465	Primary Metabolites	Fiehn	400.334	C27H44O2
7,8-dimethylalloxazine	5326566	Primary Metabolites	Fiehn	242.08	C12H10N4O2
4-Hydroxymethyl-3-methoxyphenoxyacetic acid	134310	Primary Metabolites	Fiehn	212.068	C10H12O5
octacosanoic acid methyl ester	41518	Primary Metabolites	Fiehn	438.444	C29H58O2

Compound Name	PubChem CID	Platform	Lab	Exact Mass	Formula
aniline-o-sulfonic acid	6926	Primary Metabolites	Fiehn	173.015	C6H7NO3S
2-mercaptoethanesulfonic acid	598	Primary Metabolites	Fiehn	141.976	C2H6O3S2
indole-3-acetamide	397	Primary Metabolites	Fiehn	174.079	C10H10N2O
flavanone	10251	Primary Metabolites	Fiehn	224.084	C15H12O2
cyclic GMP	295	Primary Metabolites	Fiehn	345.047	C10H12N5O7P
Guanosine 3',5'-cyclic monophosphate	24316	Primary Metabolites	Fiehn	345.047	C10H12N5O7P
oleic acid	445639	Primary Metabolites	Fiehn	282.256	C18H34O2
Elaidic acid	637517	Primary Metabolites	Fiehn	282.256	C18H34O2
daidzein	5281708	Primary Metabolites	Fiehn	254.058	C15H10O4
butyraldehyde	261	Primary Metabolites	Fiehn	72.058	C4H8O
L-homocystine	439579	Primary Metabolites	Fiehn	268.055	C8H16N2O4S2
Homocystine	69382	Primary Metabolites	Fiehn	268.055	C8H16N2O4S2
cysteinylglycine	65270	Primary Metabolites	Fiehn	178.041	C5H10N2O3S
biphenyl	7095	Primary Metabolites	Fiehn	154.078	C12H10
PE(P-16:0/18:1(9Z))	52925128	Complex lipids	Fiehn	701.536	C39H76NO7P
2,4-dihydroxypyrimidine-5-carboxylic acid (uracil-5-carboxylic acid)	90301	Primary Metabolites	Fiehn	156.017	C5H4N2O4
