# Supplementary material for: The fermented cabbage metabolome and its protection against cytokine-induced intestinal barrier disruption of Caco-2 monolayers
Source: Appl Environ Microbiol. 2025 Apr 7;91(5):e02234-24. doi: 10.1128/aem.02234-24 (PMC12093966; doi:10.1128/aem.02234-24)
Supplement: File S2 — GC methods. [file aem.02234-24-s0002.pdf]

Data Dictionary Fiehn laboratory _NIH West Coast Metabolomics Center_09-27-2013

Primary metabolism by ALEX-CIS GCTOF MS

Glossary

ALEX automated liner exchange, produced by Gerstel corporation.
CIS cold injection system, produced by Gerstel corporation
GC gas chromatography
TOF time of flight mass spectrometer
MS mass spectrometry. After hard ionization by electron ionization, one electron gets abstracted from the intact molecules which hence become positively charged. The standardized -70 eV ionization voltage is so high that molecules fragment into multiple product ions, which may also form rearrangements among each other. Fragments are then analyzed by time of flight mass spectrometry which is made here by the vendor Leco corporation not to obtain accurate mass information at high resolution but instead to obtain mass spectra at very high sensitivity and speed.
QC quality control
IS also istd, internal standards
FAME fatty acid methyl esters
v/v volumetric ratio
InChI International Chemical Identifier key. Denotes the exact stereochemical and atomic description of chemicals and used as universal identifier in chemical databases.
KEGG Kyoto Encyclopedia of Genes and Genomes
PubChem a public database of chemicals and chemical information.
rt retention time (seconds)
RI also ret.index, retention index, a conversion of absolute retention times to relative retention times based on a set of pre-defined internal standards. Classically, Kovats retention indices are used based on hydrocarbons. We use Fiehn retention indices based on FAME istd because FAME mass spectra are much easier to correctly annotate in automatic assays.
mz also m/z, or mass-to-charge ratio. In metabolomics, ions are almost exclusively detected as singly charged species.
s/n signal to noise ratios
IUPAC International Union of Pure and Applied Chemistry
NIST National Institute of Standards and Technology
PCA Principal Component Analysis

Data acquisition

Data are acquired using the following chromatographic parameters, with more details to be found in Fiehn O. et al. Plant J. 53 (2008) 691–704.
Column: Restek corporation Rtx-5Sil MS (30 m length x 0.25 mm internal diameter with 0.25 μm film made of 95% dimethyl/5%diphenylpolysiloxane)
Mobile phase: Helium
Column temperature: 50-330°C Flow-
rate: 1 mL min^{-1}
Injection volume: 0.5 μL
Injection: 25 splitless time into a multi-baffled glass liner
Injection temperature: 50°C ramped to 250°C by 12°C s^{-1}
Oven temperature program: 50°C for 1 min, then ramped at 20°C min^{-1} to 330°C, held constant for 5 min.

The analytical GC column is protected by a 10 m long empty guard column which is cut by 20 cm intervals whenever the reference mixture QC samples indicate problems caused by column contaminations. We have validated that at this sequence of column cuts, no detrimental effects are detected with respect to peak shapes, absolute or relative metabolite retention times or reproducibility of quantifications. This chromatography method yields excellent retention and separation of primary metabolite classes (amino acids, hydroxyl acids, carbohydrates, sugar acids, sterols, aromatics, nucleosides, amines and miscellaneous compounds) with narrow peak widths of 2–3 s and very good within-series retention time reproducibility of better than 0.2 s absolute deviation of retention times. We use automatic liner exchanges after each set of 10 injections which we could show to reduce sample carryover for highly lipophilic compounds such as free fatty acids.
Mass spectrometry parameters are used as follows: a Leco Pegasus IV mass spectrometer is used with unit mass resolution at 17 spectra s^{-1} from 80-500 Da at -70 eV ionization energy and 1800 V detector voltage with a 230°C transfer line and a 250°C ion source.

Data processing

Raw data files are preprocessed directly after data acquisition and stored as ChromaTOF-specific *.peg files, as generic *.txt result files and additionally as generic ANDI MS *.cdf files. ChromaTOF vs. 2.32 is used for data preprocessing without smoothing, 3 s peak width, baseline subtraction just above the noise level, and automatic mass spectral deconvolution and peak detection at signal/noise levels of 5:1 throughout the chromatogram. Apex masses are reported for use in the BinBase algorithm. Result *.txt files are exported to a data server with absolute spectra intensities and further processed by a filtering algorithm implemented in the metabolomics BinBase database.

The BinBase algorithm (rtx5) used the settings: validity of chromatogram (<10 peaks with intensity >10^7 counts s^{-1}), unbiased retention index marker detection (MS similarity>800, validity of intensity range for high m/z marker ions), retention index calculation by 5th order polynomial regression. Spectra are cut to 5% base peak abundance and matched to database entries from most to least abundant spectra using the following matching filters: retention index window ±2,000 units (equivalent to about ±2 s retention time), validation of unique ions and apex masses (unique ion must be included in apexing masses and present at >3% of base peak abundance), mass spectrum similarity must fit criteria dependent on peak purity and signal/noise ratios and a final isomer filter. Failed spectra are automatically entered as new database entries if s/n >25, purity <1.0 and presence in the biological study design class was >80%. All thresholds reflect settings for ChromaTOF v. 2.32. Quantification is reported as peak height using the unique ion as default, unless a different quantification ion is manually set in the BinBase administration software BinView. A quantification report table is produced for all database entries that are positively detected in more than 10% of the samples of a study design class (as defined in the miniX database) for unidentified metabolites. A subsequent post-processing module is employed to automatically replace missing values from the *.cdf files. Replaced values are labeled as 'low confidence' by color coding, and for each metabolite, the number of high-confidence peak detections is recorded as well as the ratio of the average height of replaced values to high-confidence peak detections. These ratios and numbers are used for manual curation of automatic report data sets to data sets released for submission.

Data reporting

Data are reported including metadata, see example below.

							Subject ID	223913	157819	124940
							Local code	A0050702A	A0125621A	142363
							Vial Barcode	1RAR7	1GZR9	1AN1N
							Date received	4-Dec-12	4-Dec-12	4-Dec-12
							Date of evalu	3/17/2013	3/17/2013	3/17/2013
							Sample Status			
							REVISION			
							Comments	GCTOF MS_s	GCTOF MS_sa	GCTOF MS_s
							Acq. Time	5:18:33 AM	5:43:50 AM	6:09:05 AM
							Data File Nam	130328cmss	130328cmss	130328cms
							miniX id	118072	118073	118074
**BinBase i**	BinBase name	ret.index	quant mz	mass spec	InChI key	KEGG id	PubChem id			
14441	z C30 FAME internal stand	1113100	87	82:386.0 83:	BIRUBGLRQLAEFF-UHFFF	n/a	12400	16026	15203	18096
14378	z C28 FAME internal stand	1061700	87	82:1635.0 83	ZKHOYAKAFALNQD-UHFF	n/a	41518	39317	228	11145
14367	z C26 FAME internal stand	1006900	87	82:1915.0 83	VHUJBYYFFWDLNM-UHFF	n/a	22048	32809	30571	35507
14373	z C24 FAME internal stand	948820	87	82:1153.0 83	XUDJZDNUVZHSKZ-UHFFF	n/a	75546	43836	43163	48731
14350	z C22 FAME internal stand	886620	87	82:3004.0 83	QSQLTHHMFHEFIY-UHFF	n/a	13584	53566	51781	58740
14338	z C20 FAME internal stand	819620	87	82:5074.0 83	QGBRLVONZXHAKJ-UHFF	n/a	14259	57778	58877	63250
14344	z C18 FAME internal stand	747420	87	82:1871.0 83	HPEUJPJOZXNMSJ-UHFFFA	n/a	8201	53466	52542	57091
14328	z C16 FAME internal stand	668720	87	82:7874.0 83	FLIACVVOZYBSBS-UHFFF	C16995	8181	170150	166843	186189
14330	z C14 FAME internal stand	582620	87	82:6846.0 83	ZAZKJZBWRNNLDS-UHFF	n/a	31284	117029	113463	131850
15538	z C12 FAME internal stand	487220	87	87:89125.0 1	UQDUPQYQJKYHQI-UHFFF	n/a	8139	147184	142864	169095
14348	z C10 FAME internal stand	381020	87	82:2900.0 83	YRHYCMZPEVDGFQ-UHFF	n/a	8050	131518	127669	150456
14356	z C09 FAME internal stand	323120	87	82:1461.0 83	IJXHLVMUNBOGRR-UHFF	n/a	15606	119374	119013	137532
14391	z C08 FAME internal stand	262320	87	82:816.0 83:	JGHZJRVDZXSNKQ-UHFFF	n/a	8091	79355	87424	91461
231968	xylose	544673	103	85:5068.0 86	PYMYPHUHKUWMLA-VPE	C02205	644160	19097	1462	1642
368041	xylitol	566570	217	86:588.0 87:	HEBKCHPVOIAQTA-NGQZ	C00379	6912	239	168	121
203224	xanthine	702391	353	85:1361.0 86	LRFVTYWOQMYALW-UHF	C00385	1188	64	26	60
199605	valine	313224	144	85:48.0 86:1	KZSNJWFQEVHDMF-BYPY	C00183	6287	108089	120432	133290
213127	uridine	856953	258	85:2472.0 86	DRTQHJPVMGBUCF-XVFC	C00299	6029	256	63	60
304993	uric acid	730534	441	85:1183.0 86	LEHOTFFKMJEONL-UHFF	C00366	1175	16528	13715	7856
224322	urea	337230	171	87:1643.0 89	XSQUKJJJFZCRTK-UHFFFA	C00086	1176	334000	281631	313888

The **'BinBase identifier column'** denotes the unique identifier for the GCTOFMS platform. It is given for both identified and unidentified metabolites in the same manner.

The **'BinBase name'** denotes the name of the metabolite, if the peak has been identified. A chemical name is not a unique identifier. We use names recognized by biologists instead of IUPAC nomenclature. If a compound is identified, it has a name, and external database identifiers such as InChI key, PubChem ID and KEGG ID. If a compound is unknown, the name is the same as given in the 'identifier column'.

The **'retention index'** column details the target retention index in the BinBase database system.
The **'quant mz'** column details the m/z value that was used to quantify the peak height of a BinBase entry.
The **'mass spec'** column details the complete mass spectrum of the metabolite given as mz: intensity values, separated by spaces.
The **'InChI key'** identifier gives the unique chemical identifier defined by the IUPAC and NIST consortia.
The **'KEGG'** identifier gives the unique identifier associated with an identified metabolite in the community database KEGG LIGAND DB.
The **'PubChem'** column denotes the unique identifier of a metabolite in the PubChem database.
The **'internal standard'** addition within the BinBase name clarifies if a specific chemical has been added into the extraction solvent as internal standard. These internal standards serve as retention time alignment markers, for quality control purposes and for quantification corrections.

Row metadata that are requested by a specific consortium are labeled in blue.
Consortium **'subject ID', 'local ID', 'vial barcode'** detail information given by a specific consortium.
The row '**date received'** is the date when samples were received in the metabolomics laboratory.
The row '**date of evaluation'** is the data of data acquisition, as given by the machine logbook.
The row '**sample status'** uses the consortium's sample status code if samples have errors. The consortium sample status code does not give a code when data acquisition occurred without problems. If a consortium does not use an authorized error code dictionary, plain text is given for errors.
The row '**revision'** details if data processing yields a new data sheet. Data revisions may be needed when new algorithms have been tested, validated and deployed that might yield better raw data analyses than prior submissions. By default, therefore, data revisions replace the (less valid) prior data submissions. However, data revisions may also indicate a different form of data treatment, e.g. data normalizations (see below). In this case, the 'revision' would indicate the type of normalization.
Any information in the row 'revision' will have a date stamp when the revision was conducted in the form of *MMDDYY*.
The **'comments'** row gives comments about the platform and type of sample. A sample is given as "sample" in comparison to e.g. a quality control or a blank injection.
The '**Acq.Date-Time'** row details the acquisition time when the data acquisition was completed.
The **'Data File Name'** row denotes the name of the raw data file. Raw data files are secured at the NIH Metabolomics database, www.metabolomicsworkbench.org
Data file names are dictated by the laboratory's information and management system when the sequence starts running. GCTOF raw file names from the Leco instrumentation end with .peg (this ending is not given in the file name, but is found in the database repositories).
In case a sample will need to be reinjected, the file name will change from e.g. 130328cmssa40_1.peg to 130328cmssa40 _2.d for the second injection, 130328cmssa40_3.d for the third injection and subsequent injections. The file name itself denotes YYMMDD then the 'machine used for data acquisition' (here: c; we have four GCTOF MS machines a-d), 'person who operated the machine' (here: ms for Mimi Swe), 'sa' for sample (instead of e.g. 'qc' for a quality control or 'bl' for a blank sample), followed by the sequence number (here: the 40th sample within the sample sequence).
The '**miniX'** row shows the unique sample identifier in the Fiehnlab miniX laboratory information management system.

The **actual data** are given as peak heights for the quantification ion (mz value) at the specific retention index. We give peak heights instead of peak areas because peak heights are more precise for low abundant metabolites than peak areas, due to the larger influence of baseline determinations on areas

compared to peak heights. Also, overlapping (co-eluting) ions or peaks are harder to deconvolute in terms of precise determinations of peak areas than peak heights. Such data files are then called 'raw results data' in comparison to the raw data file produced during data acquisition (see 'data file name'). The worksheets are called 'Height'.

Raw results data need to be normalized to reduce the impact of between-series drifts of instrument sensitivity, caused by machine maintenance, aging and tuning parameters. Such normalization data sets are called 'norm data' worksheets.

There are many different types of normalizations in the scientific literature. We usually provide first a variant of a 'vector normalization' in which we calculate the sum of all peak heights for all identified metabolites (but not the unknowns!) for each sample. We call such peak-sums "mTIC" in analogy to the term TIC used in mass spectrometry (for 'total ion chromatogram'), but with the notification "mTIC" to indicate that we only use genuine metabolites (identified compounds) in order to avoid using potential non-biological artifacts for the biological normalizations, such as column bleed, plasticizers or other contaminants.

Subsequently, we determine if the mTIC averages are significantly different between treatment groups or cohorts. If these averages indeed are different by $p<0.05$, data will be normalized to the average mTIC of each group. If averages between treatment groups or cohorts are not different, or if treatment relations to groups are kept blinded, data will be normalized to the total average mTIC.

Following equation is then used for normalizations for metabolite *i* of sample *j*:

$$\mathrm{metabolite}_{\mathrm{ij,\ normalized}} = \frac{\mathrm{metabolite}_{\mathrm{ij,\ raw}}}{\mathrm{mTIC}_{\mathrm{j}}} \cdot \mathrm{mTIC}_{\mathrm{average}}$$

The worksheet is then called **'norm mTIC'**. Data are 'relative semi-quantifications', meaning they are normalized peak heights. Because the average mTIC will be different between series of analyses that are weeks or months apart (due to differences in machine sensitivity, tuning, maintenance status and other parameters), **additional normalizations** need to be performed. For this purpose, identical samples ('QC samples') must be analyzed multiple times in all series of data acquisitions. In fact, one must not exclude the possibility that even within a series of data acquisitions, a sensitivity shift or drift might occur. Hence, the following statistical analyses are suggested: (a) compute univariate statistics for mTIC values in batches within-series and between-series of data injections, using time/date stamps to find potential breaks during which machine downtime may have occurred. If there are no mTIC differences between such time/date stamp batches, calculate an overall mTIC covering all samples. (b) compute multivariate PCA plots for the , marking the potentially different samples of individual time/date stamp batches using different colors. If there is no apparent separation between PCA clusters of different colors, there is no large between-series effect and these PCA clusters can be treated as indistinguishable. If there is suspicion of hidden features that might be masked by overall variance analysis in PCA, supervised statistics by Partial Least Square regression models can unravel such between-series differences.

Once different clusters (i.e. series of undistinguishable QC samples) have been identified, correction factor models need to be developed that correct differences between those QC samples. Subsequently, these correction factors can be applied to the actual analytical samples to remove overt quantification differences that are not related to biological causes but solely due to analytical errors.

Such correction factor models can be computed in different ways, e.g. by unit-variance mean centering or by calculating simple offset vectors for each individual metabolite. The best way of such types of normalizations is being explored in the Fiehn laboratory. However, in any case, such correction models

can only be developed if a sufficient number of QC samples have been included in the analytical sequences. For that reason, the Fiehn laboratory uses a suitable QC sample for every 11th injection. Such QC samples need to be as similar to the actual biological specimen as possible, e.g. generated by pool samples during extractions or by obtaining typical community standard samples (e.g. the NIST standard blood plasma, or commercial serum or plasma samples as needed).

If appropriate internal standards are used for absolute quantifications, the following equation could be used for peak height normalizations for metabolite *i* of sample *j* and internal standard *k*

$$\text{metabolite}_{\text{ij, normalized}} = \frac{\text{metabolite}_{\text{ij, raw}}}{\text{istd}_{\text{k}}} \cdot \text{concentration istd}_{\text{k}}$$

However, there are few universal or class-specific internal standards in GC-MS based analysis, because within each chemical class, metabolites may have drastically different calibration curves (sensitivity or 'response') based on a combination of injection, volatilization and stability and ionization response properties. As surrogate, external calibration standards could be used for specific (important) metabolites which, however, cannot be applied for unidentified compounds and which of course would not account for recovery during extraction procedures.
