# Supplementary material for: The fermented cabbage metabolome and its protection against cytokine-induced intestinal barrier disruption of Caco-2 monolayers
Source: Appl Environ Microbiol. 2025 Apr 7;91(5):e02234-24. doi: 10.1128/aem.02234-24 (PMC12093966; doi:10.1128/aem.02234-24)
Supplement: Supplemental figures — Figures S1 to S7 and legends for Files S1 to S5. [file aem.02234-24-s0006.docx]

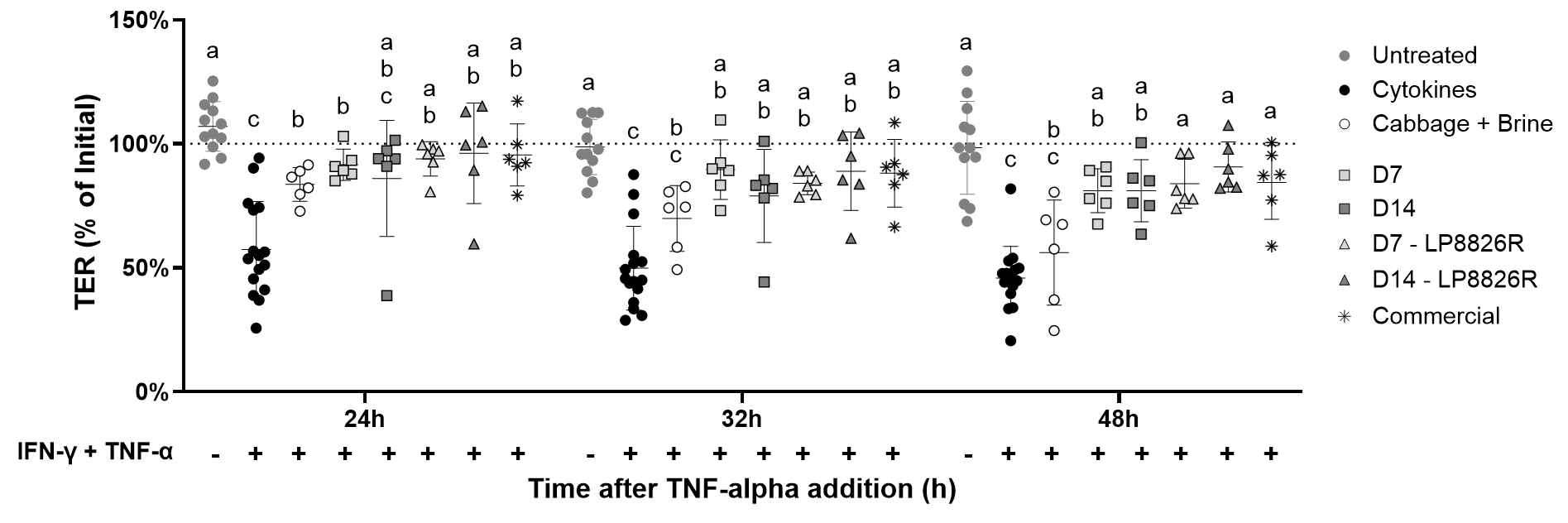


**Figure S1. Effect of (fermented) cabbage homogenates on intestinal barrier permeability of cytokine-perturbed Caco-2 monolayers.** Trans-epithelial electrical resistance (TER) of Caco-2 monolayers 24, 32, and 48 h after basolateral TNF-α addition. Values are normalized to TER immediately prior to when TNF-α was added. D7 and D14 were collected on days 7 and 14, respectively, from LSF either with or without the LP8826R additions. Replicates included Caco-2 monolayers not exposed to IFN-γ and TNF-α (untreated) (n = 12), controls to which the cytokines were applied (n = 16), and those exposed to the cytokines and (fermented) cabbage (n = 6). The mean ± SD is shown for four independent experiments. The letters indicate significant differences based on two-way ANOVA with Tukey’s multiple comparisons test.


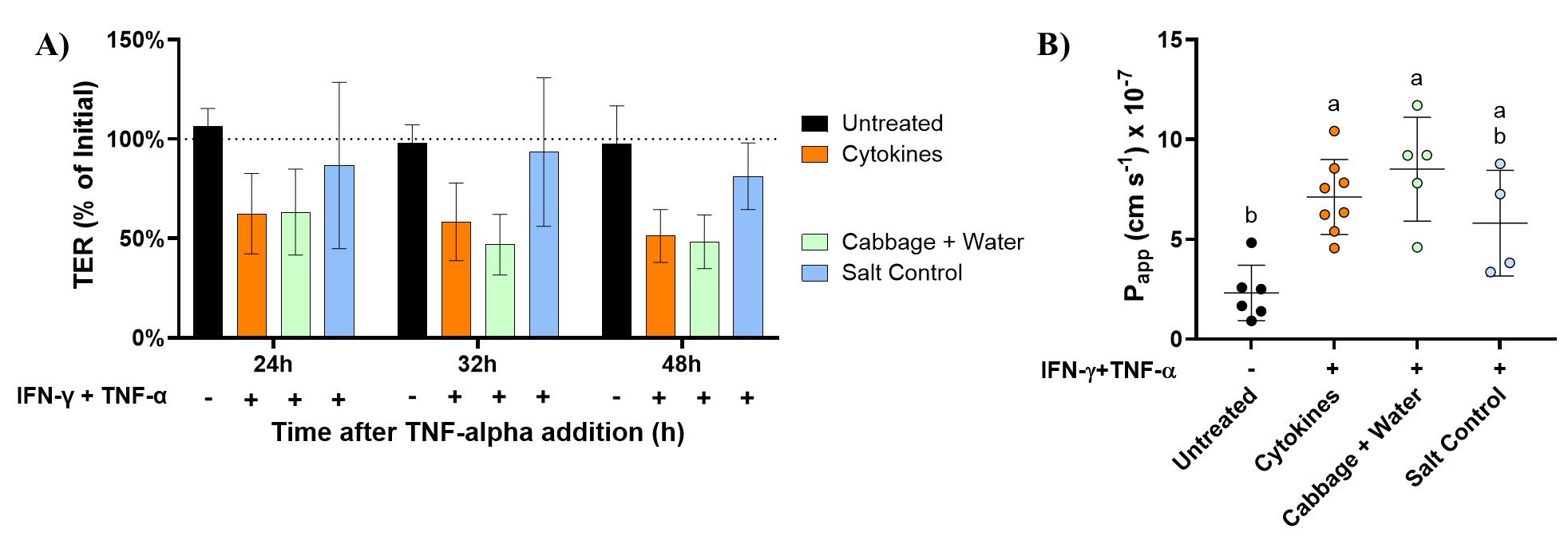


**Figure S2. Effect of salt control on intestinal barrier permeability of cytokine-perturbed Caco-2 monolayers.** (**A**) Trans-epithelial electrical resistance (TER) of Caco-2 monolayers 24, 32, and 48 h after basolateral TNF-α (10 ng/ml) addition. Values are normalized to TER immediately prior to when TNF-α was added. (**B**) Apparent permeability coefficient (P_app_) to FITC-dextran for Caco-2 monolayers at the 48 h time point. Replicates included Caco-2 monolayers not exposed to IFN-γ and TNF-α (n = 5), controls to which the cytokines were applied (n = 7), and those exposed to the cytokines and cabbage + water homogenate or salt control (2.91 ppt salinity) (at least n = 3). The mean ± SD is shown for two independent experiments. The asterisks and letters indicate significant differences based on two-way or one-way ANOVA with Tukey’s multiple comparisons test, respectively.

**
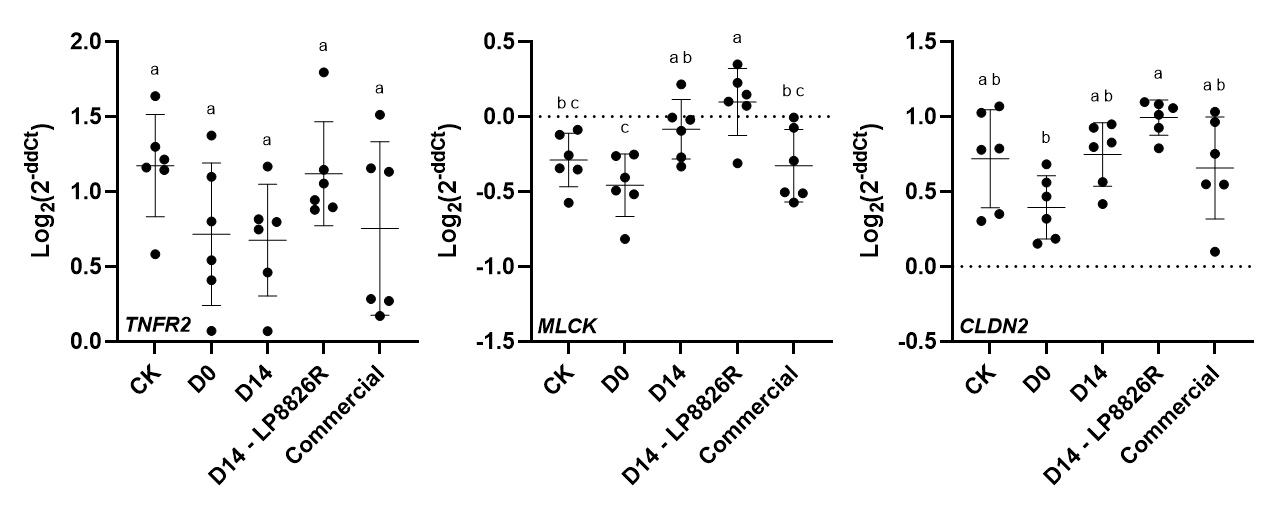
**

**Figure S3. Transcript levels of *TNFR2*, *MLCK*, and *CLDN2* in Caco-2 monolayers after sequential exposure to IFN-γ and TNF-α.** Cells were collected 48 h after TNF-α exposure. The results are normalized to Caco-2 monolayers not exposed to IFN-γ and TNF-α using the 2^-ΔΔCt^ method with *GAPDH* as the housekeeping gene. Caco-2 monolayers were exposed to the IFN-γ and TNF-α cytokines (CK) and CK and (fermented) cabbage homogenates from the start (D0) of the study and day 14 (D14) of LSF without (D14) and with LP8896R (D14-LP8826R) and commercial ferments. The mean ± SD is shown for n = 6 replicates. The mean values that do not share any letters were significantly different based on Kruskal-Wallis with Dunn’s multiple comparisons test.


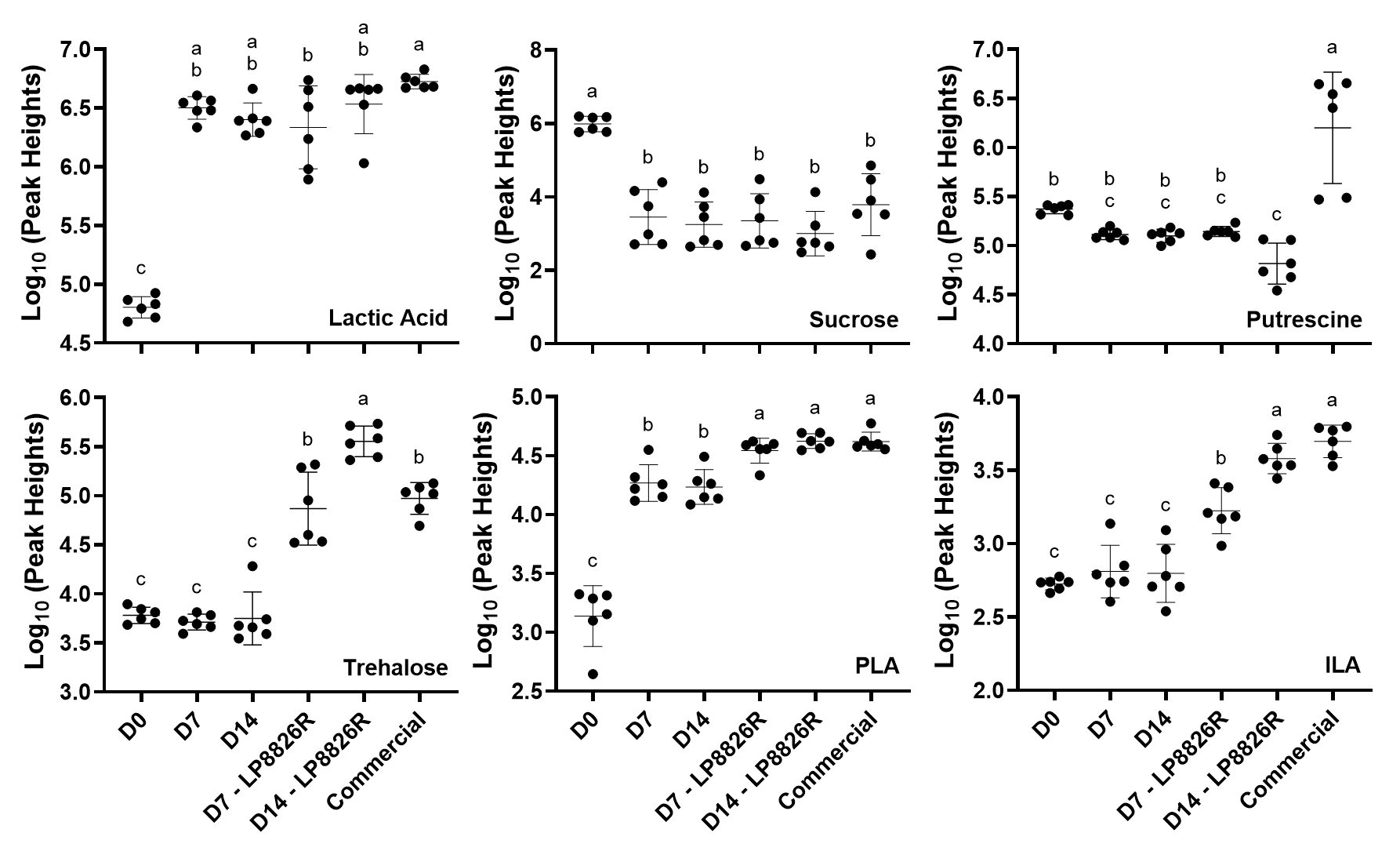


**Figure S4. Representative metabolites detected by GC-TOF/MS.** Metabolites were detected in homogenates of cabbage and brine from day 0 (D0), day 7 laboratory scale ferments (LSFs) without (D7) and with LP8826R (D7 – LP8826R), day 14 LSFs without (D14) and with LP8826R (D14 – LP8826R), and commercial ferments. Mean values not sharing letter are significantly different based on one-way ANOVA with Tukey’s multiple comparisons test. Mean ± SD for n = 6 replicates. PLA: phenyl-lactic acid. ILA: indole-3-lactate.

**
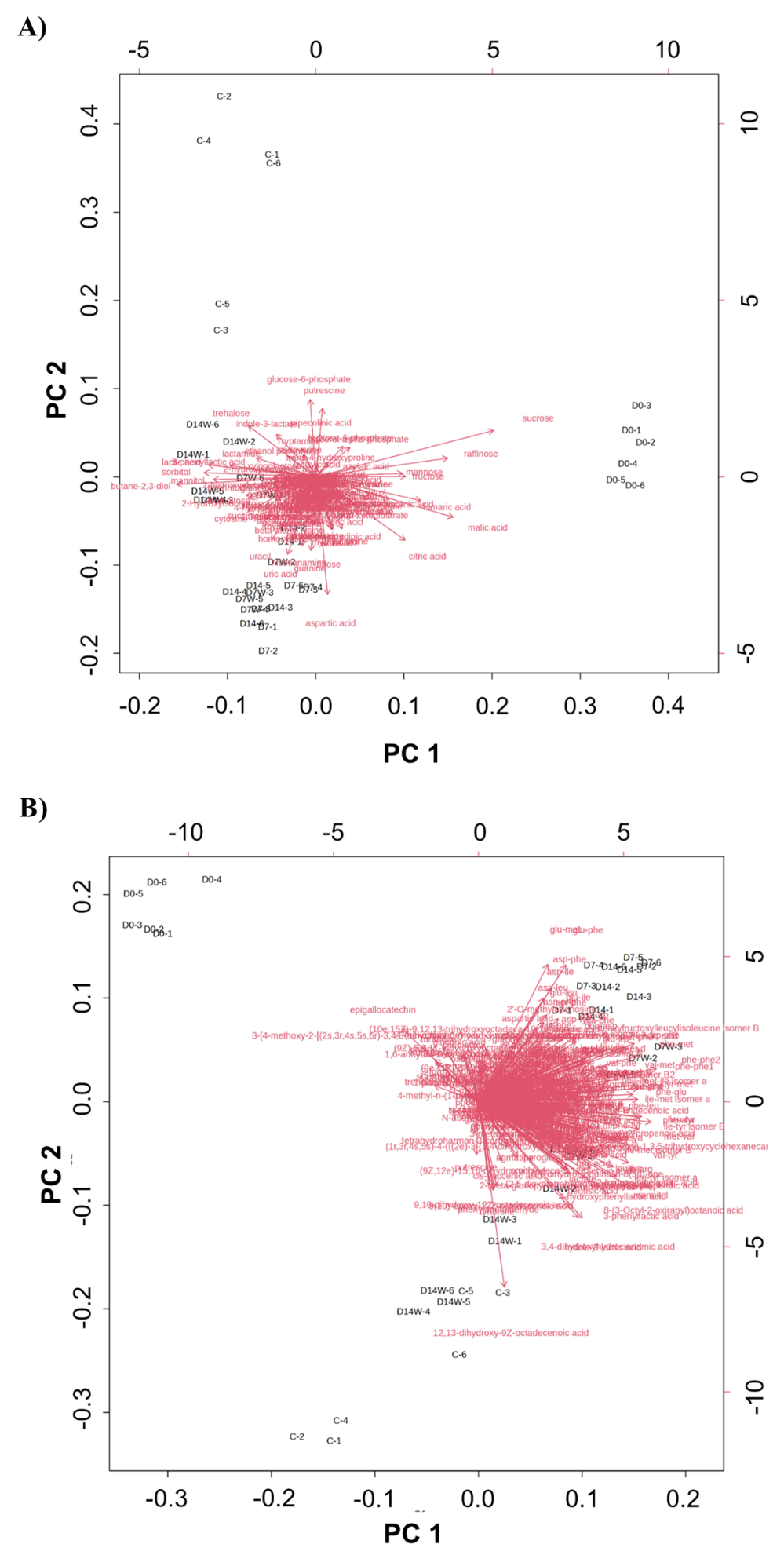
**

**Figure S5. Principal component analysis (PCA) biplots showing correlations between metabolites and (fermented) cabbage homogenates.** Metabolites identified through untargeted (**A**) GC-TOF/MS or (**B**) RP-LC-HRMS/MS and homogenates that are spatially in the same direction along an axis suggest positive correlations; metabolites and homogenates located in different directions indicate no correlations. The strength of the correlation is shown by the increasing distance from the origin of the plot.

**
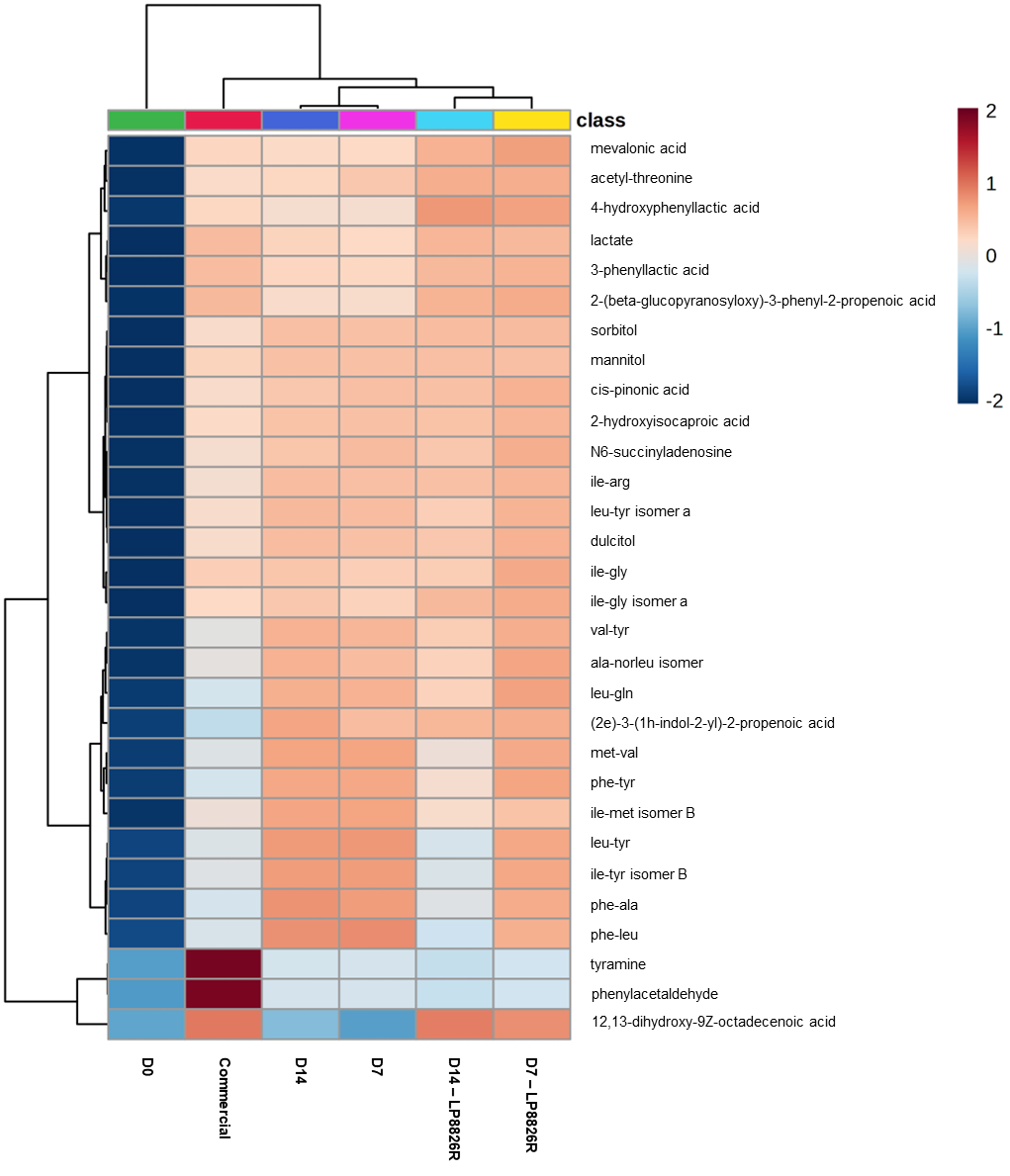
**

**Figure S6. Heatmap of RP-LC-HRMS/MS metabolites found in significantly different levels between the (fermented) cabbage homogenates.** The heatmap shows the relative peak heights of the top 35 metabolites (with the lowest p values) identified through RP-LC-HRMS/MS. Visualization of the compounds were created using normalized data. The blue color represents lower relative peak heights while red color represents higher relative peak heights. Metabolite features were standardized through autoscaling (z-score = subtract mean and divide by standard deviation). Similarity between data points was assessed using Euclidean distance measure and then hierarchically clustered using Ward’s method. Top 35 features with the lowest p values were determined by one-way ANOVA with Tukey’s multiple comparisons test.

**
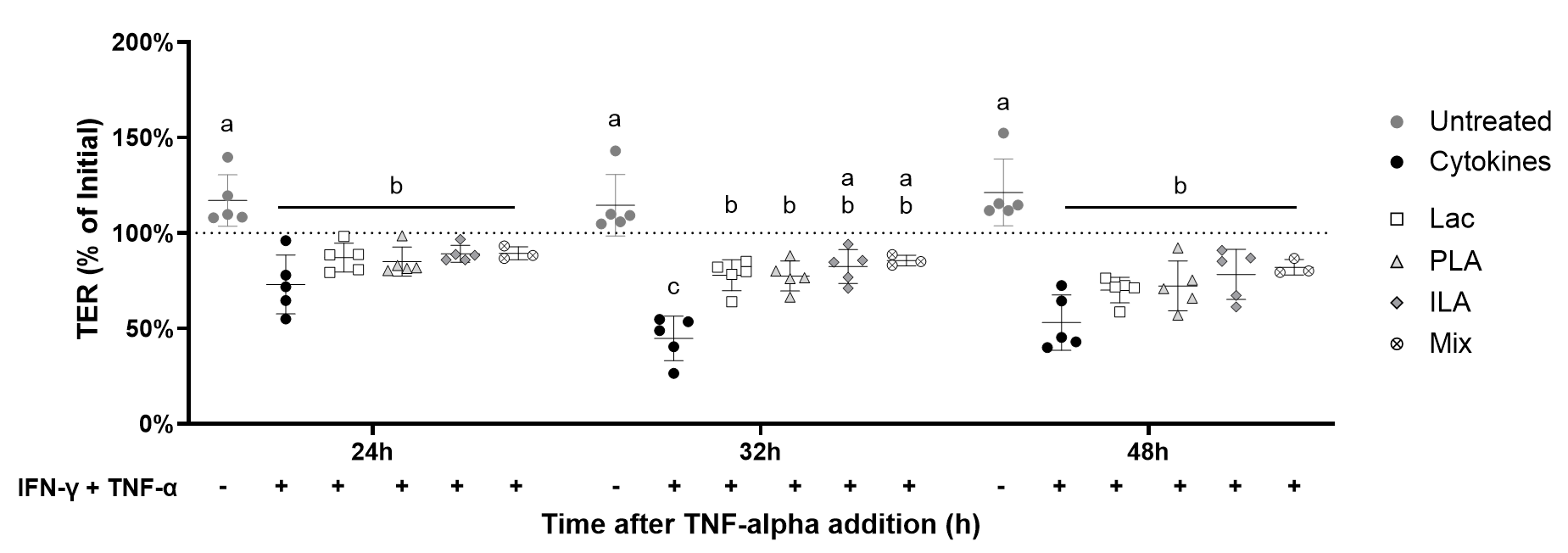
**

**Figure S7. Effect of lactate, PLA, ILA, and their mixture on intestinal barrier permeability of cytokine-perturbed Caco-2 monolayers.** Trans-epithelial electrical resistance (TER) of Caco-2 monolayers 24, 32, and 48 h after basolateral TNF-α (10 ng/ml) addition. Values are normalized to TER immediately prior to when TNF-α was added. Replicates included Caco-2 monolayers not exposed to IFN-γ and TNF-α (Untreated) (n = 5), controls to which the cytokines were applied (Cytokines) (n = 5), and those exposed to the cytokines and metabolite, specifically 50 mM lactate (Lac), 60 µM D-phenyl-lactate (PLA), 25 µM indole-3-lactate (ILA), or a mixture of the three (Mix) (at least n = 3). The mean ± SD is shown. The letters indicate significant differences based on two-way ANOVA with Tukey’s multiple comparisons test.

**Supplemental File Legends**

**Supplemental File 1.** List of metabolites detected by the West Coast Metabolomics Center platforms (as of August 2024).

**Supplemental File 2.** West Coast Metabolomics Center Fiehn Laboratory – Information on data acquisition, processing, and raw data normalization.

**Supplemental File 3.** Peak heights of internal standards used for untargeted LC-RP-HRMS/MS.

**Supplemental File 4.** List of GC-TOF/MS metabolites significantly changed in each cabbage ferment group compared to day 0.

**Supplemental File 5.** List of RP-LC-HRMS/MS metabolites significantly changed in each cabbage ferment group compared to day 0.
